# Human Milk Bioactive Components and Child Growth and Body Composition in the First 2 Years: A Systematic Review

**DOI:** 10.1016/j.advnut.2023.09.015

**Published:** 2023-10-04

**Authors:** Meredith (Merilee) Brockway, Allison I. Daniel, Sarah M. Reyes, Julia M. Gauglitz, Matthew Granger, Joann M. McDermid, Deborah Chan, Rebecca Refvik, Karanbir K. Sidhu, Suad Musse, Pooja P. Patel, Caroline Monnin, Larisa Lotoski, Donna T. Geddes, Fyezah Jehan, Patrick Kolsteren, Lars Bode, Kamilla G. Eriksen, Lindsay H. Allen, Daniela Hampel, Natalie Rodriguez, Meghan B. Azad

**Affiliations:** 1 Manitoba Interdisciplinary Lactation Centre (MILC), Children’s Hospital Research Institute of Manitoba, University of Manitoba, Winnipeg, MB, Canada; 2 Department of Pediatrics and Child Health, University of Manitoba, Winnipeg, MB, Canada; 3 Faculty of Nursing, University of Calgary, Calgary, AB, Canada; 4 Department of Nutritional Sciences, University of Toronto, Toronto, ON, Canada; 5 Centre for Global Child Health, Hospital for Sick Children, Toronto, ON, Canada; 6 Sapient Bioanalytics LLC, San Diego, CA, United States; 7 Department of Food and Human Nutritional Sciences, University of Manitoba, Winnipeg, MB, Canada; 8 Consultant, Charlottesville, VA, United States; 9 Department of Epidemiology, Biostatistics, and Occupational Health, McGill University, Montréal, QC, Canada; 10 Department of Public Health and Community Medicine, Tufts University School of Medicine, Boston, MA, Unites States; 11 Neil John Maclean Health Sciences Library, University of Manitoba, Winnipeg, MB, Canada; 12 School of Molecular Sciences, The University of Western Australia, Perth, WA, Australia; 13 Department of Pediatrics & Child Health, Aga Khan University, Karachi, Pakistan; 14 Department of Food Safety and Food Quality, Ghent University, Ghent, Belgium; 15 Department of Pediatrics, Mother-Milk-Infant Center of Research Excellence (MOMI CORE), University of California, San Diego (UC San Diego), San Diego, CA, United States; 16 Department of Nutrition, Exercise and Sports, Faculty of Science, University of Copenhagen, Copenhagen, Denmark; 17 Department of Nutrition, University of California, Davis, CA, United States; 18 Western Human Nutrition Research Center, Agriculture Research Service, United States Department of Agriculture, Washington, DC, Unites States

**Keywords:** human milk, breastmilk, breastfeeding, infant, anthropometry, bioactives, hormones, human milk oligosaccharides, immunomodulatory, metabolomics, body composition, growth, lactation

## Abstract

Human milk (HM) contains macronutrients, micronutrients, and a multitude of other bioactive factors, which can have a long-term impact on infant growth and development. We systematically searched MEDLINE, EMBASE, Cochrane Library, Scopus, and Web of Science to synthesize evidence published between 1980 and 2022 on HM components and anthropometry through 2 y of age among term-born infants. From 9992 abstracts screened, 141 articles were included and categorized based on their reporting of HM micronutrients, macronutrients, or bioactive components. Bioactives including hormones, HM oligosaccharides (HMOs), and immunomodulatory components are reported here, based on 75 articles from 69 unique studies reporting observations from 9980 dyads. Research designs, milk collection strategies, sampling times, geographic and socioeconomic settings, reporting practices, and outcomes varied considerably. Meta-analyses were not possible because data collection times and reporting were inconsistent among the studies included. Few measured infant HM intake, adjusted for confounders, precisely captured breastfeeding exclusivity, or adequately described HM collection protocols. Only 5 studies (6%) had high overall quality scores. Hormones were the most extensively examined bioactive with 46 articles (*n =* 6773 dyads), compared with 13 (*n =* 2640 dyads) for HMOs and 12 (*n =* 1422 dyads) for immunomodulatory components. Two studies conducted untargeted metabolomics. Leptin and adiponectin demonstrated inverse associations with infant growth, although several studies found no associations. No consistent associations were found between individual HMOs and infant growth outcomes. Among immunomodulatory components in HM, IL-6 demonstrated inverse relationships with infant growth. Current research on HM bioactives is largely inconclusive and is insufficient to address the complex composition of HM. Future research should ideally capture HM intake, use biologically relevant anthropometrics, and integrate components across categories, embracing a systems biology approach to better understand how HM components work independently and synergistically to influence infant growth.

**Statement of Significance**

Our work comprehensively synthesizes evidence regarding associations between individual human milk bioactives and child anthropometrics among healthy, term-born infants. This manuscript is part of a larger three-part systematic review (PROSPERO: CRD42020187350).

## Introduction

Beyond providing a custom-made source of micronutrients and macronutrients, human milk (HM) contains a multitude of other bioactive factors [1]. Together, these components create a biologically active system to meet the health and nutritional needs of infants and young children. The WHO recommends exclusive breastfeeding for the first 6 mo and continued HM feeding as a component of the diet through 2 y and beyond [2]. HM bioactives can have a prolonged impact on the infant microbiome, growth and development, as well as immune function [3]. Although infant formula provides a safe nutrient substitute for HM, it is deficient in its capacity to replace HM bioactives. Despite decades of research, we still have a limited understanding of how many HM components inform the most fundamental of infant outcomes, including growth and development. However, as technology improves, there has been an increasing push to expand the breadth and scope of HM composition research, moving beyond nutrient analysis to investigate diverse components and clinical outcomes in the infant.

Anthropometry is a primary indicator of health for physicians, care providers, and parents. In high-resourced countries, a key goal is to prevent childhood obesity and identify risk factors or predictors of obesity in early life [4]. Conversely, lower resourced settings often have a greater need to understand and mitigate concerns around child under-nutrition and stunting [5]. Investigating how HM components contribute to infant anthropometry in healthy full-term infants, will provide a broad group of researchers and clinicians with an enhanced understanding of the role that HM feeding plays in child growth. This in turn will help to provide improved evidence to inform practice recommendations, and health promotion strategies to support breastfeeding and optimal infant growth and will assist the industry to better design HM alternatives when HM is not available.

The aim of this systematic review was to assess and synthesize evidence on the associations between HM components and child anthropometry measured in the first 2 y. Because of a large number of articles retrieved, results were organized into 3 manuscripts encompassing the following categories: micronutrients (vitamins and minerals [6]), macronutrients (lipids, proteins, and digestible carbohydrates [7]), and the current manuscript which examines bioactive components (for example, cytokines, hormones, and non-digestible carbohydrates). Maternal cells, ribonucleic acid, and microbiota were excluded from this review.

### Bioactive components in HM

Bioactive components of HM are defined as components that “affect biological processes or substrates and hence have an impact on body function or condition and ultimately health” [1]. Bioactive components in HM include lactoferrin, growth factors, hormones, nucleotides, human milk oligosaccharides (HMOs), immunoglobulins, and cytokines [[8], [9]]. Although this list is not exhaustive, for the purposes of this review, we have classified bioactives in HM into 3 categories: hormones, HMOs, and immunomodulatory components.

Hormones enter milk from the maternal bloodstream and are produced endogenously in the maternal epithelium of the mammary gland [10]. Insulin, ghrelin, adiponectin, and leptin are commonly examined for their appetite-regulating functions [10] and are among the most extensively studied bioactive components in HM in relation to infant growth [10]. Previous research examining the link between hormones and infant growth has reported conflicting results [10].

HMOs are the third most abundant component in HM. HMOs are complex carbohydrates that are indigestible to the infant yet serve as prebiotics for commensal bacteria in the infant’s gut [11], thus impacting the infant microbiome. HMOs can also have anti-adhesive functions, sequester pathogens, and directly interact with the gut epithelium and immune cells [12]. Furthermore, HMOs are minimally absorbed into the infant circulation where they can have systemic effects [13]. The most influential predictor of HMO composition is maternal secretor status, which is determined by a single nucleotide polymorphism on the fucosyltransferase 2 (FUT2) gene [14]. HM produced by secretors contains HMOs that have α1,2-fucosylated oligosaccharides, whereas milk produced by nonsecretors is deficient in this class of HMOs. Although relatively little research has addressed the impact of HMOs or maternal secretor status on infant growth, some commercial formulas are now adding HMOs to their products, often including or limited to α1,2-fucosylated HMOs that are not produced by non-secretor mothers [[11], [15]].

HM contains multiple components that impact the infant’s immune system. These include cytokines, growth factors, lactoferrin, lysozyme, and immunoglobulins [16]. Although each of these factors has a different pathway of impact, they all influence the development and function of the immune system and help provide immunity for the breastfed infant during a critical period when the infant’s own immune system is developing [17]. Although they are best known for their immunomodulating properties, it is conceivable that these factors could also influence infant growth—either by supporting optimal immune health or via immune-independent mechanisms such as energy spared by preventing illness in the infant.

## Methods

This review was registered with PROSPERO: CRD42020187350 [6] and is reported according to the PRISMA [18]. Nine reviewers (SMR, JMM, DC, MG, KS, SM, PPP, RR, and MB) independently participated in abstract and full-text screening, quality assessment, and data extraction. Covidence Systematic Review Software (2020) was used to manage screening and data extraction.

### Search strategy and screening

In consultation with the review team, a health sciences librarian (CM) developed and tested the search strategy. Using a combination of controlled vocabulary and keywords to create search concepts for HM, growth and development, macronutrients, micronutrients, and bioactive components. We also included an infant search filter adapted from the Pediatric Search Filter from the Cochrane Childhood Group to limit ≤ 24 mo of age [19]. The search was peer-reviewed by another health sciences librarian using the Peer Review of Electronic Search Strategies method [20]. The original search strategy was created in MEDLINE (Ovid) and translated to the other databases. The MEDLINE (Ovid) strategy is available in Appendix A. All other strategies are available upon request.

We searched the following databases in March 2020: MEDLINE (Ovid; MEDLINE® All 1946–2020), EMBASE (Ovid; 1974–2020), the Cochrane Library (Wiley; CENTRAL and Cochrane Database of Systematic Reviews), Scopus (1970–2020), and Web of Science Core Collection (Clarivate, 1900–2020). To locate grey literature we searched Agricola, Practice-based Evidence in Nutrition (PEN®), OpenSIGLE, Google Advanced, and PROSPERO. These resources were selected to ensure the retrieval of materials relevant to nutrition, food science, and technology. Finally, we conducted reverse snowballing (using the reference list of a paper to identify additional papers; [21] on review articles retrieved with our search strategy. The search was updated in March 2022 revisiting all the original databases and grey literature sources. The records were exported into Endnote (version x9; Clarivate Analytics) and duplicates were removed [22]. Inclusion criteria were *1*) References published in English and *2*) after 1980. All records were screened in duplicate in Covidence (Veritas Health Innovation).

### Selection criteria

Search results were screened in duplicate. Any randomized controlled trial (RCT) or observational study was eligible for inclusion if it reported associations between HM components and infant anthropometrics. Data from RCTs were evaluated as observational studies because, in all cases, associations between HM composition and infant anthropometrics were secondary trial outcomes. We required that studies reported on healthy, term, HM-fed infants (aged 0–24 mo). Healthy was defined as term birth (37 wk, 0 d of gestation, or later) with no congenital or other morbidities and no admission in the neonatal intensive care unit, as described by study authors. Studies that included preterm infants were excluded unless it was possible to extract data for the term infants separately. Although breastfeeding exclusivity was not an inclusion criterion, it was recorded when reported by authors. Our main outcomes were weight-for-age Z-score (WAZ), length-for-age Z-score (LAZ), weight-for-length Z-score (WLZ), BMI or BMI-for-age Z-score, and growth velocity. Reference populations used to calculate Z-scores varied across studies, and some studies reported percentiles rather than Z-scores. To simplify the synthesis of results only Z-scores were summarized in heatmaps.

We also included articles that reported other infant anthropometrics, including but not limited to weight, length, rapid weight gain (as reported by study authors), total adiposity (percent body fat by DEXA [dual-energy x-ray absorptiometry] or skinfold thickness), body composition (fat mass, fat-free mass, percent fat mass by bioelectrical impedance spectroscopy or skinfold thickness), stunting, wasting, under- or overweight, and head circumference.

### Quality assessment

Articles were assessed for quality using a modified Newcastle–Ottawa scale [23] (Supplemental Table 1). On the basis of previous research [24], and in collaboration with multiple subject matter experts, we created a 17-point evaluation scale. We designated 8 points for HM exposure assessment, including HM collection and handling protocol (3 points), HM sample preparation (1 point), analytical method used to measure HM analyte (2 points), longitudinal HM sampling strategy (1 point), and accounting for infants’ HM intake (1 point); 5 points for confounders considered, including infant diet (2 points), birth anthropometrics (1 point), baseline characteristics of mothers and infants (2 points); and 4 points for infant anthropometry outcome assessment, including whether infant anthropometrics were measured by trained staff (1 point), using technical replicates (1 point), longitudinally (1 point), and with all infants measured within 1 wk of each other at each time point (1 point). Quality assessment for each article was conducted in duplicate by independent reviewers, with conflicts addressed through consensus. Overall quality scores between >13 and 17 were considered high; 7 and 13 moderate; and <7 low. Quality scores were also evaluated individually for exposure assessment (high: >6–8, moderate: 3–6, and low: <3), confounders considered (high: >4–5, moderate: 3–4; and low: <3), and outcome assessment (high: >3–4, moderate: 2–3, and low: <2).

### Data extraction

Data extraction was conducted using a standardized form that was developed and piloted in collaboration with subject matter experts. Data extracted included publication year, location, design, baseline characteristics of mothers and infants, HM sampling times, and timing of infant anthropometric measurements, whether HM values were reported as concentrations or estimated intakes, outcomes, associations reported (correlations and unadjusted and adjusted ß-estimates, as reported by study authors), and major confounders considered (via study design or statistical analyses) including maternal age, parity, maternal BMI, ethnicity, time postpartum, breastfeeding exclusivity (as stated by study authors—usually at time of enrollment), birth anthropometrics, infant age and sex, and any others reported. Study authors were contacted to request data in instances they were missing or presented in non-extractable formats. Each article was extracted in duplicate, and conflicts were addressed through consensus.

### Analytical strategies

Data were summarized in tables as reported by study authors, and directional associations reported for HM concentrations were visualized in heat maps with colors determined by vote counting based on the mean direction of significant associations [25]. Because of minimal studies reporting calculated daily intakes (CDIs), these were summarized narratively. Only studies reporting HM concentrations were reflected in the heatmaps. The color gradient was determined by *1*) assigning a score to each outcome (+1 for positive associations, 0 for no/assumed no association, and −1 for inverse associations) and *2*) taking the mean direction of association (range: −1 to +1) for all articles reviewed. Some papers only reported outcomes that were statistically significant; for these studies, we considered the unreported associations as “assumed no association.” Components investigated in just 1 study were excluded from heatmaps. When studies reported results from both HM concentration and estimated daily intake, only associations using concentrations were reported in heatmaps.

Narrative synthesis was conducted according to the synthesis without meta-analyses (SWiM) reporting guidelines [25]. Bioactives were divided into 3 categories of hormones, HMOs, and immunomodulatory components, based on their distinct roles within infant physiology. Bioactives that did not fall into these 3 categories were discussed individually.

## Results

### Description of included studies

**FIGURE 1:**
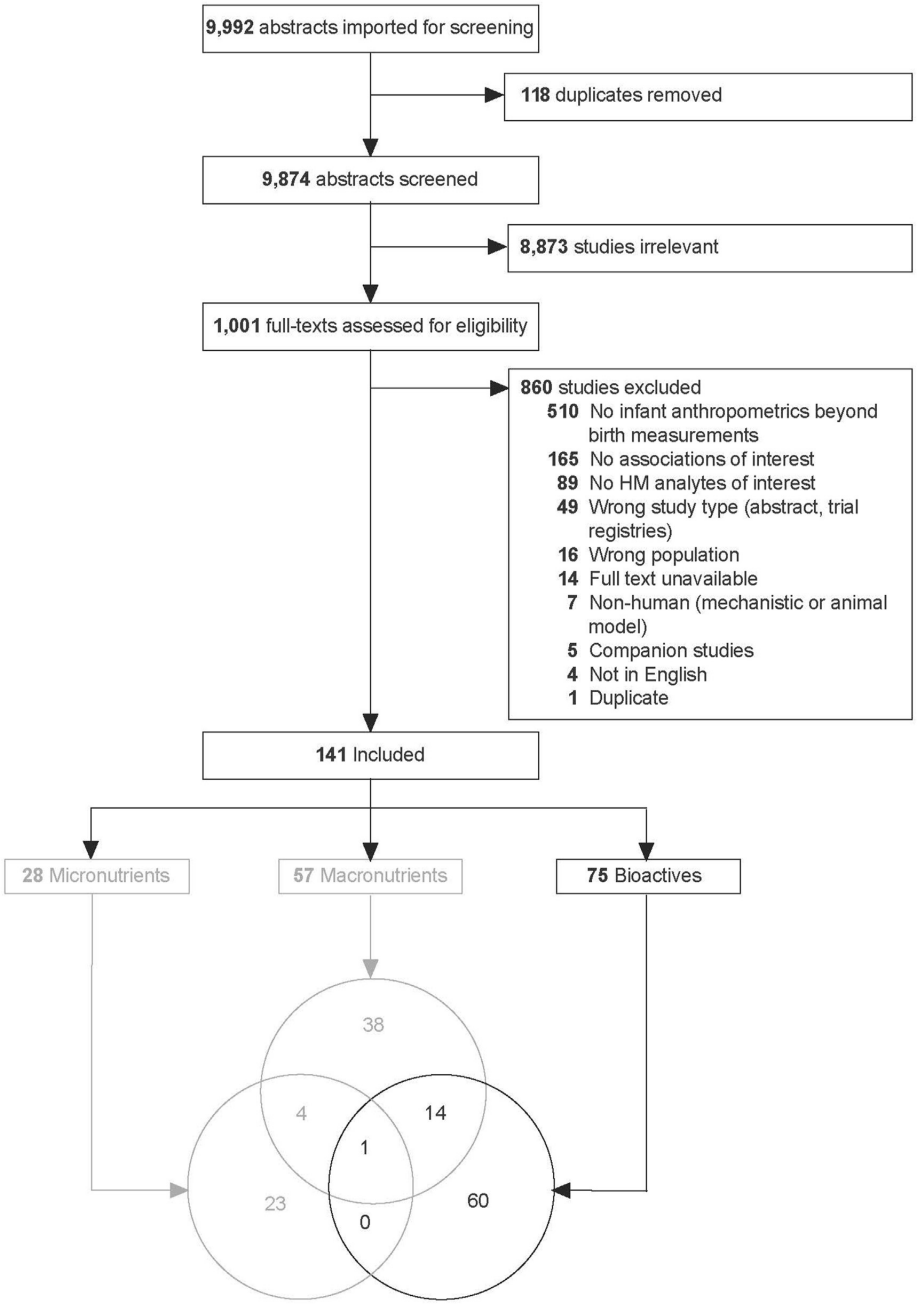
Systematic review of associations between HM bioactives and infant growth in the first 2 y: PRISMA flow diagram. Reasons for study exclusion were recorded in the order listed in the figure. Though some studies had more than 1 reason for exclusion, each study was only counted once (for example, if a study reported no HM analytes of interest and was not in English, it was recorded as the former). Bioactive studies are reported in the current paper; Macronutrient and Micronutrient studies are reported separately. HM, human milk.

In total, 9992 unique abstracts were identified and 1001 full texts were screened (Figure 1). The main reasons for excluding articles were no infant anthropometrics or only birth anthropometrics were reported (*n* = 510); no associations between HM analytes and infant anthropometrics were reported (*n* = 165); or no HM analytes of interest were reported (*n* = 89). Together, these 3 reasons accounted for 76% (764/1001) of the articles excluded during full-text screening. Overall, 141 articles were included for the broader systematic review, of which 75 articles examining bioactives in HM were included in this review.

Among the 75 included bioactive articles (Table 1) [[26], [27], [28], [29], [30], [31], [32], [33], [34], [35], [36], [37], [38], [39], [40], [41], [42], [43], [44], [45], [46]], none were published before 2000. The 75 articles represented 69 unique studies involving 9980 dyads. Thirty-two studies were conducted in low- and middle-income countries (LMICs, according to World Bank criteria) and 36 in high-income countries (HICs). One study examined milk components from 2 cohorts, 1 in the USA (HIC) and 1 in Mexico (LMIC [47]). Fifty-seven studies were longitudinal, including 5 RCTs and 1 case-control study. The remaining 12 studies were cross-sectional.

**TABLE 1:** Detailed characteristics and results of included studies reporting on human milk bioactives and infant anthropometrics (organized alphabetically by first author)

Authors, country, publication year (income setting)	Design and participants	Milk sampling time(s), analytes and units	Anthropometric outcome assessment time(s), measures and standards	Associations^2^	Major confounders considered
Aksan et al. Turkey, 2021 (UMIC) [26]	Cross-sectional 88	3 mo osteopontin (concentrations)	Birth, 3 mo weight, height, HC (WHO standards)	(+) Association for HM osteopontin and weight at 3 mo (+) Association for HM osteopontin and length at 3 mo (+) Association for HM osteopontin and HC at 3 mo	None

Alderete et al. USA, 2015 (HIC) [48]	Longitudinal 37 (25 analyzed)	1 mo, 6 mo HMOs (see Supplemental Table 4) (concentrations)	1 mo, 6 mo weight, length, % fat, total fat, lean mass, trunk fat mass	1 mo: (−) Association for LNFPI and weight (−) Association for HMO diversity and 1 mo fat mass and % body fat (−) Association for HMO evenness and 1 mo fat mass and % body fat 6 mo: (+) Association for DSLNT and length (−) Association for LNFPI and weight, lean mass, and fat mass (+) Association for DSLNT and fat mass (+) Association for LNFPII and fat mass (−) Association for LNnT and body fat (+) Association for FDSLNH and body fat (−) Association for 1 mo LNFPII and 6 mo fat mass Specific HMOs accounting for increase in % of observed variance in body composition: 1 mo - including LNFPI explained 18% more of variance in weight; HMO diversity and HMO evenness explained 20% more of variance in % fat and 17% more of variance in fat mass Specific HMOs accounting for significant increase in % of observe red variance in body composition: 6 mo - including DSLNT explained 7% more of variation in length; including LNFPI explained 6% more of variance in weight and 13% more of variation in lean mass; including LNFPI, DSLNT, AND FDSLNH explained 33% more of variation in fat mass; including LNnT explained 23% more of variance in percent fat	Maternal pre-pregnancy BMI, pregnancy weight gain, infant sex, age

Alsharnoubi et al. Egypt, 2019 (LMIC) [49]	Cross-sectional 84	71.5 ± 61.4 d TGF-beta 1 (concentrations)	71.5 ± 61.4 d weight, length, HC, postnatal weight gain, TSF	(+) Association for TGF- β 1 and average weight infants. (No) Association between TGF- β 1 and below-average weight infants. (No) Association between TGF- β 1 and infant length	Maternal age, specific food, contraceptive use; infant’s age, weight, length

Anderson et al.Philippines, 2016 (LMIC) [50]	Cohort 132 (117 analyzed)	10 d–4 y (only mothers nursing s <2 y included) adiponectin (concentrations)	10 d–4 y (only s <2 y included) WAZ, BMIZ	(+) Association for milk adiponectin and WAZ (+) Association for milk adiponectin and BMIZ	Maternal BMI, infant age, BF frequency

Andreas et al.England, 2016 (HIC) [51]	Cohort 105	1 wk and 3 mo ghrelin, insulin, leptin, resistin (concentrations)	1 wk and 3 mo weight, length, HC, weight change from: birth to 7 d, 7 d–3 mo, birth to 3 mo	(No) Association for hormone concentrations in foremilk samples collected at 1 wk and anthros NOT PREDICTIVE: (−) Association for weight and hindmilk insulin at 1 wk (−) Association for length at 1 wk and foremilk insulin at 3 mo (No) Association for anthro and hormone concentrations in hindmilk samples collected at 3 mo	None reported

Baroncelli et al.Malawi, 2018 (LIC) [52]	Cohort 149	6 mo sCD14 (concentrations)	Unclear, assuming 6 mo weight gain	(No) Association for sCD14 and weight gain	None reported

Binia et al.Europe, 2021 (HIC) [53]	Cohort 375 (350 analyzed)	V1 = 2 (0–3) d, V2 = 17 ± 3 d, V3 = 30 ± 3 d, V4 = 60 ± 5 d, V5 = 90 ± 5 d, and V6 = 120 ± 5 d HMOs (see Supplemental Table 4) (concentrations)	V1 = 2 (0–3) d, V2 = 17 ± 3 d, V3 = 30 ± 3 d, V4 = 60 ± 5 d, V5 = 90 ± 5 d, and V6 = 120 ± 5 d) weight, length, HC, weight-for-length, Fat mass index, weight change rate, length change rate, HC change rate, weight-for-length change rate (WHO standards)	(−) Association for 3'SL and length (+) Association for MFLNH-III, LNFP III and HC (−) Association for A-Tetra and delta HC (−) Association for LNnT and delta length (+) Association for LSTc and weight for length (No) Association for any HMOs and FMI (or fat accretion)	Maternal postpartum BMI, infant sex, infant birth weight, and fat mass at V1

Bronsky et al.Czech Republic, 2011 (HIC) [54]	Cohort 72	Colostrum, 1 mo, 3 mo, 6 mo, 12 mo adiponectin, AFABP, leptin (concentrations)	Birth, 1 mo, 3 mo, 6 mo, 12 mo body weight, length	(−) Association for AFABP at mo 1 and body weight (−) Association for AFABP and body length at birth (−) Association for leptin and body length at birth (+) Association for weight gain during y 1 and adiponectin concentration at mo 6	None reported

Brunner et al.Germany, 2015 (HIC) [55]	RCT 208 (152 analyzed at 6 wk, 120 at 4 mo).	6 wk, 4 mo adiponectin, leptin (concentrations)	Birth, 6 wk, 4 mo, 1 y, 2 y weight, BMI, sum 4 SFT, body fat percentage, fat mass (g), lean body mass (g), weight gain (6 wk–4 mo)	(−) Association for leptin at 4 mo and concurrent weight and lean body mass (adjusted) (No) Association for leptin at 6 wk and any growth and body composition until 2 y (No) Association for leptin at 4 mo and growth and body composition and follow-up later than 4 mo (−) Association for adiponectin and lean body mass at 4 mo (+) Association for adiponectin (unadjusted) and weight gain and fat mass ≤2 y (adjusted – ≤1 y)	Maternal pre-pregnancy BMI, gestational weight gain, pregnancy duration, infant sex, infant ponderal index at birth, mode of infant feeding at 4 mo

Bruun et al.Denmark, 2018 (HIC) [56]	Cohort 100	17.1 ± 3 wk OEA, SEA, PEA (concentrations)	4 mo abdominal circumference, weight, length, triceps & subscapular skinfold thickness, WAZ, HAZ, WHZ, BMIZ, delta weight since birth, delta weight since birth per day, delta WAZ since birth	(−) Association for SEA concentration and triceps skinfold thickness (−) Association for SEA concentration and weight gain per day since birth	Pre-pregnancy BMI, infant birth weight, early infant formula supplementation

Campbell et al.The Gambia, 2006 (LIC) [27]	RCT 65 (48 analyzed)	CagA, VacA (concentrations)	Monthly from 4 wk–44 wk (samples pooled from wk 4 onwards) WLZ	(+) Association for weight gain and the presence of maternal milk VacA antibodies (−) Association for VacA-specific IgA antibodies in maternal milk and reduction in growth in Gambian children colonized with H. pylori	None reported

Cannon et al.Australia, 2015 (HIC) [57]	Cohort 19	Assumed 3 wk–21 wk leptin (concentrations)	Assumed 3 wk–21 wk weight	^1^Primary relationship reported was not for anthros and milk component (No) Association for [leptin] or total daily leptin intake and weight	None reported

Cesur et al.Turkey, 2012 (UMIC) [58]	Longitudinal 25 (19 analyzed)	1 mo, 4 mo ghrelin, adiponectin (concentrations)	1 mo, 4 mo weight, weight gain, BMI	(+) Association for level of 4th mo HM Active Ghrelin concentrations and weight gain of during study period (No) Association for adiponectin levels in HM and growth parameters of s	None reported

Chan et al.Canada, 2018 (HIC) [59]	Cohort 420	3 mo–4 mo adiponectin, leptin, insulin (concentrations)	4 mo, 1 y weight, length (WHO standards)	(−) Association for HM leptin, insulin and WFL, BMIZ at 4 mo (No) Association for milk adiponectin and body composition ^1^these patterns of association persisted to 1 y	Pre-pregnancy maternal BMI, total BF duration, ethnicity, parity, diabetes, smoking, BF exclusivity, lactation stage

Cheema et al. Australia, 2021 (HIC) [60]	Cohort 67 (57 analyzed)	2 mo insulin, glucose, leptin (concentrations and intakes)	3 mo weight, length, BMI, HC, FFM, FFMI, Fat Mass, Fat Mass Index, % Fat Mass, Fat Mass/Fat-Free Mass (ratio) and Z-scores (WHO standards)	(No) Association for insulin and anthropometrics	Infant birth weight, infant sex, gestational age, 24-h milk intake

Cheema et al. Australia, 2022 (HIC) [61]	Cohort 67 (60 analyzed)	2 mo HMOs (see Supplemental Table 4) (concentrations and intakes)	3 mo weight, length, BMI, HC, FFM, FFMI, Fat Mass, Fat Mass Index, % Fat Mass, Fat Mass/Fat-Free Mass (ratio) and Z-scores (WHO standards)	HM Concentrations Nonsecretors: (+) Association for [DFLNT] and weight, height, WFAZ, LFAZ and Fat mass (−) Association for [FLNH] and length, LFAZ Secretors: (+) Association for [3'SL] and FFM Overall (not stratified for NS and Secretors): (−) Association for [FLNH] and weight (+) Association for log[DFLNH] and weight, length, LFAZ, FFM, (−) Association for log[LNnT] and length, LFAZ (−) Association for log[LNFP III] and Fat mass (%) and Fat mass to fat-free mass ratio HM Daily Intakes Nonsecretors: (+) Association for 6'SL and weight, WFAZ, Fat mass and FMI (−) Association for logFDSLNH and BMI, WFAZ, BMI for Age Z, logFFMI Secretors: (+) Association for log3'SL and weight, length, WFAZ, logFFM, logFFMI Overall (not stratified for NS and Secretors): (+) Association for 2'FL and weight and Fat mass, (+) Association for 3'FL and weight, length, WFAZ, LFAz, logFFM, logFFMI (+) Association for log(DFLaz) and weight, BMI, BMIZ, logFFM, logFFMI (+) Association for log(DFLNH) and weight, length, WFAZ, LFAZ, logFFM (+) Association for log(LSTb) and BMI (+) Association for DFLNT and BMI, BMIZ, and logFFMI. (+) Association for log(DFLNH) and weight, length, LFAZ, FFM, (−) Association for log[LNnT] and length, LFAZ (−) Association for log[LNFP III] and Fat mass (%) and Fat mass to fat-free mass ratio"	Infant birth weight, infant sex, gestational age, and 24-h milk intake, infant body composition, maternal body composition, maternal weight, FFM, Fat Mass and Fat Mass Index

Davis et al.The Gambia, 2017 (LIC) [62]	RCT 33	4 wk, 16 wk, 20 wk HMOs (see Supplemental Table 4) (concentrations)	4 wk, 16 wk, 20 wk WAZ, HAZ (Gambian reference)	(+) Association for 3'SL and WAZ at 20 wk (−) Association for LSTc and WAZ at 20 wk (+) Association for DFLNHa and HAZ at 20 wk (+) Association for LNFP I + III and HAZ at 20 wk	None reported

Doneray et al.Turkey , 2009. (UMIC) [28]	Cohort 15	1 d, 21 d–30 d leptin (concentrations)	1 d , 21 d–30 d weight, height, BMI, delta BMI	No reported association for anthropometrics and HM leptin. (−) Association for delta BMI and leptin	None reported

Dundar et al.Turkey, 2005 (UMIC) [29]	Longitudinal 47	15 d; 1 mo, 2 mo, 3 mo leptin (concentrations)	15 d; 1 mo, 2 mo, 3 mo birth weight, weight gain during first 15 d and first mo	(+) Association for birth weight and leptin at 15 d (−) Association for weight gain during first 15 d and leptin at 15 d (−) Association for weight gain during first mo and leptin at 15 d	None reported

Durilova et al.Czech Republic, 2010 (HIC) [63]	Cross-sectional 20	2 wk–27 wk (EC group), 12 wk (control) IL-4, IL-6, IL-10, IL-17, IL-18, IL-23, interferon-gamma (IFN-gamma) and transforming growth factor beta 1 (TGF- β1) (concentrations)	Unclear body weight	(+) Association for IL-4 and body weight (−) Association for IL-6 and body weight	None reported

Ellsworth et al.USA, 2020 (HIC) [64]	Longitudinal 55 (32 analyzed)	2 wk (average 16 d) insulin (concentrations)	2 wk, 2 mo, 6 mo WAZ change, WLZ change, BMIZ change, LAZ change, HCAZ change (WHO standards)	(+) Association for milk insulin and WFA from 2 wk to 6 mo and HCAZ change from 2 wk to 2 mo in infants receiving any type of nutrition	Infant sex

Enstad et al.USA, 2021 (HIC) [65]	Longitudinal 40	1 mo, 4 mo leptin, IL-8, IL-6, IL-1beta, MDA (concentrations)	V1, V2, V3, V4, V5, V6, V7 (that is 1 mo, 2 mo, 3 mo, 4 mo, 5 mo, 6 mo, 7 mo) WAZ, LAZ, BMIZ, % fat mass, % lean mass, growth trajectory from mo 1 to 7 mo	(+) Association for IL-1beta and LAZ in infants at V4 (+) Association for IL-8 and BMIZ at V7 (+) Association for IL-1beta and BMIZ at V7 (+) Association for leptin and lean mass at V4 (−) Association for leptin and % fat mass at V4 (−) Association for leptin and BMIZ at V1 (+) Association for leptin and % lean mass at V1 and V4 (+) Association for cytokines and WAZ at birth (−) Association for cytokines and WAZ for 1 mo–2 mo (+) Association for cytokines and WAZ after 2 mo	Race, infant age at time of growth measurement, baseline infant measurement (measured at birth or mo 1), sex

Fatima et al.Pakistan, 2019 (LMIC) [66] Fatima et al. Pakistan, 2022 (LMIC) [67]	Case Control 66	72 h , 6 wk irisin, SREBP-1c (concentrations)	Newborn weight, 6 wk weight	(+) Association for irisin and infant weight at 6 wk (+) Association for mature HM irisin and infant weight at 6 wk ^1^association is lost when adjusted for maternal BMI (+) Association for HM chemerin and weight at 6 wk	Maternal BMI, stratified by GDM status

Fields et al.USA, 2017 (HIC) [68]	Longitudinal 37 (30 analyzed)	1 mo and 6 mo insulin, leptin, IL-6, TNF-alpha (concentrations and intakes)	1 mo, 6 mo weight, length, % fat, total fat mass, total FFM, trunk fat mass	(−) Association for mo 1 leptin levels and mo 6 body length, % fat, total fat mass, and trunk fat mass (No) Association for mo 1 leptin levels and total fat-free mass (No) Association for mo 1 insulin levels and mo 6 body length, % fat, total fat mass, total fat-free mass and trunk fat mass (No) Association for mo 1 TNF levels and mo 6 body length, % fat, total fat mass, total fat-free mass and trunk fat mass (No) Association for mo 1 IL6 levels and mo 6 body length, % fat, total fat mass, total fat-free mass and trunk fat mass	Infant sex, pregravid maternal BMI category, stage of lactation (1 mo vs. 6 mo)

Galante et al.Finland, 2020 (HIC) [69]	Cohort 501	2.6 ± 0.4 mo leptin, adiponectin, IGF-1, cGP (concentrations)	1 y, 2 y, 3 y, 5 y weight, weight gain, BMIZ (Finnish reference)	(−) Association for IGF-1 and weight gain from birth to 2 y (+) Association for IGF-1 and weight Z at 13 mo (−) Association for IGF-1 and weight Z at 3 and 5 y (−) Association for cGP and weight Z at 13 mo (+) Association for IGF-1:cGP ration and weight Z at 13 mo (−) Association for IGF-1:cGP ratio and BMIZ at 3 and 5 y (+) Association for cGP and BMIZ at 5 y	Maternal pre-pregnancy BMI, infant sex, BF duration, intro of solid foods, infant birthweight

Goran et al.USA, 2017 (HIC) [30]	Longitudinal 37	1 mo and 6 mo insulin (concentrations and intakes)	1 mo and 6 mo weight, WLZ, lean mass, fat mass, adiposity	(No) Association for BM Insulin and any anthropometrics	Maternal pre-pregnancy BMI, infant sex, infant weight at 1 mo

Gridneva et al.Australia, 2020 (HIC) [31] Gridneva et al. Australia. 2018 (HIC) [70] Gridneva et al. Australia. 2021 (HIC) [71]	Cohort 20	2 mo, 5 mo, 9 mo, 12 mo lactoferrin, lysozyme, sIgA (concentrations and intakes)	2 mo, 5 mo, 9 mo, 12 mo fat mass (US4SF, BIS, US2SF), fat mass index (US4SF, BIS, US2SF), delta weight, delta BMI	(+) Association for lysozyme CDI at 12 mo and decrease in FFMI for 5 and 12 mo (−) Association for lactoferrin CDI and FFMI (ultrasound) at 12 mo (+) Association for lysozyme CDI and Fat mass (ultrasound) and FMI (ultrasound) at 12 mo (−) Association for CDI adiponectin and lean body mass. (+) Association for CDI adiponectin and adiposity (+) Association for CDI skim milk leptin and adiposity (No) Association for HM adiponectin and weight gain. Higher CDI of skim milk leptin was associated with a lower accrual of FFM over 12 mo (−) Association for lactoferrin concentration and visceral depth (+) Association for LActose, total carbohydrates and total protein (intakes) and subcutaneous abdominal fat area (no other significant associations)	Fixed effect for infant age and age Interaction with milk component

Guler et al.Turkey, 2021 (UMIC) [72]	Cohort 40	2 mo (60 d, SD=10) leptine, ghrelin, adiponectin, IGF-1 (estimated daily intake & concentrations)	2 mo (60 d, SD=10) weight, length, HC, WLZ (WHO standards)	(No) Association for Leptin and WLZ at 2 mo (No) Association for ghrelin and WLZ at 2 mo (No) Association for adiponectin and WLZ at 2 mo (No) Association for IGF-1 and WLZ at 2 mo	Maternal BMI, age, parity, gestational weight gain and sex

Hollanders et al.Netherlands, 2019 (HIC) [73]	Longitudinal 42	30 (±5 d) cortisol, cortisone (concentrations)	1 mo, 2 mo, 3 mo length, weight, BMI, FMI, FFMI, % fat	(No) Association for glucocorticoid rhythmicity at 1 mo and body composition or growth at 3 mo	HADS-Score, maternal pre-pregnancy BMI, ethnicity, socioeconomic status, gestational weight gain, parity, mode of delivery, mode of HM at 3 mo of age (that is, < or > 80% HM), infant sex, birth weight, gestational age

Isganaitis et al.USA, 2019 (HIC) [74]	Longitudinal 37 (31 analyzed at 1 mo, 26 at 6 mo)	1 mo, 6 mo various metabolites (concentrations)	1 mo, 6 mo weight, length, % body fat (that is, fat mass %), total fat mass, total lean mass, trunk fat mass, fat accrual (that is, difference in fat mass from 1 mo to 6 mo)	Milk metabolites correlating with weight status at 1 mo : (+) Association for 1-linoleoyl GPE, 2-palmitoyl-GPE, 3-methylxanthine, myo-inositol, pseudouridine, theobromine and weight (−) Association for 1-palmitoylplasmenylethanolamine, 1-stearoyl GPE, 6-sialyl-N-acetllactosamine, acetoacetate, DHA 22:6n:3, guanosine, hexanoylcarnitine C6, nicotinamide, phenylacetylglutamine and weight. Milk metabolites correlating with % body fat at 1 mo : (+) Association for 2-aminobutyrate, 3-methylxanthine, carnitine, cytidine, pseudouridine, theobromine, and % body fat. (−) Association for 1-palmitoylplamenylethanolamine, hexanoylcarnitine C6, X-11616, X-15558, X-15562 and % body fat Milk metabolites correlating with fat mass percentat 1 mo , adjusted for sex, gestational age, parity, and birthweight: (+) Association for 2-aminobutyrate and fat mass percentage. (−) Association for nicotinamide riboside, X-11616 and fat mass percentage. Milk metabolites correlating with fat mass percentat 6 mo , adjusted for parity and sex: (+) Association for biliverdin, mannose, X-11684, X-11687, X-12565 and fat mass (−) Association for 1-stearoyl-GPI 18:0, 2-linoleoyl-GPC 18:2, 2-oleoyl-GPE 18:1, 5-dodecenoate 12:1n7, 7-methylurate, AMP, creatinine, eicosapentaenoate EPA 20:5n3, linolenate 18:3n3 or n6, orotate, stearidonate, X-08893, X-12216, X-15503 and fat mass percentage. Milk metabolites correlating with fat mass percentat 6 mo , adjusted for parity, sex, and birthweight: (+) Association for biliverdin, X-11684, X-12565 and fat mass percentage (−) Association for 1-oleoyl-GPE 18:1, 1-palmitoyl GPC 16:0, 1-stearoyl-GPI 18:0, 2-linoleoyl-GPC 18:2, 2-oleoyl-GPE 18:1, 7-methylurate, adenosine, AMP, carnitine, cholesterol, creatinine, DPA 22:5n5, DPA 22:5n6, eicosapentaenoate EPA 20:5n3, NAD, orotate, palmitoyl sphinghomyelin, stearidonate, X-08893, X-12216, X-15503 and fat mass percentage. Milk metabolites at 1 mo correlating with fat accrual, adjusted for sex, gestational age, and parity: (+) Association for 1-palmitoyl-GPE 16:0, adenine, caffeine, citrate, gluconate, ornithine, urate and fat accrual (−) Association for 3-indoxyl sulfate, 7-methylurate, X-16101 and fat accrual. Milk metabolites at 1 mo correlating with fat accrual, adjusted for sex, gestational age, parity, and birthweight: (+) Association for 1-palmitoyl-GPE 16:0, adenine, gluconate, ornithine and fat accrual (−) Association for 3-indoxyl sulfate, 7-methylurate, beta-alanine, carnitine and fat accrual	For weight status at 1 mo: gestational age, parity, infant sex For % body fat at 1 mo: gestational age, parity, infant sex For fat mass % at 1 mo: gestational age, parity, infant sex, infant birthweight For fat mass % at 6 mo: parity, infant sex, infant birthweight For fat accrual: gestational age, parity, infant sex, infant birthweight

Jiang et al.China, 2021 (UMIC) [75]	Cross-sectional 150 (143 analyzed) randomly selected from 1800 in the Chinese Human Milk Project	15 d–180 d proteome, lipidome, and glycome (concentrations)	15 d–180 d weight, length, LAZ, WAZ, BMIZ, and WFLZ (WHO standards)	Factor 1: (high in 128 proteins related to platelet degranulation, endopeptidase activity, signal transduction, immune response and low in ***β***-casein; high in Phospholipids including PE, PC, Ceramide, SM, TG-SU2, TG-UUU and low in TG-SSS; high in LNnH, LNDFH II and low in 3’SL) (−) Association for Factor 1 and LAZ Factor 2: (high in Vitronectin, CD81 molecule, complement C4A, fibroblast growth factor binding protein 1, milk fat globule-EGF Factor 8 protein, immunoglobulin heavy constant gamma 4 and low in as1-casein; high in TG-SSS and low in Phospholipids including PE, PC, SM; high in LNnH, LSTa, LSTb, LSTc, 3’FL, 2’FL and low in DSLNT) (−) Association for Factor 2 and BAZ and WAZ Factor 3: (high in no proteins and low in 27 proteins related to platelet degranulation, endopeptidase activity, innate immune response; high in Phospholipids including PE, PC, PI, LPC, LPE, LPI, SM, Ceramide and low in TG-S2U; high in LSTc, LNFP II and low in 6’Sl and 2’FL) (+) Association for Factor 3 and LAZ.	Infant age, infant sex, birth weight, birth length, maternal age and city

Jorgensen et al.Malawi, 2020 (LIC) [76]	Longitudinal 659 samples collected (647 analyzed for HMOs, 637 for protein)	6 mo untargeted HMOs (concentrations)	6 mo, 12 mo change in LAZ, WAZ, WLZ and HCZ from 6 mo–12 mo	For secretors + nonsecretors combined: In primary analyses: (No) Association for abundance of groups of HMOs or concentrations of IgA, lactalbumin, or lactoferrin and growth indicators. In exploratory analyses: (+) Association for unnamed HMO 5311a and delta LAZ and WAZ. (−) Association for unnamed HMO 5330a and delta HCZ. (+) Association for unnamed HMO 5230b and delta WAZ and WLZ. (+) Association for unnamed HMO 4320a and delta LAZ. (+) Association for unnamed HMO 6400a and delta WAZ. (+) For unnamed HMO 6400b and WAZ. For secretors only: In primary analyses: (+) Association for absolute abundance of all HMOs and delta LAZ. In exploratory analyses: (−) Association for LNT + LNnT and delta WLZ. (−) Association for LNT and delta WAZ, WLZ, HCZ. (−) Association for LNFP I + III and WLZ. (−) Association for LDFT and delta LAZ. (+) Association for LDFT and changein WLZ. (+) Association for 3'SL and delta HCZ. (+) Association for 5230a + DFLNnO I/DFLNO II and delta WAZ. (−) Association for IFLNH-I and delta WLZ. (+) Association for LSTa and delta LAZ. (−) Association for LSTa and delta WLZ (+) Association for DFLNHc and delta LAZ. (−) Association for DFLNHc and delta WLZ. (−) Association for 6'SL and delta HCZ. (+) Association for unnamed HMO 5130b and delta LAZ. (+) Association for unnamed HMO 4240a and delta LAZ. For nonsecretors only: In primary analyses: (No) Significant associations for bioactive proteins or groups of HMOs and growth. In exploratory analyses: (−) Association for LNFP II and delta WLZ. (−) Association for unnamed HMO 4120a and delta WLZ	Secretor status, baseline age, BMI, parity, education, food security, HIV status, Hb, household assets, residential location, season at time of sample collection, intervention group, infant sex

Khaghani et al.Iran, 2006 (LMIC) [32]	Longitudinal 244	1 mo, 2 mo, 3 mo, 4 mo, 5 mo, 6 mo leptin (concentrations)	1 mo, 2 mo, 3 mo, 4 mo, 5 mo, 6 mo weight, height, HC	(No) Association for leptin and height, weight, and HC	None reported

Khodabakhshi et al.Iran, 2015 (LMIC) [77]	Cross-sectional 80	Unclear. ghrelin, adiponectin, leptin, EGF, IGF-1 (concentrations)	2 mo, 4 mo, 6 mo weight, height	(−) Association for ghrelin, EGF-1 and weight status (normal weight infants' mothers' milk had higher concentrations of both ghrelin and EGF1) (−) Association for adiponectin and 2nd mo weight for normal weight infants	None reported
Kon et al.Russia, 2014 (HIC) [78]	Longitudinal 103 (99 analyzed)	1 mo, 2 mo, 3 mo IGF-1, ghrelin, leptin, adiponectin (concentrations)	Weight gain	(+) Association for high weight gain status and IGF-1 at all lactation ages (+) Association for high weight gain status and leptin at 2 mo and 3 mo (+) Association for high weight gain status and ghrelin at 1 mo and 2 mo (No) Association for weight gain status and adiponectin (No) Association for weight gain status and levels of IGF-1 in HM at all lactation ages	None reported

Kuziez et al.Philippines, 2020 (LMIC) [79]	Cross-sectional 126 (69 analyzed)	9 d–24 mo EGF (concentrations)	9 d–24 mo length, weight, HC, mid-upper arm circumference, 7 skinfold thicknesses	(No) Association for human EGF and length, weight, HC, mid-upper arm circumference, 7 skinfold thicknesses	Maternal birth weight, maternal gestation age, infant age, maternal energy intake.

Lagstrom et al.Finland, 2020 (HIC) [80]	Longitudinal 1797 (802 analyzed)	3 mo HMOs (see Supplemental Table 4) (concentrations)	3 mo, 6 mo, 8 mo, 1 y, 2 y, 3 y, 4 y, 5 y WAZ, LAZ	In secretors: (−) Association for HMO diversity and LAZ and WAZ during first y (−) Association for HMO diversity and LAZ at 1 y and 5 y (+) Association for 2'FL and LAZ for 3 mo and 12 mo, and 1 y and 5 y (+) Association for 2'FL and WAZ for 3 mo and 12 mo (−) Association for LNnT and weight and LAZ throughout first 5 y (+) Association for HMO-bound fucose and LAZ and WAZ for 3 mo and 12 mo, and 1 y –5 y (−) Association for LSTb and LAZ for 3 mo and 12 mo (+) Association for 3’FL and WAZ from 3 mo to 13 mo and 1 y –5 y (+) Association for 3'SL and WAZ from 3-12 mo and 1-5 y (+) Association for DFLac and WAZ from 3-12 mo (−) Association for LSTb and WAZ from 3-12 mo For non-secretor mothers: (No) Association for HMO diversity and LAZ	Maternal secretor status, maternal pre-pregnancy BMI, infant sex, birthweight z-score, time point (that is, 3 mo mo–12 mo or 1 y –5 y)

Larson-Meyer et al.USA, 2020 (HIC) [81]	Cohort 24	1 mo, 6 mo leptin, PYY, GLP-1, ghrelin (concentrations)	1 mo, 6 mo, 12 mo WAZ, weight gain (WHO standards)	(−) Association for average milk GLP-1 and WAZ at 6 mo (−) Association for average milk leptin at 1 mo and WAZ at 12 mo	None reported

Larsson et al.Denmark, 2019 (HIC) [82]	Longitudinal 30	5 mo–6.5 mo, 9 mo HMOs (see Supplemental Table 2) (Concentrations and intakes. Associations determined for concentrations)	Birth, 5 mo, 9 mo WAZ, BAZ, HAZ, FMI, FFMI, weight velocity	Analysis of Secretors only: (+) Association for 2'FL and 0-5 mo weight velocity (+) Association for 2'FL and FMI at 5 mo (+) Association for DFlac and weight velocity and length at 5 mo (+) Association for 3'SL and length at 5 mo (−) Association for 6'SL and BAZ at 5 mo (−) Association for LNnT and length, weight velocity, FMI, and delta WAZ from birth to 5 mo (+) Association for total HMO-bound fucose and weight velocity 0 mo–5 mo and FMI (+) Association for total HMO and weight velocity from 0 mo to 5 mo and FMI at 5 mo (−) Association for HMO diversity and weight velocity and FMI at 5 mo Analysis of secretors + nonsecretors combined: (−) Association for LNnT and length (−) Association for HMO diversity and BAZ, weight velocity, and FMI at 5 mo	Maternal secretor status, infant sex

Larsson et al.Denmark, 2018 (HIC) [83]	Longitudinal 59 (30 analyzed)	First visit: 5 mo–6.5 mo old; Second visit: 9 mo (±2 wk); Third visit for HW-group only: 18 mo ± 4 wk adiponectin, leptin, lysozyme, sIgA, lactoferrin (concentrations and intakes, associations for concentrations)	First visit: 5 mo–6.5 mo old; Second visit: 9 mo (±2 wk); Third visit for HW-group only: 18 mo ± 4 wk weight, recumbent length, mid-upper-arm circumference, HC, lower leg circumference, recumbent waist and thorax circumference, triceps and subscapular skinfold thickness, WAZ, LAZ, BAZ, triceps skinfold for z-score, subscapular skinfold-for-z-score (SSFZ)	(No) Association for milk concentrations of adiponectin, leptin, lysozyme, sIgA, lactoferrin and 's anthropometry or delta Z-scores from birth to the 5-mo visit	Maternal fasting time, infant sex

Leghi et al.Australia, 2021 (HIC) [84]	Open label crossover 18	Week 1 (baseline), wk 2 wk and wk 3 of intervention (Infant age not standardized) leptin, insulin, adiponectin, fat, protein, lactose (concentrations and intakes)	Week 1 (baseline), wk 2 and wk 3 of intervention (Infant not standardized) weight, length, HC, BMI, WFLZ, WFAZ, LFAZ, (WHO standards)	(NO) outcomes reported for Hormones (Assumed no relationship)	Maternal BMI

Logan et al. Germany, 2019 (HIC) [85]	2 Cohorts UBCS: 1042 SPATZ: 934	6 wk leptin (concentrations)	2 d BMI or change in BMIZ from birth to 2 y	(−) Association for 6 wk leptin and BMI at 4 wk–5 wk (+) Association for 6 wk leptin and greater increases in BMI after 4 wk–5 wk (No) Association for 6 mo leptin and growth	Maternal (age, birth country, parity, education, BMI, history of smoking), birth (gestational age at delivery, delivery mode), and other factors associated with BF or HM composition (BF duration, exclusivity, feedings per day, BF method (breast or pump), collection time of day, time from last feeding)

Liu et al.China, 2022 (UMIC) [86]	Longitudinal 110	4 wk, 8 wk, 12 wk HMOs (see Supplemental Table 4) (concentrations)	4 wk, 8 wk, 12 wk weight, length, HC	(+) Association for LDFT, 3'SL and body weight at T2 (8 wk). (−) Association for DSLNT (4 wk) and LNT (4 wk, 8 wk and 12 wk) and body weight (+) Association for total fucosylated HMOs (4 wk), total sialylated HMOs (8 wk), 3'SL (8, 12 wk) and BMI (−) Association for LNT (12 wk) and BMI (−) Association for 3'SL (4 wk), LNT (8 wk), LNFP-1 (12 wk) and DSLNT (4 wk, 8 wk , 12 wk) and HC	No infant birth data included in analysis

Menzel et al.Germany, 2021 (HIC) [87]	Cohort 153 (145 analyzed)	3 mo HMOs (see Supplemental Table 4) (concentrations)	3 mo, 6 mo, 1 y, 2 y length, weight, HC, BMI (reported as SDs), growth velocity between 3 mo and 1 y	Nonsecretors: (−) Association for height and LNT at 2 y (No) Association for height and 2’FL, 3’-FL, 3′SL, 6′SL, LNnFP, or LNFP-V. (−) Association for growth velocity and LNnT at 3 mo–1 y and 1 y –2 y (−) Association for BMI-SDS and LNFP-V at 3 mo, 6 mo, 1 y and 2 y. (−) Association for BMI-SDS and 6'SL at 3 mo, and 1 y (−) Association for LNFP-V and HC at 3 mo, 1 y and 2 y. (+) Association for LNnFP and HC at 2 y Secretors: (+) Association for height and LNFP I at 3 mo, 6 mo and 12 mo (No) Association for height and 2’FL, 3‘FL, 3′SL, 6′SL, LNnFP, or LNFP-V. (−) Association for growth velocity and LNFP I at 1 y –2 y (No) Association for growth velocity and 2’FL, (−) Association for BMI-SDS and LNT and LNFP-V at 2 y (+) Associations for BMI-SDS and LNnFP at 2 y. (−) Association for BMI-SDS and 2'FL at 3m. (No) Association for BMI-SDS and LNFP I (+) Association for LNFP I and HC at 6m (No) Association for HC and 2’FL, Overall (not stratified for NS and Secretors): (No) Association for height and 2’FL, 3-FL, 3′SL, 6′SL, LNnFP, or LNFP-V. (−) Association for growth velocity and LNT and LNFP-V at 3 mo–1 y (No) Association for growth velocity and 3-FL, 6′SL and LNnFP (No) Association for BMI-SDS and 3-FL and LNnT (No) Association for HC and 3-FL, 3′SL, 6′SL or LNT"	Secretor status, maternal pre-pregnancy weight and height and infant birth parameters

Mesripour et al.Iran, 2002 (LMIC) [33]	Longitudinal 23 (19 analyzed)	1 mo, 6 mo FSH, LH, estradiol, progesterone (concentrations)	1 mo, 6 mo height, weight, HC	(No) Association for hormones and growth indices after first mo (−) Association for FSH, LH, progesterone and weight after 6 mo	None reported

Miralles et al.Spain, 2006 (HIC) [36]	Longitudinal 28	1 mo, 3 mo, 6 mo, 9 mo leptin (concentrations)	1 mo, 12 mo, 24 mo BMI, body weight, body weight gain	(−) Association for leptin and BMI at 2 y (No) Association for leptin and body weight or body weight gain at all ages	None reported

Mohamad et al. Malaysia, 2018 (UMIC) [88]	Cohort 155	Birth and 2 mo adiponectin, leptin (concentrations)	Birth, 2 mo, 6 mo, 12 mo body weight, BMIZ	(−) Association for HM adiponectin and BAZ, body weight and abdominal circumference at 2 mo . (No) Association for HM leptin and anthropometrics (No) Association found for maternal HM adiponectin at birth and 2 mo with adiposity at 6 and 12 mo . (−) Association for HM adiponectin at 2 mo and adiposity at 2 mo (No) Association for maternal HM adiponectin and abdominal circumference	Gestational weight gain, gestational age; maternal age, pre-pregnancy BMI; infant sex, BF patterns, BF exclusivity (exclusive, partial or no BF)

Nikniaz et al.Iran, 2013 (LMIC) [89]	RCT 80 (75 analyzed)	30 d postintervention TAC, MDA (concentrations)	Pre and postintervention BMI, WAZ, HAZ, HC	(No) Significant association was found for weight for Z-score of infants and TAC and MDA levels in HM	Infant birth weight, maternal BMI, maternal daily energy intake

Nuss et al.USA, 2019 (HIC) [65]	Cross-sectional 33	One sample, between 4 wk and 8 wk leptin, insulin, TNF- α, IL-6 (concentrations)	Same as milk sampling weight, length, HC, % fat mass	(−) Association for leptin and weight (−) Association for leptin and HC (−) Association for leptin and % fat mass (No) Association for leptin and length (−) Association for Insulin and weight (−) Association for Insulin and HC (−) Association for Insulin and % fat mass (No) Association for Insulin and length (+) Association for TNF-α and weight (−) Association for TNF- α and HC (+) Association for TNF- α and % fat mass (No) Association for TNF- α and length (−) Association for IL-6 and weight (−) Association for IL-6 and HC (−) Association for IL-6 and % fat mass (−) Association for IL-6 and length	Infant age at visit

Ortiz-Andrellucchi et al.Spain,2008 (HIC) [37]	RCT 104: 45 placebo (39 analyzed) + 59 treatment (54 analyzed)	72 d, 10 d, and 45 d TGF-β 1, TGF-β 2, IL-1B, IL-6, IL-8, IL-10, IL-12, TNF- α (concentrations)	Birth, 2 mo, 6 mo weight	No significant differences for groups in relation to weight (data not shown)	None reported

Pundir et al.Australia, 2020 (HIC) [90]	Cohort 18	2 mo, 5 mo, 9 mo, 12 mo leptin (concentrations)	2 mo, 5 mo, 9 mo, 12 mo HC, % FM, length, weight, BMI	(+) Association for cortisol and HC (+) Association for cortisol and %FM (No) Association for cortisol and length, weight, and BMI (NO Association for cortisone and any parameters (+) Association for cortisol/cortisone ratio and %FM (+) Association for cortisol/cortisone ratio and BMI	None reported

Quinn et al.Nepal, 2017 (LMIC) [91]	Cross-sectional 50 from Nubri (NV) 66 from Kathmandu (K)	NV=11.02 mo ±7.66; K=11.70 mos ± 8.44 adiponectin, Leptin (concentrations)	NV=11.02 mo ±7.66; K=11.70 mos ± 8.44 weight, length, HC, WAZ	(No) Association for Milk leptin with WAZ (NV) (−) Association for milk leptin with WAZ (K) (No) Association for adiponectin and WAZ (NV) (−) Association for adiponectin and WAZ (K)	Infant age, transferred milk volume, sex, birth order, nursing frequency and, in the Nubri Valley subset, altitude of residence.

Saben et al.USA. 2021 (HIC) [92]	Cohort 194	2 mo HMOs (see Supplemental Table 4) (concentrations and intakes)	2 mo, 6 mo weight, length, WLZ, WAZ, FM, FFM	All s: (+) Association for 3’FL and fat mass, WLZ, and WAZ at 2 mo–6 mo (+) Association for LNFP II and fat mass, WLZ and WAZ at 2 mo–6 mo (+) Association for LNFP III and fat mass at 2 mo–6 mo (+) Association for 3'SL and fat mass and WAZ at 2 mo–6 mo (+) Association for 6'SL and fat mass at 2 mo–6 mo (+) Association for LSTb and fat mass, WLZ and WAZ at 2 mo–6 mo (+) Association for DSLNT and fat mass at 2 mo–6 mo (+) Association for DSLNH and fat mass, WLZ and WAZ at 2 mo–6 mo (+) Association for Acidic HMOs and fat mass, WLZ, WAZ at 2 mo–6 mo (+) Association for Total HMOs and fat mass, WLZ, WAZ at 2 mo–6 mo EBF Infants only: (+) Association for 3’FL and fat mass and WAZ at 2 mo–6 mo (+) Association for LNFP II and fat mass, WLZ and WAZ at 2 mo–6 mo (+) Association for 3'SL and fat mass and WAZ at 2 mo–6 mo (+) Association for 6'SL and fat mass at 2 mo–6 mo (+) Association for LSTb and fat mass WAZ at 2 mo–6 mo (+) Association for DSLNH and fat mass, WLZ and WAZ at 2 mo–6 mo (+) Association for Acidic HMOs and fat mass and WAZ at 2 mo–6 mo (+) Association for Total HMOs and fat mass and WAZ at 2 mo–6 mo	Infant birth weight, sex, age at time of measurement; maternal BMI, mode of delivery, and secretor status; BF status

Saso et al.The Gambia, 2018 (LIC) [93]	Cohort 100 (subset of larger study)	Birth (colostrum), day 60–89 IL-1beta, IL-2, IL-4, IL-6, IL-10, IL-12, IL-13, IFN-gamma, TNF α, IGF-1, and TGF-β2 (concentrations)	Birth, day 60–89 change in WAZ since birth, WAZ at final visit	(+) Association for IL6 and WAZ at final visit (Adjusted) (−) Association for TNF- α and WAZ at final visit (Adjusted) Cytokine levels in mature HM were weakly predictive of poor growth, possibly reflecting a “read-out” of suboptimal maternal health and nutrition. When adjusted for maternal anemia (as a proxy for maternal nutrition), TNFα and IL6 remained significant predictors	None reported

Savino et al.Italy, 2012 (HIC) [94]	Cross-sectional 41	Between 0 mo and 6 mo leptin, resistin (concentrations)	Between 0 mo and 6 mo weight, length, BMI	(No) Associations for HM hormones and anthropometric parameters but no numerical data given	None reported

Schueler et al.USA, 2013 (HIC) [95]	Cohort 13	29 d–38 d hind milk and fore milk GLP-1, PYY, leptin (concentrations)	29d –38 d, 6 mo and 12 mo weight	(−) Association for hindmilk GLP-1 at 1 mo after delivery and weight gain over first 6 mo. (−) Association for GLP-1 and WFL percentile at 6 mo	None reported

Schuster et al.Germany, 2011 (HIC) [39]	Cohort 23	End of the first, second, third, and fourth wk followed by the second, third, fourth, fifth, and sixth mo PP leptin (concentrations)	End of the first, second, third, and fourth wk followed by the second, third, fourth, fifth, and sixth mo PP weight gain from birth to 6 mo, birth to 4 wk	(−) Association for leptin at 1 wk and weight gain from 1 mo to 6 mo; but not from 1 wk to 4 wk	None reported

Sims et al.USA, 2020 (HIC) [96]	Longitudinal 284 (174 analyzed)	Postnatal age 0.5 mo, 1 mo, 2 mo, 3 mo, 4 mo, 5 mo, 6 mo, and 9 mo Leptin, insulin, c-reactive protein, IL-6, IL-8, TNF- α (concentrations and intakes; associations for intakes)	Postnatal 0.5 mo, 1 mo, 2 mo, 3 mo, 4 mo, 5 mo, 6 mo, and 9 mo weight, length, LAZ, WAZ, WLZ, fat mass, fat-free mass, FMI, FFMI	(No) Association for Leptin and WFA & WFL (+) Association for Leptin and LFA (No) Association for Insulin and LFA, WFA & WFL (No) Association for C-reactive protein and LFA, WFA & WFL (+) Association for daily intake of insulin with FMI (−) Association for daily intake of leptin with FMI (No) Association for daily intake of CRP and FMI Analysis of effect of HM composition on growth, stratified by normal weight and overweight maternal BMI groups: - Effects of insulin on FMI driven by overweight group - Daily intake of CRP associated with FMI in overweight group, but not in normal weight group - Daily intake of CRP associated with FFMI in normal weight group, but not in overweight group (NO)	Infant sex, feeding mode (exclusive vs. mixed)

Sprenger et al.Singapore, 2017 (HIC) [97]	Longitudinal 50	30 d, 60 d, 120 d 2'FL, 3'SL, 6'SL, LNnT, LNT (concentrations)	Birth, 1 mo, 2 mo, 4 mo weight, length, BMI, HC (WHO standards)	1(No) Association for milk type (low 2'FL vs. high 2'FL) and body weight, length, BMI, and HC over the 4 mo	Lactation stage, 2'FL status, infant sex

Tonon et al.Brazil, 2019 (UMIC) [98]	Cross-sectional 78	Once between 17d and 76 d of life HMOs (see Supplemental Table 4) (concentrations)	Same as milk sampling weight, length, weight gain	Based on Se and Le status: For Se+Le+: (−) Association for LNDFH I, LNT + LNnT, 3'SL, 6'SL, LSTa, LSTb, LSTc acidic HMOs, total acidic, total neutral core, total fucosylated, total HMOs and weight (−) Association for LNT + LNnT, 3'SL, LSTa, LSTc, total neutral core, total acidic HMOs and length (−) Association for LNDFH I, 6'SL, LSTc, total fucosylated, total acidic, total HMOs and weight gain For Se+Le-: (−) Association for 3'SL and weight (−) Association for 6'SL and length (−) Association for LSTb and weight gain For Se-Le+: (No) Associations For Se+ only: (−) Association for LNFP I, LNT + LNnT, 3'SL, 6'SL, LSTa, LSTb, LSTc (acidic HMOs), total fucosylated, total neutral core, total acidic, total HMOs and weight (−) Association for LNT + LNnT, 3'SL, 6'SL, LSTc, total acid HMOs and length (−) Association for LNFP I, LNFDH I, 6'SL, LSTc, total fucosylated, total acidic, total HMOs and weight gain For Se- only: (−) Association for 6'SL, LSTc and weight (−) Association for 6'SL, LSTc and length	None reported

Ucar et al.Turkey, 2000 (UMIC) [40]	Cross-sectional 18	Once, between 3 d and 120 d old leptin (concentrations)	Same as milk sampling weight, BMI, triceps skinfold thickness, left upper arm circumference measurements	(No) Association for log leptin concentration and 's body weight, BMI, triceps skinfold thickness, and left upper arm circumference measurements	None reported

Uysal et al.Turkey, 2002 (UMIC) [41]	Cross-sectional 50	68 d–126 d leptin (concentrations)	68d –126 d BMI	(No) Association for leptin and BMI	None reported

Van Rossem et al.Netherlands, 2019 (HIC) [99]	Longitudinal 251 (223 analyzed)	2 wk–25 wk adiponectin (concentrations)	3 mo, 1 y, 2 y BMIZ, WAZ, LAZ	(−) Association for adiponectin and BMIZ and WAZ at 3 mo (−) Association for adiponectin and weight gain for birth and 3 mo (−) Association for adiponectin and weight and height for 1 y	Maternal age, pre-pregnancy BMI, gestational weight gain; infant age at HM collection, sex, age at weight measurement, presence of siblings, birthweight

Wang et al.China, 2020 (UMIC) [100]	Longitudinal 269 (116 analyzed)	4 points: 1 d–5 d, 8 d–14 d, 4 wk (27 d–33 d), and 6 mo (177 d–183 d) HMOs (see Supplemental Table 4) (concentrations)	Same as milk sampling 1 d–5 d, 8 d–14 d, 4 wk (27 d–33 d), and 6 mo (177 d–183 d) weight gain, length gain	For secretors: (+) Association for length gain at mo 1 LNH, LNnH, MFpLNH-IV, IFLNH-I AND DFLNH-a+c, colostrum, TFLNH-I AND DFLNH-b in transitional milk, and LNnH, MFpLNH-IV, IFLNH-III, TFLNH-I, TFLNH-II and DFLNH-b in mature milk For nonsecretors: (−) Association for 3’FL, LNDFH II and weight gain @ 6 mo (+) Association for LNT & LNnT and length gain 1 mo (+) Association for LNnH and length gain at 2 and 6 mo	Maternal secretor status

Weyermann et al. Germany, 2007 (HIC) [42]	Cohort 1066 enrolled (786 breastfeeding 6 wk postpartum; 767 provided milk; 674 children with follow-up at 2 y)	33 d–71 d adiponectin, leptin (concentrations)	6 wk BMI, overweight risk (German reference)	(+) Association for children who were breastfed for ≥6 mo and increased risk for overweight at the of 2 with increasing HM adiponectin levels; this risk persisted after adjustment for covariates. We found no clear relationship for risk of overweight and HM leptin levels	Maternal age, education, nationality, pre-pregnancy BMI, smoking; birthweight.

Wolfs et al. USA, 2021 (HIC) [101]	Cohort 58 (57 analyzed)	1 mo, 3 mo, 6 mo 12,13-diHOME (concentrations)	1 mo, 3 mo, 6 mo weight, length,BMI, body composition (WHO standards)	(−) Association for 12,13-diHOME and delta BMIZ over 6 mo (−) Association for 12, 13-diHOME and delta WLZ over 6 mo (−) Association for log[9,10-diHOME], but was weaker with log[12,13-epOME] and delta WLZ over 6 mo (−) Association for log[Lyso-PG 18:0] and 1-mo body fat percent (−) Association for succinate and 6-mo BMIZ (+) Association for purine nucleotides (for example, 1-methyladenosine, 7-methylguanine) and adiposity	Pre-pregnancy BMI, gestational weight gain, parity, infant sex, gestational age

Woo et al. 2009USA (HIC),Mexico (UMIC) [47]	Longitudinal 46 (45 analyzed) in USA + 277 (206 analyzed) in Mexico	USA: monthly ≤6 mo Mexico: ≥2 samples, anytime between 1 wk and 6 mo adiponectin (concentrations)	Monthly ≤6 mo weight, length, BMI, WAZ, LAZ, WLZ (WHO standards)	In cross-sectional analysis: (−) Association for adiponectin and WAZ at baseline, mo 1 and 3 (−) Association for adiponectin and WLZ at baseline, mo 1 and 3 In longitudinal analysis: (−) Association for milk adiponectin and WAZ (−) Association for milk adiponectin and WLZ (No) Association for milk adiponectin and length or LAZ	Cohort, sex, age cross-sectional analysis only: length (for WA z-score), weight (for LA z-score), mo, moˆ2, infant birth weight

Wren-Atilola et al. Guatemala, 2021 (LMIC) [43]	Cohort 140	<6 wk, 4 mo–6 mo IL-1beta, IL-6, IL-8, TNF-α, Na:K (concentrations)	<6 wk, 4 mo–6 mo HC, weight, length, WAZ, LAZ, HCAZ (WHO standards)	(+) Association for Na:K ratio and stunting, and LAZ before 6 wk (−) Association for Na:K ratio and HCAZ before 6 wk. (+) Associations for IL-8 and HCAZ (−) Association for milk IL1β and LAZ before 6 wk (+) Association for IL-1β and daily rate of increase in length from early to established lactation	Indicators of subclinical mastitis and breast inflammation, fecal oral contamination, and BF practices

Wu et al.China, 2021 (UMIC) [102]	Cohort 227 (129 healthy, 98 GDM; 100 analyzed)	1 d–3 d, 10 d, 42 d untargeted metabolomics (concentrations)	1 d–3 d, 10 d, 42 d body weight gain	(+) Association for unsaturated lipids eicosatrienoic acid (FA 20:3) and LysoPC (20:6) and body weight gain (−) Association for phosphocreatine, creatine, D-glutamic acid, N-methyl-Daspartic acid, L-serine, phosphocholine, iditol, sorbitol, galactitol, and cytarabine and body weight gain"	None reported

Yis et al.Turkey, 2010 (UMIC) [103]	Cohort 24	80 d–135 d ghrelin, leptin (concentrations)	80 d–135 d weight, length, HC, postnatal weight gain, TSF	(No) Association for ghrelin or leptin and anthropometrics	BF exclusivity

Çağiran Yilmaz et al.Turkey, 2021 (UMIC) [44]	Cohort 65	1 mo, 3 mo, 6 mo leptin (concentrations)	1 mo, 3 mo, 6 mo weight, length, HC, chest circumference (WHO standards)	(−) Association for 1 mo leptin and body weight at 6 mo (−) Association for 3 mo leptin and body weight at 3 mo and 6 mo (−) Association for 6 mo leptin and body weight at 3 mo and 6 mo (+) Association for 1 mo leptin and length at 1 mo, 3 mo and 6 mo (+) Association for 3 mo leptin and length at 1 mo, 3 mo and 6 mo (+) Association for 6 mo leptin and length at 1 mo, 3 mo and 6 mo	None reported

Yu et al.Beijing, 2018 (UMIC) [104]	Longitudinal 121 (96 analyzed for day 3 colostrum, 78 for day 42 mature milk, 61 for day 90 mature milk)	Colostrum on day 3, mature milk on day 42 and 90 adiponectin, leptin, Insulin, ghrelin (concentrations)	Days 3, 42, 90 weight, length, HC, WFL gain	(−) Association for overall adiponectin during first 3 mo and WFL in both GDM and healthy groups (−) Association for adiponectin, insulin and HC during follow-up period (that is, after day 3), but insulin was insignificant after Bonferonni correction (−) Association for day 90 adiponectin and WFL in GDM group (+) Association for day 90 adiponectin and WFL in healthy group	Maternal gestational diabetes status

Zamanillo et al. Spain, 2019 (HIC) [46]	Longitudinal 59 (38 normal weight, 21 overweight/obese)	30 d, 60 d, 90 d leptin, adiponectin (concentrations)	30 d, 60 d, 90 d BMI	(No) Associations for HM analytes of interest and anthropometrics	Maternal BMI

Alternative versions organized by component are available in Supplemental Tables 3–5.

Abbreviations: AFABP, Adipocyte-Specific Fatty Acid-Binding Protein; BF, breastfeeding; BMIZ, BMI-for-age Z-score; CDI, calculated daily intake; HIC, high-income countries; HM, human milk; HMO, human milk oligosaccharide; LMIC, low- and middle-income countries; MDA, malondialdehyde; NCHS, National Center for Health Statistics; RCT, randomized controlled trial; SCM, subclinical mastitis; SFT, skinfold thicknessWFA, weight-for-age; TAC, total antioxidant capacity; WFAZ, weight-for-age z-score; UMIC, upper middle-income countries.

Anthropometrics: FFM, fat-free mass; FMI, fat mass index; FFMI, fat-free mass index; HAZ, height-for-age z-score; HC, head circumference; HCAZ, head circumference z-score; LAZ, length-for-age Z-score; WAZ, weight-for-age z-score; WLZ, weight-for-length z-score.

Components: 2’FL, 2’fucosyllactose; 6’SL, 6’sialyllactose; DFLac, difucosyllactose; DFLNT, difucosyllacto-N-tetrose; DFLNH, difucosyllacto-N-hexaose; DSLNT, disialyllacto-N-tetraose, cGP, cyclic glycine-proline; sCD14, soluble cluster of differentiation 14; GLP, glucagon-like peptide; IFLNH-I, fucosyl-para-lacto-N-hexaose I; IGF-1, insulin-like growth factor 1; LNFPI, lacto-N-fucopentaose I; LNFP II, lacto-N-fucopentaose II; LNFP III, lacto-N-fucopentaose III; LNH, lacto-N-hexaose; LNT, lacto-N-tetrose; LNnT, lacto-N-neotetraose; LSTa, sialyl-lacto-N-tetraose a; MFpLNH-IV, monofucosyl-para-lacto-N-hexaose IV; PYY, peptide YY (also known as peptide tyrosine tyrosine); TFLNH, trifucosyllacto-N-hexaose; TNF-α, tumor necrosis factor – alpha; TGF-β, transforming growth factor – beta.

1 Indicates data were provided by the study author and do not appear in the referenced publication.

2 No (assumed) associations = unreported associations assumed to be no association.

### Study quality

**FIGURE 2:**
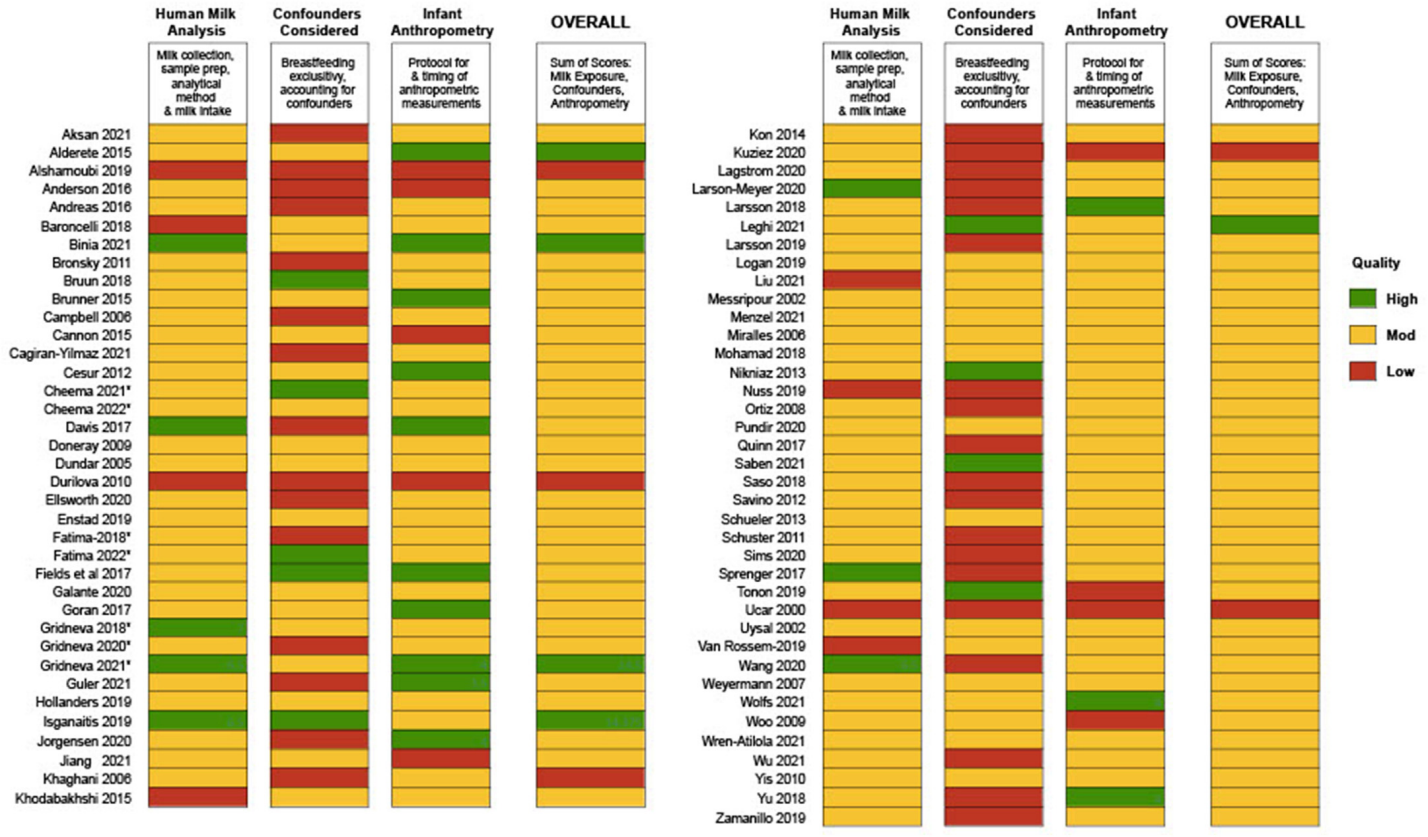
Summary of quality assessments of included articles. Quality scores are awarded based on the number of points assigned according to the criteria in Supplemental Table 1. Detailed numeric scores are presented in Supplemental Table 2. HM. HM, human milk. ∗Indicates the same study, but separate articles.

The majority of studies (*n =* 65) were rated as moderate (8.5–12.75 score on the modified Newcastle–Ottawa scale; maximum 17 points), with 5 studies being rated as low quality (<8) and only 5 studies rated as high quality (>13) (Figure 2, Supplemental Table 2). The most common quality issue across studies was failing to adjust for confounders, such as breastfeeding exclusivity, maternal BMI, or maternal age.

A variety of infant anthropometrics were reported across studies. Weight and length were the most common anthropometrics reported for each of the bioactive categories. Among studies examining hormones, 24 different anthropometrics were reported, compared with 22 for HMOs and 26 for immunomodulatory components.

Milk collection strategies and time points varied considerably across studies. Thirty articles reported analyte concentrations in milk from a single collection time point. Only 10 articles reported intakes and many of these were from the same research group [[57], [60], [71]]. In addition, there was substantial variation in how 24-h milk intake was assessed. Most studies used pre- and post-feed weights; however, some did this for each feed over 24 h [[60], [83]] whereas others only weighed at 1 feed and multiplied it by the number of feeds [84]. Milk sampling times varied from birth (colostrum) to 4 y (which was outside the scope of this study). The most common time points for milk sampling were 1 mo (30 articles), 2 mo (18 articles), 3 mo (19 articles), and 6 mo (21 articles). Twelve studies reported “varied” milk collection time points which ranged from 10 d to 4 y postpartum. Two studies [57,77] did not report when milk samples were collected.

Heterogeneity in infant anthropometrics and variation in milk sampling procedure and collection time limited the ability to compare across a wide range of studies. Because of heterogeneity in study designs, sampling times, and reporting practices, meta-analyses and pre-planned subgroup analyses (for example, by study setting, mode of HM feeding, and nutritional status of mothers) were not feasible. The full list of pre-specified subgroups is available in the review protocol [6].

### Associations of HM bioactive and infant anthropometry

HM bioactives from 66 of 75 articles fit into the 3 overarching categories: hormones, HMOs, and immunomodulatory components (described below). Among the other 8 studies, infant growth was positively associated with osteopontin [26], 12,13-diHOME [101], and chemerin [67]. No associations were found for milk epidermal growth factor [79], total antioxidant capacity [89], sterol regulatory element binding protein 1 [66], and soluble cluster of differentiation 14 [52].

### Hormones

**FIGURE 3:**
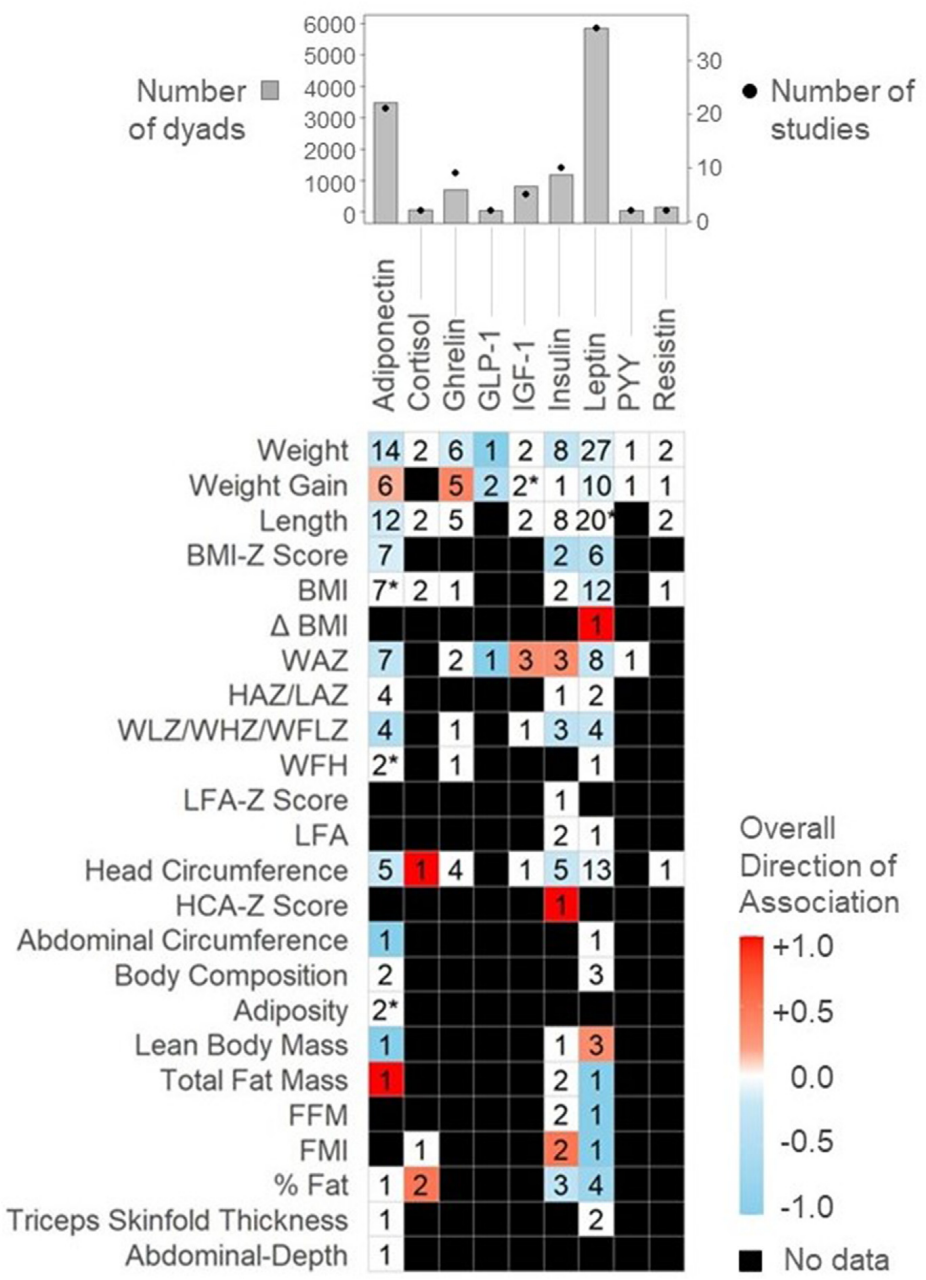
Mean directions of associations between HM hormones and infant growth in the first 2 y. Significant associations reflect results as reported by individual study authors (for example, using HM concentrations as the predictor variable, see Table 1). Value in cells indicates the number of studies examining each comparison. Red squares indicate mean positive associations, blue squares indicate mean inverse associations, white squares indicate a mean association of 0, and black squares indicate that association was not assessed. HCA, head circumference-for-age; HM, human milk; LFA, length-for-age; WFA, weight-for-age; WFL, weight-for-length; BFA, BMI-for-age; FFM, fat-free mass; FMI, fat mass index. ∗Indicates that equal numbers of positive and negative associations were observed, resulting in a gradient of zero (0).

Hormones were the most extensively examined bioactive in the body of literature with 46 articles (*n =* 6773 dyads) exploring how hormone concentrations were related to infant growth (Figure 3, Supplemental Table 3). Overall, 13 hormones were examined with leptin being the most common (*n =* 35 articles; 5857 dyads), followed by adiponectin (*n =* 18 articles; 3479 dyads). Other hormones studied included cortisol, ghrelin, glucagon-like peptide-1 (GLP-1), insulin-like growth factor 1 (IGF-1), insulin peptide tyrosine tyrosine (PYY), and resistin. Notably, of the 46 included articles, only 15 accounted for maternal pre-pregnancy BMI. This is an important consideration because maternal BMI appears to impact several appetite-regulating milk hormone concentrations, including leptin and adiponectin [105]. However, BMI is subject to bias as it is often self-reported and thus inaccurate and the relevance to HM composition is relevant at milk sampling time rather than before pregnancy [106]. Finally, there were inconsistencies across studies regarding milk preparation before analyzing hormone levels. Using skim milk compared with homogenized milk is an important consideration as lipids may interfere with certain hormones and assays, especially when examining leptin and adiponectin levels in HM [107]. We identified inconsistencies between studies with some using whole milk samples [70,65] and other studies using skim milk samples [68,55] to test for adiponectin and leptin levels.

#### Leptin.

Overall, HM leptin concentrations were negatively associated with infant anthropometry in more than half of the comparisons (11/20 comparisons), including for weight, weight gain, length, BMIZ and percent fat (Figure 3). Most remarkably, 3 of 4 studies examining the association between leptin and percent fat mass found significant inverse associations (Figure 3) [65,68,108], whereas 2 studies observed positive associations for lean body mass [70,55]. The 1 exception to this trend was a positive association with change in BMI over the first 2 y in infants born with an average BMI [85]; however, only 1 study evaluated this association. Two studies consistently showed no association between milk leptin and triceps skinfold thickness [40,83]. Four studies [96,[60], [83], [84]] examined CDI of HM leptin and none found meaningful associations with infant growth outcomes despite 3 of these studies [[60], [83], [84]] using the same technique to calculate CDI.

#### Adiponectin.

Similar to leptin, HM adiponectin concentrations were overall inversely associated with infant growth, with weight and length being reported most frequently. Multiple studies found inverse associations between milk adiponectin concentrations and infant weight [77,[54], [88], [99]] and length [99,54], and weight-for-age Z-Score (WAZ) [47,99,91]. Only 3 of 21 studies [71,54,104] found positive associations between milk adiponectin and infant growth outcomes. However, Yu et al. [104] stratified by healthy mothers and mothers with gestational diabetes, and only found a positive association with infants from healthy mothers. Gridneva et al. [70], found an inverse association with infant abdominal adiposity; however, this was a time-dependent relationship calculated from daily intake rather than a cross-sectional 1-time adiponectin measurement.

#### Other hormones.

Milk insulin concentrations were examined in 9 studies (10 articles) with 1189 dyads. All studies, except Chan et al. [59] and Yu et al. [104] excluded mothers with diabetes; both of which found that HM insulin concentrations were significantly higher in mothers who were diagnosed with diabetes. Overall, inverse relationships were observed between milk insulin and infant growth; specifically with infant weight [108,51], head circumference [108,104], BMIZ [59], and percent fat [108]. However, 8 of 10 studies found no relationship between milk insulin and weight and no studies found associations between milk insulin and infant length. Conversely, Sims et al. [96] and Ellsworth et al. [64] found positive relationships between insulin and fat mass index (FMI), WAZ, and head circumference-for-age z-score. However, both of these studies stratified mothers by maternal BMI examining outcomes of infants consuming milk from normal weight compared with overweight mothers and neither study controlled for exclusive HM feeding.

Milk ghrelin was examined in 9 studies (701 dyads) and demonstrated minimal associations with infant growth outcomes. An inverse association was observed with infant weight in 1 study [77], whereas 5 other studies [104,51,72,103,58] found no association. Positive associations were observed for weight gain in 2 [58,78] of the 5 studies that reported an increased rate of weight gain when higher concentrations of milk ghrelin were present.

Milk IGF-1 was analyzed in 5 studies (820 dyads). No consistent associations were found with infant growth across all studies. Two studies demonstrated opposite trends between milk IGF-1 and infant weight gain [78,69]. However, Kon et al. [78], categorized infant weight gain into low weight gain (<500 g/mo), normal weight gain (between 500 and 1000 g/mo), and high weight gain (>1000 g/mo), whereas Galante et al. [69] treated weight gain as a continuous variable. In addition, the small molecule metabolite cyclic glycine-proline, which is derived from IGF-1, demonstrated a positive association with WAZ [73].

Milk cortisol and resistin each were analyzed in 2 studies, respectively. Pundir et al. [90] found positive associations between milk cortisol concentrations and infant head circumference and infant adiposity (measured by percent fat mass), whereas Hollanders et al. [73] found no associations between milk cortisol concentrations and any infant growth outcomes. Both studies controlled for percent breastfeeding at the time of milk collection. Milk resistin was also examined by [51,94] and no associations were found with infant growth outcomes in either study.

PYY and GLP-1 were both assessed by [95] and [81]. Although Schueler et al. [95] reported on a subset of the larger Larson-Meyer et al. cohort [81], they examined different infant growth outcomes. Both Schueler et al. [95] and Larson-Meyer et al. [81] found that GLP-1 concentrations were inversely associated with infant growth (weight, weight gain, and WAZ), but found no associations between PYY and infant growth outcomes.

Only 1 study explored sex hormones and infant growth [33]. Inverse associations were observed between infant weight at 6 mo and progesterone, luteinizing hormone, and follicle-stimulating hormone, but not estradiol. Finally, 1 study [66] explored the relationship between irisin and infant growth, observing a positive association between milk irisin and infant weight at 6 wk of age.

Overall, our systematic review indicates that milk leptin and milk adiponectin are the only hormones that have been consistently associated with infant growth, with higher concentrations of leptin typically being associated with fat deposition and lean body mass, and adiponectin being associated with reduced growth outcomes.

### HMOs

**FIGURE 4:**
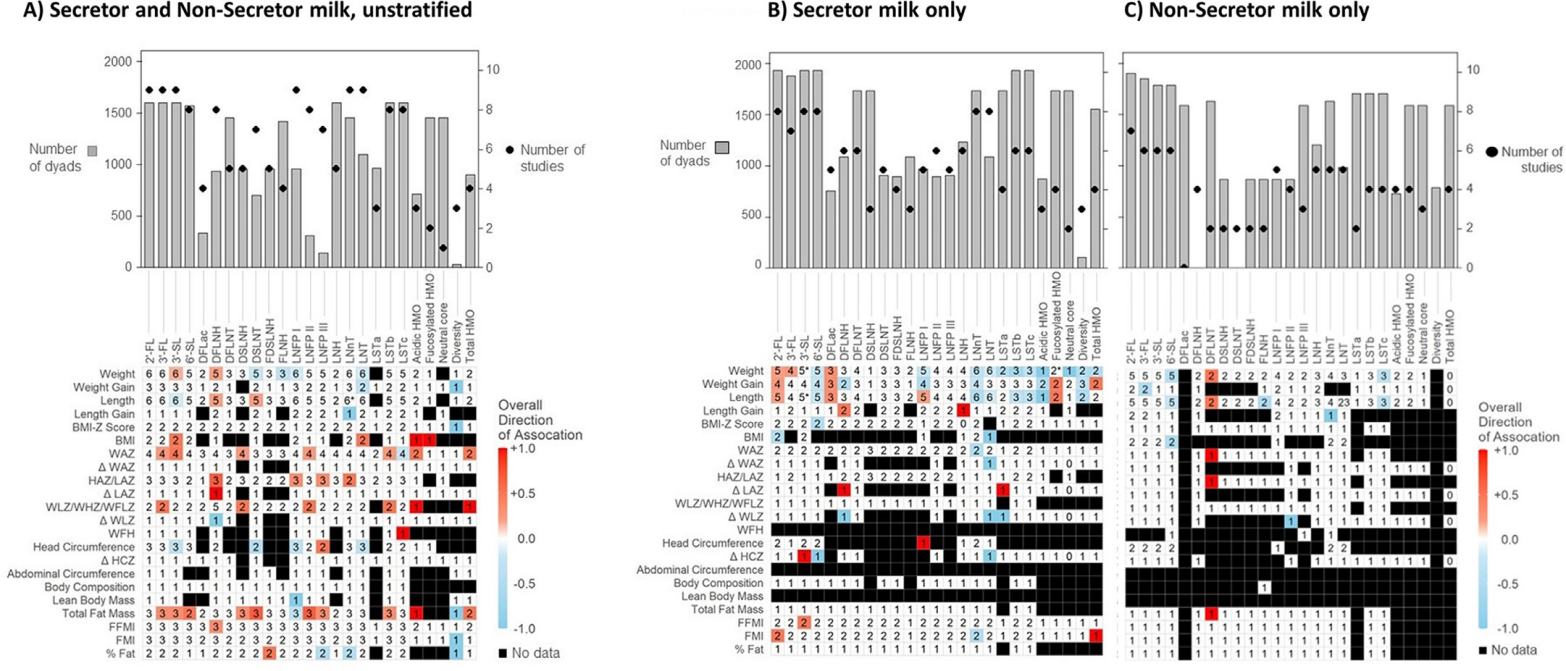
Mean directions of associations between HMOs and infant growth in the first 2 y, regardless of secretor status (A), or separately for secretor (B) and non-secretor (C) milk. Significant associations between HMOs and infant anthropometrics reflect results as reported by individual study authors (for example, using HM concentrations as the predictor variable, see Table 1). Value in cells indicates the number of studies examining each comparison. Red squares indicate mean positive associations, blue squares indicate mean inverse associations, white squares indicate a mean association of 0, and black squares indicate that association was not assessed. Note that some studies did not stratify on secretor status while others only presented stratified data (i.e. the studies in A are not all represented in B&C, and vice-versa). Despite using the 'near absence' of 2'FL to define non-secretor status, some studies still reported on 2'FL in this group, though no associations with infant growth were observed. HCA, head circumference-for-age; HM, human milk; HMO, human milk oligosaccharide; LFA, length-for-age; WFA, weight-for-age; WFL, weight-for-length; BFA, BMI-for-age; FFM, fat-free mass; FMI, fat mass index. ∗Indicates that equal numbers of positive and negative associations were observed, resulting in a gradient of zero (0).

In total, 13 studies (*n =* 2640 dyads) examined the association between HMO composition and infant growth (Supplemental Table 4, Figure 4). Some studies stratified all analyses on maternal secretor status [[80], [97], [98], [100]], a major genetic determinant of HMO composition, whereas others did not stratify [[48], [53], [62], [86], [92]], and some performed both stratified and combined analyses [[61], [76], [82], [87]]. Classification and identification of HMOs were inconsistent among studies, and there was considerable variation in which HMOs authors examined. All studies used some form of liquid chromatography to analyze HMOs. Lagstrom et al. [80], Alderete et al. [48], and Larsson et al. [82] used HPLC after fluorescent derivatization, resulting in a uniform comparison of individual HMOs and consistent measures of composite HMOs, whereas Jorgensen et al. [76] and Davis et al. [62] used an LC-MS (nano-LC-chip/time-of-flight mass spectrometry) approach resulting in the identification of several previously unnamed and unstudied HMOs. This difference in methodology made comparison between studies difficult. For example, Tonon et al. [98] analyzed 16 HMOs which they indicated were most the abundant, whereas Davis et al. [62] analyzed 39 HMOs. Jorgensen et al. [76] used the same analytical techniques as Davis et al. [62]; however, the infant anthropometrics examined were not comparable with other studies (change in LAZ, HAZ, WLZ, and HCZ).

#### Maternal secretor status

All 13 studies measured maternal secretor status in their analysis of HMO profiles and infant growth; however, only 8 studies reported outcomes stratified by secretor status [[80], [97], [98], [100],[61], [76], [82], [87]]. Tonon et al. [98] , Binia et al. [53], and Wang et al. [100] further stratified their analyses by Lewis status, which is a functional dominant allele that is coded on the FUT3 gene on chromosome 19 and has a lesser known impact on HMO patterns in HM [109]. Presence or near-absence of the HMO 2’Fucosyllactose (2'FL) was used across all studies to determine secretor status, with milk containing 2’FL being classified as secretor milk. Sprenger et al. [97] and Menzel et al. [87] compared infant growth between infants receiving secretor or non-secretor milk. Although Sprenger et al. [97] found no association between any infant growth parameters and secretor and non-secretor milk, Menzel et al. [87] found that infants who consumed milk from non-secretor mothers had significantly higher BMI at 3 mo and 6 mo. In addition, Menzel et al. [87] found statistically higher head circumference in infants consuming non-secretor milk at 3, 6, and 12 mo and that this difference was sustained until 7 y of age. These findings indicate a need to consider the secretor status of the mother when looking at growth outcomes overall. Notably, only Binia et al. [53] accounted for infant secretor status, which could be an important consideration because the infant’s own secretor status can also impact microbiome development and other aspects of physiology [76].

#### Combined secretor and non-secretor milk—individual HMOs

Nine studies (*n =* 1594 dyads) examined the association between individual HMO concentrations and infant growth with secretor and non-secretor samples combined (Figure 4A) [[48], [53], [61], [62], [76], [82], [86], [87], [92]]. There were no consistent associations between individual HMOs and infant growth outcomes observed across studies. Although lacto-N-fucopentaose I (LNFPI) demonstrated an inverse association with infant weight, total fat mass, and lean body mass, this was only observed in 1 of 6 studies reporting on these associations [48]. Conversely, Davis et al. [62], found a positive association with BMIZ, but only when looking at LNFP I and LNFP III in concert. Binia et al. [53] concluded that fat mass accretion and fat mass index did not appear to be related to a specific HMO and highlighted the importance of examining HMOs in composite groups rather than individually.

The majority of studies only analyzed HM component concentrations. However, Cheema et al. [61] also assessed the daily intake of HMOs, finding positive associations between 2’FL, 3’FL, difucosyllactose (DFLac), Sialyl-lacto-N-tetraose b (LSTb), and difucosyllacto-N-tetrose and infant growth, whereas these associations were not observed when simply measuring concentrations.

#### Secretor milk—individual HMOs

Eight studies (*n =* 1928 dyads) reported results for individual HMO concentrations in secretor milk and resultant associations, with lacto-N-neotetraose (LNnT) and lacto-N-tetrose (LNT) demonstrating the most consistent (inverse) relationships with infant growth (Figure 4B: Secretor HM). In 3 of 6 studies [80,98,48], LNnT demonstrated inverse associations with infant length and in 2 of 6 studies [92], LNnT demonstrated inverse associations with infant weight. Furthermore, inverse associations were observed between LNnT and weight velocity [82], WAZ and weight-for-height Z-Score (WHZ) [109], and fat mass index [82]. LSTb also demonstrated a consistent inverse association with infant weight across 2 of 3 studies reporting on this relationship [48]. Finally, 6’sialyllactose (6’SL) demonstrated a potential negative association with infant growth, though only 1 of 5 studies observed this inverse association with weight, weight gain, and length [61].

Several positive associations were identified between secretor milk 2’FL, 3’FL, DFLac, lacto-N-hexaose, and difucosyllacto-N-hexaose concentrations and infant growth outcomes (Figure 4B). However, these positive associations were only identified in 1 study each and were not replicated in other studies. 2’FL and DFLac were positively associated with infant weight [76], length [80,82], and weight velocity [82]. Larsson et al. [82] also found an additional positive association between 2’FL and fat mass index. Finally, using the untargeted method of HMO identification Jorgensen et al. [76] found positive associations between unnamed HMOs 5130b and HMO 4240a and change in LAZ as well as a positive association between the composite of 5230a + DFLNnO I/DFLNO II (Difucosyllacto-N-neooctaose I/difucosyllacto-N-octaose II) and change in WAZ. Wang et al. [100] identified unique positive associations between monofucosyl-para-lacto-N-hexaose IV, fucosyl-para-lacto-N-hexaose I, trifucosyllacto-N-hexaose and infant length gain.

Mixed associations were observed between 3’SL and infant weight and length, with Lagstrom et al. and Larsson et al. [80,82] finding positive associations and Tonon et al. [98] finding negative associations. Although stratification among infants receiving only milk from secretor mothers showed increased significant associations with infant growth compared with combined analyses, these associations were only found in single studies for most individual HMOs.

#### Non-secretor milk—individual HMOs

Seven studies (*n =* 1782 dyads) reported results for individual HMOs from infants receiving milk from non-secretor mothers (Figure 4C) [[60], [76], [80], [87], [97], [98], [100]], which make up a relatively smaller proportion of the population. Typically, 6%–24% of populations in European and North and Latin American Countries and as many as 29%–51% of populations in some South Asian countries are nonsecretors [111]. Because of the lower proportion of nonsecretors in the population, many of these studies may not be sufficiently powered to detect statistical differences in non-secretor participants only. Again, associations between individual HMOs and infant growth outcomes were typically found in single studies and consistent associations between studies were not observed. Lagstrom et al. [80] and Jorgensen et al. [76] did not find any associations between individual HMOs in non-secretor milk and infant anthropometrics. Tonon et al. [98] and Lagstrom et al. [80] found that a concentration of 6’SL in HM had an inverse association with infant weight and Menzel et al. [87] found an inverse association with BMI. However, when Cheema et al. [60] accounted for intakes (not shown in heatmaps) instead of milk concentration, they found a positive association between 6’SL and infant weight. As such, it is hard to draw meaningful conclusions about the association between 6’SL and infant growth outcomes.

#### Composite HMO measures

Composite measures of HMOs, such as acidic or fucolsylated HMOs, tended to demonstrate inconsistent directional associations with infant growth across studies. HMO diversity showed inverse associations with several infant growth outcomes for both secretor-only and combined secretor and non-secretor milk (Figure 4A and B). Inverse associations were observed for HMO diversity and BMIZ [82], total fat mass [48], fat mass index [82], and percent fat [48] in analyses combining secretors and nonsecretors; whereas weight and length [76] demonstrated inverse associations when the analysis was restricted to just secretor milk. Acidic HMOs demonstrated positive associations when analyses combined secretors and nonsecretors, but inverse associations when analyses were restricted to only secretor milk.

Composite HMO measures also revealed an inverse association between total HMOs and infant weight in non-secretor milk [111,112]. No other meaningful associations were observed between composite HMOs in non-secretor milk and infant growth outcomes across studies. Finally, Jorgensen et al. [76] found inverse associations between composite HMO measures of LNT + LNnT and LNFP I + III and change in WLZ in infants consuming secretor milk.

Jiang et al. [75] explored infant growth patterns by combining proteome, lipidome, and HMO data using principal component analysis (PCA) rather than looking at individual HMOs. Using this method, they found that the group high in LNnH (lacto-N-neothexaose)and LNDFH II (lacto-N-difucohexaose) and low in 3’SL was inversely associated with infant length-for-age (LFA) scores when adjusted for infant age, infant sex, birth weight, birth length, and maternal age. They also found that the factor high in LNnH , LSTa, LSTb, LSTc, 3’FL, and 2’FL and low in disialyllacto-N-tetraose was inversely associated with infant BMIZ. Noting that these factors also included proteomics and lipidomics, Jiang et al. [75] was the only study to look at multiple aspects of HM composition and infant growth outcomes using PCA.

The studies examining HMOs and infant growth included in this review had considerable variation in analytic techniques, study settings, and infant feeding status. For example, some studies [82] examined outcomes in exclusively breastfed infants whereas others did not account for breastfeeding exclusivity [48], which is an important predictor of HMO composition [113]. In addition, the lactation stage when samples were collected can also impact HMO composition, with 6'SL concentrations dropping quickly and 3’FL increasing over time. Sample collection ranged from 2 d postpartum [75] to 9 mo postpartum [82], which could result in important variability in HMO concentrations. Finally, the setting is an important influential factor in HMO composition [76] and HMO studies ranged across all 6 habitable continents in this review. As such, it is difficult to draw clear conclusions about the role of HMOs on infant growth because of the likely and unaccounted variability in HMO composition.

### Immunomodulatory components

**FIGURE 5:**
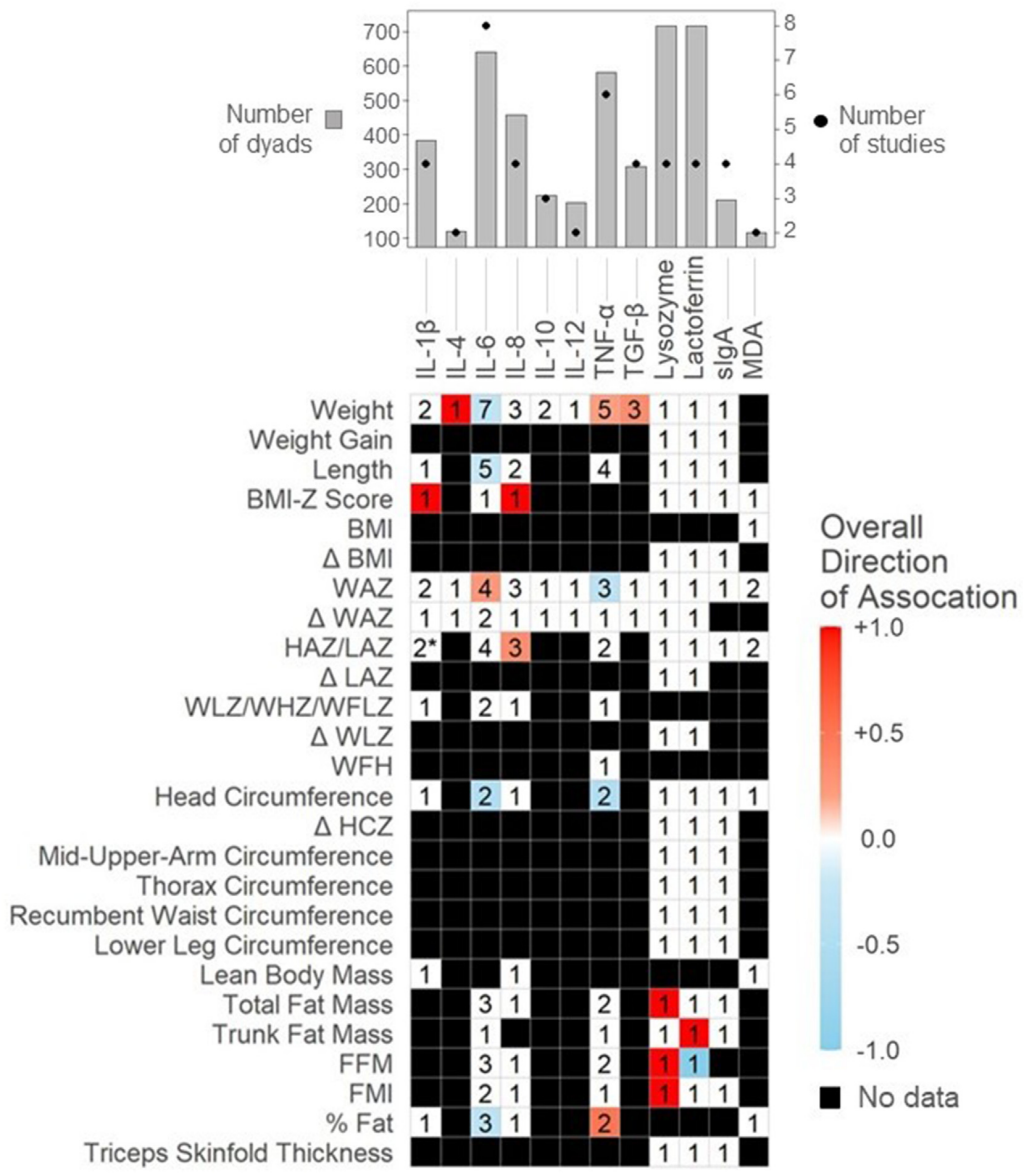
Mean directions of associations between HM immunomodulators and infant growth in the first 2 y. Significant associations between immunomodulators and infant anthropometrics reflect results as reported by individual study authors (for example, using HM concentrations as the predictor variable, see Table 1). Value in cells indicates the number of studies examining each comparison. Red squares indicate mean positive associations, blue squares indicate mean inverse associations, white squares indicate a mean association of 0, and black squares indicate that association was not assessed. HCA, Head circumference-for-age; HM, human milk; LFA, Length-for-age; WFA, weight-for-age; WFL, weight-for-length; BFA, BMI-for-age; FFM, fat-free mass; FMI, fat mass index. ∗Indicates that equal numbers of positive and negative associations were observed, resulting in a gradient of zero (0).

Twelve studies (*n =* 1422 dyads) were included in the immunomodulatory component category (Supplemental Table 5, Figure 5). Thirty-one anthropometrics were examined across the 12 studies, with weight and length being the most common. Bioactive components in the immunomodulatory category included cytokines, immunoglobulins, lactoferrin, lysozyme, and malondialdehyde. The area of investigation surrounding many of these HM components is still highly exploratory with studies using varied assays and minimal consensus on quality control. IL-6 (*n =* 7; *n =* 501 dyads) and tumor necrosis factor-alpha (TNF-α; *n =* 5, *n =* 441 dyads) were the most investigated bioactive components in this category. Interestingly, 10 of 12 studies were published after 2017, indicating that this is a relatively new area of exploration.

Nuss et al. [108] and Durilova et al. [63] both observed inverse associations between milk IL-6 concentrations and infant weight. Furthermore, Nuss et al. [108] found additional inverse associations between IL-6 and infant length, head circumference, and percent body fat. WAZ was the only growth outcome to demonstrate a positive association with milk IL-6 concentrations [93]. Among the ILs, IL-6 was the only component where >1 study found similar directional (inverse) associations between milk concentrations and infant growth outcomes (weight) [108,63]. Associations were only observed in one study each for all other IL milk components.

Six studies explored the relationship between milk TNF-α concentrations and infant growth. Inverse associations were observed between milk TNF-α and infant WFA [93] and infant head circumference [108]; whereas positive associations were observed for weight and percent fat mass [108].

Lactoferrin demonstrated mixed directional associations with infant growth within the same study. Gridneva et al. [71], observed a positive association with trunk fat mass and an inverse association with fat-free mass index, indicating that lactoferrin may have a positive association with overall infant body fat composition. Significant positive associations were found between infant growth outcomes and IL-1 β (BMIZ) [65], IL-4 (weight) [63], transforming growth factor – beta (weight) [49], and lysozyme (Fat-free mass, fat mass index, and total fat-free mass) [56]. However, these significant associations were only observed in 1 study each and findings were not replicated elsewhere.

### Metabolomics—untargeted analysis

Untargeted metabolomics analyses facilitate the observation of a broader range of compounds and report a relative abundance of molecules. Two studies [74,102] conducted untargeted metabolomics on bioactive components in HM and examined how these metabolites related to infant growth. In their case-control study of Chinese females with normal and gestational diabetic pregnancies, Wu et al. [102] identified 620 metabolites. Ten of these compounds (phosphocreatine, creatine, D-glutamic acid, N-methyl-D-aspartic acid, L-serine, phosphocholine, iditol, sorbitol, galactitol, and cytarabine) had significant inverse associations with infant weight gain and 2 (the lipids eicosatrienoic acid and lysophosphatidylcholine) had positive associations. Direct comparisons between Wu et al. [102], which looked at infant body weight gain as their infant growth metric, and Isganitis et al. [74], which examined infant fat mass percentage and fat accrual and excluded mothers with gestational diabetes, are obfuscated by differing growth outcomes as well as different chromatographic separation methods (liquid compare with gas chromatography). Indeed, when comparing the compounds in Wu et al. [102] to the compounds found to have significant associations with infant growth in Isganaitis et al. [74], there was no overlap of specific metabolites, which is not unexpected given the different data collection methods. However, at a broader metabolic level, there is evidence for similar biological effects in the creatine degradation pathway (negatively associated in both studies; phosphocreatine, creatine, and creatinine), and a general role for bioactive lipids—observed in both studies. A key component in fatty acid metabolism, carnitine, was negatively associated with infant fat accrual and infant fat mass percentage; however, it was positively associated with infant percent body fat.

### Other bioactive components

Several bioactive components, particularly bioactive lipids, did not fit into the aforementioned categories. Bioactive lipids play a prominent role in immune regulation, inflammation, and homeostasis [114]. In addition to lipids observed in the untargeted metabolomics experiments referenced above, bioactive lipids or bioactive lipid mediators involved in satiety, the N-acylethanolamine (NAE) lipids oleoylethanolamide, stearoylethanolamide, and palmitoylethanolamide, have been specifically investigated [56]. Stearoylethanolamide concentration was shown to have a negative association with both triceps’ skinfold thickness (β = −2.235, *P* = 0.016) and weight gain per day since birth (β = −8.169, *P* = 0.024) [56]. In Isganaitis et al. [74], NAE lipid 1-palmityolpasmenylethanolamine was negatively associated with infant weight.

## Discussion

### Key findings

The findings from this systematic review revealed inconsistent associations between HM bioactive components and infant body composition in the first 2 y, highlighted inconsistent data collection methods and identified many knowledge gaps for future research. Among bioactive constituents of HM, the largest body of evidence (spanning >20 y, hundreds of studies, and over 5000 dyads) exists for leptin and adiponectin, showing consistent inverse associations with infant growth across studies, although several studies found no association. In contrast, research on other hormones, HMOs, and immunomodulators has primarily emerged in the last decade, with data often limited to a few hundred infants. There were no consistent associations found between individual HMOs and infant growth outcomes across studies, partially because of differences in laboratory methodology and data analysis strategies. However, examining HMOs in concert with each other revealed more consistent associations across studies. Among all immunomodulatory components, IL-6 appeared to demonstrate the most consistent association with infant growth, showing an inverse relationship. In addition, metabolomic analysis of HM is also a new area of exploration and could yield many important relationships between HM metabolites and infant growth. Targeted analyses of metabolites in milk indicate that bioactive lipids are a broad group of molecules with diverse physiological effects, which remain to be elucidated in HM. However, consistency of assays and methodological quality control is still of concern in these emerging areas when considering the validity of outcomes. As such, it is important to acknowledge these limitations when examining immunomodulatory components in HM as they are likely impacted by the different methodological strategies and may not demonstrate consensus among studies.

Moving forward, researchers aiming to examine the association between components in HM should continue to focus on appetite-regulating hormones while expanding exploration to other hormones and composite analysis of HMOs as the number of observations for these components are still quite small. Furthermore, the relationship between immunomodulatory components in HM and infant growth is still preliminary and warrants further research through both targeted and untargeted explorations. In addition, it is important to think beyond examining components within the silos of macronutrient, micronutrient, and non-nutrient categories and consider how these components work in combination with each other.

### Improving the quality of HM research

Our ability to synthesize and interpret research findings was tempered by substantial variation in HM sampling times and collection strategies. Inconsistent findings related to HMO composition and infant growth were similarly related to inconsistent sampling time points and reporting in a recent review of microbiome-related products in HM and infant growth [115]. It is well established that HM composition changes throughout the day and over the course of lactation for some components [116]. Previous research examining the impact of circadian rhythms on bioactive components in HM has been inconsistent; likely because of methodological issues with milk collection and sampling strategies [116]. The impact of temporal variance in milk composition should be considered when developing milk collection strategies. Consistent collection times across studies that align longitudinally with the lactation stage and temporally with circadian rhythms will allow for more consistent comparisons of findings.

Extensive variation in the anthropometric outcomes measured across studies further complicated this review. Most studies examined length and weight; however, an additional 24 different anthropometric measures were also reported across studies, making it difficult to synthesize results. Reliability and reproducibility have long plagued infant growth research [117] and our results demonstrate that this issue persists with extensive variation even among many studies published within the last 5 y. Clinicians and researchers currently have over 100 growth or size charts from which to reference based on their location and local guidelines [118] and there are no universally accepted guidelines to identify clinically important growth trajectories [117]. This lack of consistency and guidance complicates the identification of meaningful research outcomes and the resultant translation to clinical practice. In our systematic review, only 4 studies [59,64,81,97] explicitly stated they were following WHO Child Growth Standards [113], to guide their anthropometric outcomes which are considered to be the gold standard for assessing child growth [[117], [119]]. Over 25 different anthropometric measures were reported across all included studies which challenged our ability to conduct a meta-analysis and compare outcomes across studies. Aris et al. [117] highlights the importance of using longitudinal growth trajectories, specifically BMIZ, rather than cross-sectional assessments to identify clinically meaningful trajectory patterns, risk for obesity, and adverse health outcomes. Consistent reporting of anthropometric measures, such as BMIZ will align research outcomes in a more understandable and meaningful way so that researchers and clinicians can guide their practice based on consistent outcomes.

Specific to studies examining HMOs, stratification by secretor status is an important consideration necessary to identify certain associations between HMOs and infant growth. Larsson et al. [82] and Jorgensen et al. [76] presented results stratified by secretor status and with combined secretor status; both identified associations for secretor-positive milk that were not present when combining secretors with nonsecretors. These differences indicate the vital importance of stratifying results by secretor status and to adequately power studies for stratified analysis. The extent to which secretor status impacts HM composition is still being explored and may extend beyond HMOs. As such, stratification by secretor status in future analyses may be warranted to better understand this relationship and the subsequent impact on child growth. Furthermore, Larsson et al. [82] were not able to examine the relationships between HMO composition and infant growth in non-secretor mothers because of the small sample size. Although nonsecretors only represent less than half of the world population [120], limiting research findings to only combined and secretor milk impacts the generalizability of HM composition research. Consequently, a greater emphasis needs to be placed on recruiting an adequate number of nonsecretor mothers to conduct sufficient analyses and make recommendations for this portion of the population.

All studies used the presence of 2’FL as an indicator of secretor status as a phenotypic indicator of secretor status. Although identifying 2’FL in HM is feasible and the required technology is becoming more accessible through point-of-care testing [121], there remain many scenarios where genetic information is used to determine secretor status. In these instances, there may be added value to distinguishing between heterozygous and homozygous secretors because differences may exist for some HMOs between heterozygous and homozygous secretors. Finally, findings from this review indicate that infant secretor status is rarely considered when examining HM composition and child growth. Only 1 study included in this review [98] accounted for infant secretor status and there is a general paucity of research examining the role of infant secretor status in child health and developmental outcomes. Considerations of both maternal and infant physiology (including secretor status) and the interplay with HM as a triadic relationship will provide better insight toward recommended practices and therapeutic concepts related to HM composition [120].

### Moving research forward: the need to study HM from a systems perspective

Although the full impact of cytokines in HM is yet to be fully understood, it is believed that cytokines mediate infant immune responses through interactions with epithelial receptors [122]. In addition, cytokines are believed to act in concert with other cytokines, rather than in isolation. Similarly, it is believed that HMOs act in combination with each other [122] as well as individually. Furthermore, different types of bioactive constituents (for example, HMOs and cytokines) might also interact. In addition, some HM constituents are consumed or modified by the infant’s gut microbiota [123]. This underscores the importance of examining HM bioactive components as a network of interactions and pathways, from an ecological or biological systems perspective to develop a more comprehensive understanding of how HM components interact with each other to inform growth and subsequent health outcomes [124,125]. Yet very few studies took this approach. Jiang et al. [75] examined the combined role that the proteome, lipidome, and HMO composition in HM has on infant growth using PCA. Only 2 studies attempted integrated analyses of different HMOs [48,76] while 2 other studies performed an untargeted metabolomic analysis [74,102]. With the exception of Jiang et al. [75], Alderete et al. [48] who included HM insulin into their HMO model and Jorgensen et al. [76] who explored HMOs and bioactive proteins in concert with each other, no studies integrated different classes of milk bioactives. Conducting these comprehensive analyses and looking at HM from a systems perspective requires investment from scientists and funders to develop assays to optimize milk volume, to collect samples using consistent and validated strategies, and to include large sample sizes from diverse populations across the globe. Echoing Reyes et al. [6], there is a need for large high-quality studies to better understand how HM components work independently and together to influence infant growth and health outcomes.

### Strengths and limitations

Across 3 studies [6,7], we have comprehensively synthesized the available evidence for HM components and child anthropometric measures in the first 2 y. The main limitation of our review was the inability to conduct meta-analyses because of incomplete reporting practices and variability in sampling times and outcome measurements. Although meta-analyses are considered to be the gold standard, using SWiM [25] as a reporting guideline is an acceptable synthesis method in the absence of meta-analyses and allows us to synthesize and present study findings that would otherwise be left unreported. Individual studies included in this review also had limitations; only 5 of 75 achieved a high-quality score. Most did not adequately control for confounding (maternal BMI, birth anthropometrics, time postpartum, and HM exclusivity) and many did not provide results for all examined outcomes. We assumed that no relationship existed when an outcome was not reported, which limited our understanding and ability to incorporate these findings into the overall review. Finally, most studies measured bioactive concentrations, rather than calculated intakes. Assessing bioactives in milk using concentrations from 1 feed does not appropriately indicate the overall intake of these components by an infant over time and can lead to measurement bias.

## Conclusions

Bioactive components in HM are increasingly being examined as important influencers of child growth. Although our findings in this review were largely inconsistent, general trends were observed for HM adiponectin and leptin, demonstrating inverse relationships with infant growth. In addition, in secretor mothers, the HMO LNnT consistently demonstrated inverse trends with child growth outcomes, whereas 2’FL demonstrated positive associations. No immunomodulatory components demonstrated consistent directional relationships with infant growth outcomes.

Our synthesis of this literature was limited by methodological issues with milk collection strategies, timing of milk collection, inconsistent anthropometric measures that were not aligned with WHO guidelines, and insufficient reporting of findings. Moving forward, HM researchers need to be cognizant of aligning research strategies with clinically meaningful growth outcomes, developing HM sampling strategies that reflect the temporal fluctuations in composition, capturing daily HM intake, and applying analysis strategies that acknowledge and investigate HM as a complex biological system.

## Author contributions

The authors’ responsibilities were as follows—M(M)B, SMR, MBA: designed the research; M(M)B, SMR, MBA, NR: oversaw the research; SMR, M(M)B, JMM, DC, MG, RR, KKS, SM, PP, CM, LL: conducted the systematic review; M(M)B, SMR, JG, LL, LB, MBA: synthesized the data; M(M)B, MBA: wrote the paper and have primary responsibility for the final content; M(M)B, SMR, JG, JMM, MG, DTG, FJ, PK, LHA, LB, DH, KGE: provided critical review and contribution to the manuscript; and all authors: read and approved the final manuscript.

## Funding

This review was undertaken as part of the International Milk Composition (IMiC) Consortium, funded by the Bill & Melinda Gates Foundation (INV-001734).

## Data availability

Data described in the manuscript, code book, and analytic code will be made available upon request pending application and approval by study authors.

## Declaration of interests

The authors declare the following financial interests/personal relationships which may be considered as potential competing interests:

This research was funded as part of the International Milk Composition (IMiC) Consortium, funded by the Bill & Melinda Gates Foundation (INV-001734).

## Conflict of interest

M(M)B, SMR, and MBA have contributed to online courses on breast milk and the infant microbiome produced by Microbiome Courses. SMR has also served as the scientific advisor for SimpliFed and as a consultant for TraverseScience®. She is a current employee of Prolacta Bioscience®; her contribution to this review occurred prior to this employment. JMM has received support from the Bill & Melinda Gates Foundation and serves on the Council on Research for the American Academy of Nutrition and Dietetics. DC is supported by a Canadian Nurses Foundation Scholarship. DTG is funded by an unrestricted research grant from Medela AG. She is also currently funded by Telethon Child Health Grants and the Australian National Health and Medical Research Council. LHA has research grants from the Bill & Melinda Gates Foundation. MBA is supported by a Canada Research Chair and is a CIFAR Fellow in the Humans and the Microbiome Program; she has consulted for DSM and is a scientific advisor to TinyHealth. LB is UC San Diego Chair of Collaborative Human Milk Research endowed by the Family Larsson-Rosenquist Foundation and also receives support from the US National Institutes of Health and The Bill & Melinda Gates Foundation. AID, MG, RR, KKS, SM, PPP, CM, FJ, PK, DH, and KGE have no conflicts of interest.
