# Supplementary material for: Human Milk Bioactive Components and Child Growth and Body Composition in the First 2 Years: A Systematic Review
Source: Adv Nutr. 2023 Oct 4;15(1):100127. doi: 10.1016/j.advnut.2023.09.015 (PMC10831900; doi:10.1016/j.advnut.2023.09.015)
Supplement: Multimedia component 1 [file mmc1.pdf]

Table S1. Study quality assessment criteria: human milk bioactives and child growth.
Total score <7=Low, 7-13=Moderate, >13-17=High.

Item	Description	Points
**Human Milk Exposure Assessment**		<3=Low, 3-6=Moderate, >6-8=High
1	**Sampling strategy/ handling protocol**	
	**Hormones & Immunomodulators**	
1.1.0	Milk collection protocol	1 - Full breast expression standardized for time of day
		0 - Any other collection method
1.1.1	Stage of lactation	1 - Time postpartum standardized across participants
		0 - Time postpartum not standardized
1.1.2	Handling protocol	1 - Analyzed fresh or within 2 days of sampling
		0 - No precautions taken
	**Human Milk Olligosaccharides**	
1.2.0	Milk collection protocol	1.5 - Full breast expression standardized for time of day
		0 - Any other collection method
1.2.1	Stage of lactation	1.5 - Time postpartum standardized across participants
		0 - Time postpartum not standardized
2	**Sample preparation**	
2.1.0	Reproducibility	1 - Reproducible using information provided in current paper OR direct reference
		0 - Not reproducible using informaiton provided in current paper OR direct reference
3	**Analytical Method**	
3.1.0	Validation / quality control	2 - Method validated in human milk, including recovery data and inter-/intra-assay variation*** in current paper OR direct reference
		1 - Particial validation (e.g., precision or accuracy data reported, but not both in current paper OR direct reference
		0.5 - Commercial assays or commonly used methods but no validation data specific to human milk in current paper OR direct reference
4	**Longitudinal sampling**	
4.1.0	Longitudinal sampling of human milk	1 - Milk samples collected longitudinally (e.g., 1, 3, and 6 mo)
		0 - Milk samples NOT collected longitudinally
5	**Human milk volume intake**	
5.1.0	Infant intake of human milk	1 - Volume of human milk infants consumed measured or estimated
		0 - Volume not considered
**Confounders Considered**		<3=Low, 3-4=Moderate, >4-5=High
6	**Infant diet**	
6.1.0	Breastfeeding exclusivity	2 - All infants exclusively breastfed at time of human milk collection
		up to 1.5 - If NOT all infants exclusively breastfed:
		0.5 - Reporting breastfeeding status
		0.5 - Adjusting for it in analyses
		0.5 - Reports number of proportion of human milk vs. other milks
		0 - Breastfeeding exclusivity of infants unclear
7	**Birth anthropometrics**	
7.1.0	Birth anthropometrics	1 - Any birth anthropometrics accounted for in study design or analyses
		0 - No birth anthropometrics accounted for
8	**Baseline characteristics**	
8.1.0	Maternal characteristics reported	0.5 - Reports maternal age parity, BMI, ethnicity, time postpartum, others relevant to study
		0 - Does not report maternal characteristics
8.1.1	Infant characteristics reported	0.5 - Reports infants age [or time postpartum] and sex
		0 - Does not report infant characteristics
8.1.2	Maternal characteristics accounted for	1 - Accounted for in analyses or study design
		0 - Not accounted for
8.1.3	Infant characteristics accounted for in analyses	1 - Accounted for in analyses or study design
		0 - Not accounted for
**Infant Anthropometric Outcomes**		<2=Low, 2-3=Moderate, >3-4=High
9	**Infant anthropometrics**	
9.1.0	Source of anthropometric measurements	1 - Trained staff
		0.5 - Collected from clinical records
		0 - Self-reported
9.1.1	Technical replicates performed?	1 - Yes
		0 - No
9.1.2	Longitudinal measurements?	1 - Infant anthropometrics measured multiple times over time (e.g., 1, 3, and 6 mo)
		0 - Anthropometrics NOT measured longitudinally
9.1.3	Timing of measurements across infants	1 - All infants measured at same age (± 1 week)
		2 - All infants measured at similar age (± 1 month)
		0 - Timing of measurements not standardized across infants

***(e.g., data accuracy and precision)

Table S2. Study quality* assessment results: human milk bioactives and child growth.

Study Details	Human Milk Exposure Assessment					Confounders Considered				Infant Anthropotmetric Outcomes				Summary & Total Scores**			
	1	2	3	4	5	6	7	8		9							
**Authors** (Alphabetical) **Country, Publication Year (Setting)**	**Sampling strategy/ handling protocol**	**Sample preparation**	**Analytical method**	**Longitudinal sampling**	**Human milk volume intake**	**Infant diet**	**Birth anthropometrics**	**Baseline characteristics reported**	**Baseline characteristics accounted for**	**Source of measurements**	**Techinical replicates**	**Longitudinal measurements**	**Standardization of timing of measurements**	**Human Milk Exposure Assessment**	**Confounders Considered**	**Infant Anthropometric Outcomes**	**TOTAL**
Aksan et al. Turkey, 2021 (UMIC)	2.0	0.0	2.0	0.0	0.0	0.0	1.0	1.0	0.0	1.0	0.0	1.0	0.5	4.0	2.0	2.5	**8.5**
Alderete et al. USA, 2015 (HIC)	3.0	1.0	1.0	1.0	0.0	2.0	0.5	0.8	0.8	1.0	1.0	1.0	1.0	6.0	4.0	4.0	**14.0**
Alsharnoubi et al. Egypt, 2019 (LMIC)	0.0	0.5	0.5	0.0	0.0	0.0	0.5	0.6	0.8	0.0	0.0	0.0	0.0	1.0	1.9	0.0	**2.9**
Anderson et al. Phillipines, 2016 (LMIC)	1.0	1.0	2.0	0.0	0.0	0.0	0.0	1.0	1.0	1.0	0.0	0.0	0.0	4.0	2.0	1.0	**7.0**
Andreas et al. England, 2016 (HIC)	2.0	1.0	2.0	1.0	0.0	0.0	0.5	1.0	0.5	0.5	0.0	1.0	1.0	6.0	2.0	2.5	**10.5**
Baroncelli et al. Malawi, 2018 (LIC)	0.5	0.5	0.5	0.0	0.0	2.0	0.3	0.8	0.5	0.5	0.0	0.5	1.0	1.5	3.5	2.0	**7.0**
Binia et al. Europe, 2021 (HIC)	3.0	1.0	2.0	1.0	0.0	0.0	1.0	0.9	1.0	0.5	0.5	1.0	1.0	7.0	2.9	3.0	**12.9**
Bronsky et al. Czech Republic, 2011 (HIC)	2.0	1.0	0.5	1.0	0.0	0.3	0.0	1.0	0.0	0.8	0.0	1.0	0.3	4.5	1.3	2.0	**7.8**
Brunn et al. Denmark, 2018 (HIC)	2.5	1.0	0.8	0.0	0.0	1.3	1.0	0.9	1.0	0.5	0.0	1.0	0.8	4.3	4.1	2.3	**10.6**
Brunner et al. Germany, 2015 (HIC)	2.0	1.0	0.5	1.0	0.0	1.0	0.5	1.0	1.0	0.4	1.0	1.0	1.0	4.5	3.5	3.4	**11.4**
Campbell et al. The Gambia, 2006 (LIC)	1.0	1.0	0.8	1.0	0.0	0.3	0.5	0.5	0.5	0.5	0.5	1.0	1.0	3.8	1.8	3.0	**8.5**
Cannon et al. Australia, 2015 (HIC)	2.0	1.0	2.0	0.0	1.0	1.5	0.5	1.0	0.5	0.5	0.0	0.0	0.0	6.0	3.5	0.5	**10.0**
Cesur et al. Turkey, 2012 (UMIC)	2.0	1.0	0.3	1.0	0.0	1.5	0.5	0.8	0.5	1.0	0.5	1.0	0.8	4.3	3.3	3.3	**10.8**
Chan et al. Canada, 2018 (HIC)	1.8	0.5	0.5	0.0	0.0	1.0	0.5	1.0	1.0	1.0	0.5	1.0	1.0	2.8	3.5	3.5	**9.8**
Cheema et al. Australia, 2021 (HIC)	2.0	1.0	2.0	0.0	1.0	2.0	1.0	1.0	1.0	1.0	0.0	0.0	1.0	6.0	5.0	2.0	**13.0**
Cheema et al. Australia, 2022 (HIC)	1.0	1.0	2.0	0.0	1.0	2.0	1.0	0.5	0.0	1.0	0.0	0.5	1.0	5.0	3.5	2.5	**11.0**
Davis et al. The Gambia, 2017 (LIC)	3.0	1.0	1.3	1.0	0.0	0.0	0.0	0.6	0.0	1.0	0.5	1.0	1.0	6.3	0.6	3.5	**10.4**
Doneray et al. Turkey, 2009 (UMIC)	1.0	0.5	0.8	1.0	0.0	2.0	0.5	1.0	0.5	1.0	0.0	1.0	1.0	3.3	4.0	3.0	**10.3**
Dundar et al. Turkey, 2005 (UMIC)	2.0	0.5	2.0	1.0	0.0	2.0	0.5	1.0	0.5	1.0	0.0	1.0	1.0	5.5	4.0	3.0	**12.5**
Durilova et al. Czech Republic, 2010 (HIC)	0.8	1.0	0.5	0.0	0.0	0.0	0.5	0.8	0.3	0.0	0.0	0.0	0.0	2.3	1.5	0.0	**3.8**
Ellsworth et al. USA, 2020 (HIC)	1.5	1.0	0.5	0.0	0.0	0.5	0.5	0.9	0.8	0.6	0.0	1.0	0.8	3.0	2.6	2.4	**8.0**
Enstad et al. USA, 2019 (HIC)	1.0	1.0	1.0	1.0	0.0	1.0	1.0	0.8	1.0	1.0	0.0	1.0	0.8	4.0	3.8	2.8	**10.5**
Fatima et al. Pakistan, 2018 (LMIC)	1.3	1.0	1.3	1.0	0.0	0.0	0.5	1.0	0.8	0.5	0.0	0.5	1.0	4.5	2.3	2.0	**8.8**
Fatima et al. Pakistan, 2022 (LMIC)	2.0	1.0	0.5	1.0	0.0	2.0	1.0	1.0	1.0	0.5	0.0	1.0	0.5	4.5	5.0	2.0	**11.5**
Fields et al. USA, 2017 (HIC)	2.0	1.0	0.5	0.5	0.0	2.0	0.5	0.9	0.8	1.0	1.0	1.0	1.0	4.0	4.1	4.0	**12.1**
Galante et al. Finland, 2020 (HIC)	1.0	1.5	0.0	0.0	0.0	1.0	1.0	1.0	0.8	0.0	1.0	0.8	4.5	3.0	2.5	4.5	**10.0**
Goran et al. USA, 2017 (HIC)	2.5	1.0	0.5	1.0	0.0	2.0	0.0	0.9	1.0	1.0	0.5	1.0	1.0	5.0	3.9	3.5	**12.4**
Gridneva et al. Australia, 2018 (HIC)	2.0	1.0	2.0	1.0	1.0	2.0	0.0	1.0	0.0	1.0	0.0	1.0	0.8	7.0	3.0	2.8	**12.8**
Gridneva et al. Australia, 2020 (HIC)	1.5	0.0	1.5	1.0	1.0	1.0	0.0	1.0	0.0	1.0	0.0	1.0	0.8	5.0	2.0	2.8	**9.8**
Gridneva et al. Australia. 2021 (HIC)	2.0	0.5	2.0	1.0	1.0	2.0	0.0	1.0	1.0	1.0	1.0	1.0	1.0	6.5	4.0	4.0	**14.5**
Guler et al. Turkey, 2021 (UMIC)	2.0	1.0	2.0	0.0	0.0	0.0	1.0	1.0	0.0	1.0	1.0	1.0	0.5	5.0	2.0	3.5	**10.5**
Hollanders et al. Netherlands, 2019 (HIC)	2.0	1.0	2.0	0.0	0.0	1.5	0.5	0.9	1.0	1.0	0.0	1.0	0.8	5.0	3.9	2.8	**11.6**

Table S2. Study quality* assessment results: human milk bioactives and child growth.

Study Details	Human Milk Exposure Assessment					Confounders Considered				Infant Anthropotmetric Outcomes				Summary & Total Scores**			
	1	2	3	4	5	6	7	8		9							
**Authors** (Alphabetical) Country, Publication Year (Setting)	Sampling strategy/ handling protocol	Sample preparation	Analytical method	Longitudinal sampling	Human milk volume intake	Infant diet	Birth anthropometrics	Baseline characteristics reported	Baseline characteristics accounted for	Source of measurements	Techinical replicates	Longitudinal measurements	Standardization of timing of measurements	Human Milk Exposure Assessment	Confounders Considered	Infant Anthropometric Outcomes	TOTAL
Isganaitis et al. USA, 2019 (HIC)	2.5	1.0	2.0	1.0	0.0	2.0	1.0	0.9	1.0	1.0	0.0	1.0	1.0	6.5	4.9	3.0	**14.4**
Jiang et al. China, 2021 (UMIC)	1.0	1.0	2.0	0.0	0.0	0.0	1.0	1.0	1.0	1.0	0.0	0.0	0.0	4.0	3.0	1.0	**8.0**
Jorgensen et al. Malawi, 2020 (LIC)	1.5	1.0	1.3	0.0	0.0	0.0	0.5	0.3	0.9	1.0	1.0	1.0	1.0	3.8	1.6	4.0	**9.4**
Khaghani et al. Iran, 2006 (UMIC)	2.0	0.0	0.8	1.0	0.0	0.0	0.0	0.3	0.3	0.5	0.0	1.0	0.8	3.8	0.5	2.3	**6.5**
Khodabakhshi et al. Iran, 2015 (LMIC)	1.0	0.5	0.5	0.0	0.0	2.0	0.5	0.8	0.5	1.0	0.0	1.0	1.0	2.0	3.8	3.0	**8.8**
Kon et al. Russia, 2014 (HIC)	1.5	0.5	1.3	1.0	1.0	0.5	0.0	0.0	0.0	1.0	0.0	1.0	1.0	5.3	0.5	3.0	**8.8**
Kuziez et al. Philippines, 2020 (LMIC)	1.0	1.0	1.5	0.0	0.0	0.0	0.5	0.8	1.0	0.8	0.0	0.5	0.0	3.5	2.3	1.5	7.0
Lagstrom et al. Finland, 2020 (HIC)	2.3	1.0	0.3	0.0	0.0	0.3	1.0	0.9	0.8	0.8	0.0	0.5	1.0	3.5	2.9	2.3	**8.6**
Larson-Meyer et al. United States, 2020 (HIC)	2.5	1.0	2.0	1.0	0.0	0.3	0.5	0.8	0.0	1.0	0.0	1.0	0.8	6.5	1.5	2.8	**10.8**
Larsson et al. Denmark, 2018 (HIC)	2.3	1.0	0.5	0.5	1.0	0.5	0.0	0.9	0.3	1.0	0.5	1.0	0.5	5.3	1.6	3.0	**9.9**
Larsson et al. Denmark, 2018 (HIC)	2.0	0.0	0.7	1.0	1.0	0.5	0.0	0.9	0.1	1.0	1.0	1.0	0.5	4.7	4.5	3.5	**9.7**
Leghi et al. Australia, 2021 (HIC)	1.0	1.0	2.0	1.0	1.0	2.0	1.0	1.0	1.0	1.0	1.0	1.0	0.0	6.0	5.0	3.0	**14.0**
Logan et al. Germany, 2019 (HIC)	2.0	1.0	1.0	0.5	0.0	0.5	0.5	1.0	1.0	0.8	0.0	1.0	0.5	4.5	3.0	2.3	**9.8**
Liu et al. China, 2021 (UMIC)	0.0	0.0	0.5	1.0	0.0	2.0	0.0	1.0	1.0	1.0	0.0	1.0	0.0	1.5	4.0	2.0	**7.5**
Menzel et al. Germany, 2021 (HIC)	2.0	0.0	2.0	0.0	0.0	0.0	1.0	1.0	1.0	1.0	1.0	1.0	0.0	4.0	3.0	3.0	**10.0**
Messripour et al. Iran, 2002 (LMIC)	0.5	1.0	0.5	1.0	0.0	2.0	0.0	0.5	0.5	1.0	0.0	1.0	1.0	3.0	3.0	3.0	**9.0**
Miralles et al. Spain, 2006 (HIC)	1.5	1.0	0.8	1.0	0.0	2.0	0.5	0.8	0.5	0.5	0.0	1.0	1.0	4.3	3.8	2.5	**10.5**
Mohamad et al. Malaysia. 2018 (UMIC)	2.0	1.0	2.0	1.0	0.0	0.8	0.3	1.0	1.0	1.0	0.0	1.0	1.0	6.0	3.2	3.0	**12.2**
Nikniaz et al. Iran, 2013 (LMIC)	2.0	1.0	0.8	0.0	0.0	2.0	1.0	0.8	1.0	0.5	0.0	0.5	1.0	3.8	4.8	2.0	**10.5**
Nuss et al. USA, 2019 (HIC)	1.0	1.0	0.5	0.0	0.0	0.5	0.0	0.9	0.5	1.0	1.0	0.0	0.8	2.5	1.9	2.8	**7.1**
Ortiz-Andrellucchi et al. Spain,2008 (HIC)	1.8	1.0	1.3	1.0	0.0	0.0	0.5	1.0	0.5	1.0	0.0	1.0	1.0	5.0	2.0	3.0	**10.0**
Pundir et al. Australia, 2020 (HIC)	1.5	1.0	0.8	1.0	0.5	2.0	0.5	1.0	0.5	0.8	0.0	1.0	1.0	4.8	4.0	2.8	**11.5**
Quinn et al. Nepal, 2017 (LMIC)	2.0	1.0	1.5	0.0	1.0	0.5	0.0	0.8	0.8	1.0	1.0	0.0	0.0	5.5	2.0	2.0	**9.5**
Saben et al. USA, 2021 (HIC)	2.5	1.0	2.0	0.0	1.0	1.3	1.0	0.8	1.0	1.0	0.0	1.0	0.8	6.5	4.0	2.8	**13.3**
Saso et al. The Gambia, 2018 (LIC)	2.0	1.0	1.0	1.0	0.0	0.0	11.0	1.0	1.0	1.0	0.0	1.0	0.5	5.0	3.0	2.5	**10.5**
Savino et al. Italy, 2012 (HIC)	1.5	1.0	1.5	0.0	0.0	1.0	0.5	0.8	0.5	1.0	0.0	0.0	1.0	4.0	2.8	2.0	**8.8**
Schueler et al. USA, 2013 (HIC)	2.5	1.0	1.3	0.0	0.0	1.5	0.5	0.8	0.5	1.0	0.0	1.0	1.0	4.8	3.3	3.0	**11.0**
Schuster et al. Germany, 2011 (HIC)	1.5	1.0	2.0	1.0	0.0	0.0	0.0	1.0	0.5	0.5	0.0	1.0	1.0	5.5	1.5	2.5	**9.5**
Sims et al. USA, 2020 (HIC)	2.0	0.5	0.5	1.0	1.0	0.8	0.5	0.9	0.5	1.0	0.0	1.0	1.0	5.0	2.6	3.0	**10.6**
Sprenger et al. Singapore, 2017 (HIC)	3.0	1.0	2.0	1.0	0.0	0.5	0.5	0.8	1.0	1.0	0.0	1.0	0.8	7.0	2.8	2.8	**12.5**
Tonon et al. Brazil, 2019 (UMIC)	0.8	1.0	1.5	0.0	0.0	2.0	0.5	0.9	0.8	0.8	0.0	0.0	0.4	3.3	4.1	1.1	**8.5**
Ucar et al. Turkey, 2000 (UMIC)	1.0	0.5	0.5	0.0	0.0	0.0	0.0	0.9	0.0	0.5	0.0	0.0	1.0	2.0	0.9	1.5	**4.4**

Table S2. Study quality* assessment results: human milk bioactives and child growth.

Study Details	Human Milk Exposure Assessment					Confounders Considered				Infant Anthropotmetric Outcomes				Summary & Total Scores**			
	1	2	3	4	5	6	7	8		9							
**Authors** (Alphabetical) **Country, Publication Year (Setting)**	**Sampling strategy/ handling protocol**	**Sample preparation**	**Analytical method**	**Longitudinal sampling**	**Human milk volume intake**	**Infant diet**	**Birth anthropometrics**	**Baseline characteristics reported**	**Baseline characteristics accounted for**	**Source of measurements**	**Techinical replicates**	**Longitudinal measurements**	**Standardization of timing of measurements**	**Human Milk Exposure Assessment**	**Confounders Considered**	**Infant Anthropometric Outcomes**	**TOTAL**
Uysal et al. Turkey, 2002 (UMIC)	3.0	1.0	1.3	0.0	0.0	1.5	0.5	0.9	0.5	1.0	0.0	0.0	1.0	5.3	3.4	2.0	**10.6**
Van Rossem et al. Netherlands, 2019 (HIC)	0.0	1.0	1.5	0.0	0.0	0.8	1.0	0.6	1.0	0.3	0.0	1.0	0.8	2.5	3.4	2.0	**7.9**
Wang et al. China, 2020 (UMIC)	3.0	1.0	1.5	1.0	0.0	0.0	0.5	1.0	0.8	1.0	0.0	1.0	1.0	6.5	2.3	3.0	**11.8**
Weyermann et al. Germany, 2007 (HIC)	1.8	1.0	1.5	0.0	0.0	1.0	1.0	0.6	0.7	0.6	0.0	1.0	0.5	4.3	3.4	2.1	**9.8**
Wolfs et al. USA, 2021 (HIC)	1.0	1.0	0.5	1.0	0.0	0.0	1.0	1.0	1.0	1.0	1.0	1.0	1.0	3.5	3.0	4.0	**10.5**
Woo et al. USA and Mexico, 2012 (HIC and UMIC)	1.3	0.8	1.3	1.0	0.0	1.5	1.0	0.5	0.0	0.5	0.0	1.0	0.3	4.3	3.0	1.8	**9.0**
Wren-Atilola et al. Guatemala, 2021 (LMIC)	2.0	0.0	2.0	1.0	0.0	1.5	0.0	1.0	1.0	1.0	1.0	1.0	0.0	5.0	3.5	3.0	**11.5**
Wu et al. China, 2021 (UMIC)	2.0	1.0	0.8	1.0	0.0	1.0	0.5	0.4	0.0	1.0	0.5	1.0	0.5	4.8	1.9	3.0	**9.6**
Yilmaz et al. Turkey, 2021 (UMIC)	1.3	0.5	0.8	1.0	0.0	1.0	0.5	0.8	0.0	1.0	0.0	1.0	1.0	3.5	2.3	3.0	**8.8**
Yis et al. Turkey, 2010 (UMIC)	1.8	1.0	1.3	0.0	0.3	1.5	1.0	0.5	0.3	0.8	0.0	1.0	0.5	4.3	3.3	2.3	**9.8**
Yu et al. Beijing, 2018 (UMIC)	2.0	1.0	1.5	1.0	0.0	2.0	0.0	0.8	0.0	1.0	1.0	1.0	1.0	5.5	2.8	4.0	**12.3**
Zamanillo et al. Spain, 2019 (HIC)	2.0	1.0	2.0	1.0	0.0	0.5	0.0	1.0	0.8	0.5	0.0	1.0	0.8	6.0	2.3	2.3	**10.5**

*See Table S1 for 9 categories of quality assessment criteria. **Red = low, Yellow = moderate, Green = high. See Methods for details.
World Bank Income Setting: LMIC, low middle income country; UMIC, upper middle income country; HIC, high income country.

Table S3. Characteristics and results of included studies reporting on human milk hormones and infant anthropometrics - organized by component.

Authors, country, publication year (income setting)	Design and participants	Timing of milk sampling	Timing of infant anthropometrics	Estimated intake or HM concentration*	Anthropometric outcome measures and standards	Associations**	Major confounders considered
**Adiponectin (19 studies, 19 articles - 3,479 dyads)**							
Anderson et al. Philippines, 2016 (LMIC)	Cohort (132 enrolled, 117 analysed)	10 days to 4 years (only m	10 days to 4 years Only in	Concentration in HM	WAZ, BMI Z-score	(+) Association between milk adiponectin and infant WAZ<br>(+) Association between milk adiponectin and infant BMI Z	Maternal BMI, infant age, infant nursing frequency
Bronsky et al. Czech Republic, 2011 (HIC)	Cohort (72 enrolled & analyzed)	Colostrum, month 1, month 3, month 6, and 12 months postpartum	Birth, month 1, month 3, month 6, and 12 months	Concentration in HM	Body weight, length	(+) Association between weight gain during year 1 and adiponectin breast milk concentration at month 6 (r = 0.2774, p = 0.0488)	None reported
Brunner et al. Germany, 2015 (HIC)	RCT (208 enrolled), 152 analysed at 6 weeks, 120 analysed at 4 months.	6 weeks postpartum, 4 months postpartum	Birth, 6 weeks, 4 months,	Concentration in HM	Weight, BMI, sum 4 SFT, body fat percentage, fat mass (g), lean body mass (g), weight gain (6 weeks - 4 months postpartum)	(-) Association between milk adiponectin and infant lean body mass at 4 months<br>(+) Association between milk adiponectin (unadjusted) and infant weight gain and fat mass up to 2 years of life, (adjusted - upto 1 year of life)	Maternal pre-pregancy BMI, gestational weight gain, pregnancy duration, infant sex, infant ponderal index at birth, mode of infant feeding at 4 months postpartum
Cesur et al. Turkey, 2012 (UMIC)	Longitudinal (25 enrolled, 19 analyzed)	Month 1 and 4 months postpartum	Month 1 and 4 months pos	Concentration in HM	Weight, weight gain, BMI	(No) Association between adiponectin levels in breast milk and growth parameters of infants	None reported
Chan et al. Canada, 2018 (HIC)	Longitudinal (cohort)<br><br>1094 analyzed	3-4 months postpartum	4 months, 1 year	Concentration in HM	Weight, length (WHO Child Growth Standards)	(No) Association between milk adiponectin and infant body composition	Prepregnancy maternal BMI, total breastfeeding duration, ethnicity, parity, diabetes, smoking, breastfeeding exclusivity, lactation stage
Galante et al. Finland, 2020 (HIC)	Cohort (501 mothers and 507 children enrolled)	2.6 +/- 0.4 months postpartum	13 months, 2, 3, 5 years	Concentration in HM	Weight, weight gain, BMI z-scores (Finnish population references)	(No) Associations for adiponectin reported (assumed no association)	Maternal pre-pregnancy BMI, infant sex, breastfeeding duration, intro of solid foods, infant birthweight
Gridneva et al., Australia. 2018 (HIC)<br><br>Gridneva et al., Australia. 2021 (HIC)	Cohort. 20 mother-infant dyads were enrolled	2, 5, 9, 12 months	2, 5, 9, 12 months	Estimated intake and concentration	Infant length, weight, BMI, head circumference, infant body composition, infant abdominal adiposity<br><br>(WHO standards)	(-) Association between CDI adiponectin and infant lean body mass.<br>(+) Association between CDI adiponectin and adiposity<br><br>(no other significant associations)	None reported
Khodabakhshi et al. Iran, 2015 (LMIC)	Cross-sectional (80 enrolled & analyzed)	Unclear	2, 4, 6 months	Concentration in HM	Weight, height	(-) Association between adiponectin and 2nd month weight for normal weight infants	None reported
Kon et al. Russia, 2014 (HIC)	Longitudinal (103 enrolled, 99 analyzed)	1, 2, 3 months postpartum	1, 2, 3 months postpartum	Concentration in HM	Weight gain	(No) Association between infant weight gain status and adiponectin	None reported
Larsson et al. Denmark, 2018 (HIC)	Longitudinal (59 enrolled, 30 analyzed)	First visit: 5-6.5 months old; Second visit: 9 months (+/- 2 weeks); Third visit for HW-group only: 18 months +/- 4 weeks	First visit: 5-6.5 months old;<br>Second visit: 9 months (+/- 2 weeks);<br>Third visit for HW-group only: 18 months +/- 4 weeks	Estimated intake and concentration in HM but associations determined using concentrations in milk	Weight, recumbent length, mid-upper-arm circumference, head circumference, lower leg circumference, recumbent waist and thorax circumference, triceps and subscapular skinfold thickness, WAZ, LAZ, BAZ, triceps skinfold for age z-score (TSFZ), subscapular skinfold-for-age z-score (SSFZ)	(No) Association between milk concentrations of adiponectin and infant's anthropometry or change in z-scores from birth to the 5-month visit	Maternal fasting time, infant sex
Leghi et al. Australia, 2021 (HIC)	Open label crossover design (18 enrolled and analyzed)	Week 1 (baseline), week 2 and week 3 of intervention (Infant age not standardized)	Week 1 (baseline), week 2 and week 3 of intervention (Infant age not standardized)	Estimated intake and concentration in HM	Weight, length, head circumference, BMI, WFLZ, WFAZ, LFAZ,<br><br>(WHO standards)	(NO) outcomes reported for Hormones (Assumed no relationship)	Controlled for maternal BMI
Mohamad et al. Malaysia. 2018. UMIC	Cohort. 155 enrolled	0 and 2 months postpartum	Birth, 2, 6, 12 months	Concentration in HM	Infant body weight, infant BMI-for-age Z-score (BAZ)	(-) Association between breastmilk adiponectin and infant BAZ, infant body weight and abdominal circumference at 2 months .	Gestational weight gain,gestational age, maternal age,pre-pregnancy BMI,Infant sex, breastfeeding patterns, breastfeeding exclusivisity(exclusive, partial or no breastfeeding)
Quinn et al., Nepal, 2017 (LMIC)	Cross-sectional. (50 women from Nubri and 66 women from Kathmandu)	NV=11.02 months +/- 7.66; K=11.70 mos +/- 8.44	NV=11.02 months +/-7.66;	Concentration in HM	Weight, length, head circumfe	(No) Association between adiponectin and infant WAZ (Nubri)<br>(-) Association between adiponectin and infant WAZ (Katmandu)	Infant age, transferred milk volume, infant sex, birth order, nursing frequency and, in the Nubri Valley sub-set, altitude of residence.

Table S3. Characteristics and results of included studies reporting on human milk hormones and infant anthropometrics - organized by component.

Authors, country, publication year (income setting)	Design and participants	Timing of milk sampling	Timing of infant anthropometrics	Estimated intake or HM concentration*	Anthropometric outcome measures and standards	Associations**	Major confounders considered
Van Rossem et al. The Netherlands, 2019 (HIC)	Longitudinal<br>251 enrolled, 223 analyzed	2 - 25 weeks	3 months,<br>1, 2, 3, 4, 5, 6, 7, 8, 11/12, 14, and 16/17 years	Concentration in HM	BMI z-score,<br>Weight-for-age z-score,<br>Length/height-for-age z-score	(-) Association between adiponectin and BMI z-score and WFA z-score at age of 3 months<br>(-) Association between adiponectin and weight gain between birth and 3 months<br>(-) Association between adiponectin and weight and height between age of 1 and 17 years	Maternal age, maternal pre-pregnancy BMI, gestational weight gain, age of infant at breastmilk measurement, sex, age at weight measurement, presence of siblings of infant, birthweight
Weyermann et al. Germany, 2007 (HIC)	Cohort.<br><br>1066 enrolled<br>767 analyzed	Range 33 and 71 days postpartum (mean 44 days; median 43 days; 10th-90th percentile 39–48 days)	6 weeks postpartum	Concentration in HM	Infant BMI, overweight risk (German reference population)	(+) Association between children who were breast-fed for at least 6 months and increased risk for overweight at the age of 2 with increasing breast milk adiponectin levels; this risk persisted after adjustment for covariates.	Age of mother (years), school education of mother (9 years; 10 years), nationality of mother (German; Turkish; other), BMI of mother at the beginning of pregnancy (kg/m2), smoking status of mother during follow-up (no; yes), and birthweight (grams). In addition to analyses in the overall sample, specific analyses were carried out according to duration of breast-feeding (6 months; 6 months).
Woo et al.<br>USA; Mexico, 2009 (HIC); (UMIC)	Longitudinal<br><br>USA<br>46 enrolled and 45 analyzed,<br><br>Mexico<br>206 enrolled and 277 analyzed	USA:<br>1,2,3,4,5 and 6 months postpartum<br><br>Mexico:<br>2 samples, anytime bewteen 1 week and 6 months postpartum	Monthly, up to 6 months postpartum	Concentration in HM	Weight, length, BMI, weight-for-age z score, length-for-age z-score, weight-for-length z-score<br>(WHO)	*In cross-sectional analysis:*<br>(-) Association between milk adiponectin and infant WA z-score at baseline and months 1 and 3<br>(-) Association between milk adiponectin and WFL z-score at baseline and months 1 and 3<br><br>*In longitudinal analysis:*<br>(-) Association between milk adiponectin and infant WA z-score<br>(-) Association between milk adiponectin and WFL z-score<br>(No) Association between milk adiponectin and infant length or LA z-score	For both cross-sectional and longtudinal analysis: cohort, sex, age in days<br>for cross-sectional analysis only: length (for WA z-score), weight (for LA z-score.<br>For longitudinal analysis only: month, month^2, infant birth weight
Yu et al.<br>Beijing, 2018 (UMIC)	Longitudinal<br><br>(121 enrolled, 96 analyzed for day 3 colostrum, 78 analyzed for day 42 mature milk, 61 analyzed for day 90 mature milk)	Colostrum on day 3 postpartum, mature milk on day 42 and 90	Days 3, 42, 90 postpartum	Concentration in HM	Weight, length, head circumference, weight-for-height gain	(-) Association between overall adiponectin concentration during first three months and infant weight-for-height in both GDM and healthy groups<br>(-) Association between adiponectin, insulin and head circumference during follow-up period (i.e. after day 3), but insulin association was insignificant after Bonferonni correction<br>(-) Association between day 90 adiponectin and weight-for-height in GDM group<br><br>(+) Association between day 90 adiponectin and weight-for-height in healthy group	Maternal gestational diabetes status
Zamanillo et al., Spain, 2019 (HIC)	Longitudinal<br><br>59 infant-mother dyads. (38 normal-weight and 21 overweight/obese (BMI ≥ 25)	30, 60, 90 days postpartum	30, 60, 90 days postpartum	Concentration in HM	Infant BMI	(No) Associations between HM analytes of interest and infant anthros.	Maternal BMI
**Leptin (34 studies, 35 articles - 5,857 dyads)**							
Andreas et al.<br>England, 2016 (HIC)	Cohort<br>(105 enrolled)	1 week and 3 months postpartum	1 week and 3 months postpartum	Concentration in HM	Weight, length, head circumference, weight change from birth to 7 days, weight change from 7 days to 3 months, weight change from birth to 3 months	(No) Association between hormone concentrations in foremilk samples collected at 1 week postpartum and infant anthros<br><br>NOT PREDICTIVE:<br>(No) Association between infant anthros and hormone concentrations in hindmilk samples collected at 3 months postpartum	None reported
Bronsky et al.<br>Czech Republic, 2011 (HIC)	Cohort<br>(72 enrolled & analyzed)	Colostrum, month 1, month 3, month 6, and 12 months postpartum	Birth, month 1, month 3, month 6, and 12 months	Concentration in HM	Body weight, length	(-) Association between leptin and body length at birth (r = -0.2666, p = 0.0450)	None reported
Brunner et al.<br>Germany, 2015 (HIC)	RCT<br>(208 enrolled), 152 analysed at 6 weeks, 120 analysed at 4 months.	6 weeks postpartum, 4 months postpartum	Birth, 6 weeks, 4 months,	Concentration in HM	Weight, BMI, sum 4 SFT, body fat percentage, fat mass (g), lean body mass (g), weight gain (6 weeks - 4 months postpartum)	(-) Association between milk leptin measured at 4 months and concurrent infant weight and lean body mass (adjusted)<br>(No) Association between milk leptin measured at 6 weeks and any infant growth and body composition until 2 years<br>(No) Association between milk leptin measured at 4 months and infant growth and body composition and followup later than 4 months	Maternal pre-pregancy BMI, gestational weight gain, pregnancy duration, infant sex, infant ponderal index at birth, mode of infant feeding at 4 months postpartum
Cannon et al.<br>Australia, 2015 (HIC)	Cohort<br>(19 enrolled)	NR, but assumed 3-21 weeks (same age as infant)	Presumed 3-21 weeks	Concentration in HM	Weight	* Primary relationship reported was not between infant anthros and milk component<br>(No) Association between [leptin] or total daily leptin intake and infant weight	None reported
Chan et al.<br>Canada, 2018 (HIC)	Longitudinal (cohort)<br><br>1094 analyzed	3-4 months postpartum	4 months, 1 year	Concentration in HM	Weight, length<br>(WHO Child Growth Standards)	(-) Association between breast milk leptin, insulin and infant WFL, BMI z-scores at 4 months<br>*these patterns of association persisted to 1 year	Prepregnancy maternal BMI, total breastfeeding duration, ethnicity, parity, diabetes, smoking, breastfeeding exclusivity, lactation stage

Table S3. Characteristics and results of included studies reporting on human milk hormones and infant anthropometrics - organized by component.

Authors, country, publication year (income setting)	Design and participants	Timing of milk sampling	Timing of infant anthropometrics	Estimated intake or HM concentration*	Anthropometric outcome measures and standards	Associations**	Major confounders considered
Cheema et al. Australia, 2021 (HIC)	Cohort (67 enrolled, 57 analyzed)	2 months	3 months	Estimated intake and concentrations in HM	Weight, length, BMI, head circumference, Fat Free Mass, Fat Free Mass Index, Fat Mass, Fat Mass Index, % Fat Mass, Fat Mass/Fat Free Mass (ratio) and z-scores<br><br>(WHO standards)	(no) association between leptin and infant anthropometrics - BIO	Infant birth weight, infant sex, gestational age, and 24-h milk intake
Doneray et al., Turkey , 2009. (UMIC)	Cohort. (15 mother-infant dyads enolled and analyzed)	1 day postpartum (average of 6.07 ± 1.94 h after birth) and 21-30 days postpartum	1 day postpartum, and 21-30 days postpartum	Concentration in HM	Weight and height, BMI, delta BMI	No reported association between infant anthropometrics and HM leptin. Noted (-) correlation between delta BMI and leptin concentration (r= -0.529, p<0.05)	None reported
Dundar et al. Turkey, 2005 (UMIC)	Longitudinal (47 enrolled)	Day 15 postpartum, 1, 2, 3 months	Birth weight, weight gain during first 15 days postpartum, weight gain during the first 1 month	Concentrations in HM	Day 15 postpartum, 1, 2, 3 months	(+) Association between birth weight and leptin levels at 15 days postpartum (r = 0.47, p = 0.01)<br>(-) Association between weight gain during first 15 days and leptin at 15 days postpartum (r = -0.44, p = 0.002)<br>(-) Association between weight gain during first month and leptin at 15 days postpartum (r = -0.40, p = 0.05)	None reported
Enstad et al. USA, 2019 (HIC)	Longitudinal (40 enrolled & analyzed)	1, 4 months	1, 2, 3, 4, 5, 6, 7 months	Concentrations in HM	Weight z-score, length z-score, BMI z-score, % fat mass, % lean mass, infant growth trajectory from month 1 to month 7	(+) Association between leptin and infant lean mass at 4 months<br>(-) Association between leptin and infant % fat mass at 4 months<br>(-) Association between leptin and infant BMI z-score at 1 month<br>(+) Association between leptin and % lean mass at 1 month and 4 months	Race, infant age at time of growth measurement, baseline infant measurement (measured at birth or month 1), sex
Fields et al. USA, 2017 (HIC)	Longitudinal (37 enrolled, 30 analyzed at 6 months)	1 month, 6 months	1 month, 6 months	Concentrations in HM	Weight, length, % fat, total fat mass, total fat free mass, trunk fat mass	(-) Association between month 1 leptin levels and month 6 body length, % fat, total fat mass, and trunk fat mass<br>(No) Association between month 1 leptin levels and total fat-free mass	Infant sex, pregravid maternal BMI category (normal weight, overweight, obese), stage of lactation (1 month vs. 6 months)
Galante et al. Finland, 2020 (HIC)	Cohort (501 mothers and 507 children enrolled)	2.6 +/- 0.4 months postpartum	13 months, 2, 3, 5 years	Concentration in HM	Weight, weight gain, BMI z-scores (Finnish population references)	(No) Associations for leptin reported (assumed no association)	Maternal pre-pregnancy BMI, infant sex, breastfeeding duration, intro of solid foods, infant birthweight
Gridneva et al., Australia. 2018 (HIC)<br><br>Gridneva et al., Australia. 2021 (HIC)	Cohort. 20 mother-infant dyads were enrolled	2, 5, 9, 12 months	2, 5, 9, 12 months	Estimated intake and concentration	Infant length, weight, BMI, head circumference, infant body composition, infant abdominal adiposity<br><br>(WHO standards)	(No) Association between leptin and infant anthropoetrics	None reported
Khaghani et al. Iran, 2006 (LMIC)	Longitudinal 244 enrolled & analyzed	1 month, 2 months, 3 months, 4 months, 5 months, 6 months	1 month, 2 months, 3 months, 4 months, 5 months, 6 months	Concentrations in HM	Weight, Height, Head circumference	(No) Association between leptin and infant height, weight, and head circumference	None reported
Khodabakhshi et al. Iran, 2015 (LMIC)	Cross-sectional (80 enrolled & analyzed)	Unclear	2, 4, 6 months	Concentration in HM	Weight, height	(No) reported Association between leptin and infant anthropometry (assumed no association)	None reported
Kon et al. Russia, 2014 (HIC)	Longitudinal (103 enrolled, 99 analyzed)	1, 2, 3 months postpartum	1, 2, 3 months postpartum	Concentration in HM	Weight gain	(+) Association between infant high weight gain status and leptin levels at 2 and 3 months	None reported
Larson-Meyer et al. USA,, 2020 (HIC)	Cohort (24 enrolled & analyzed)	1 and 6 months postpartum	1, 6, 12 months	Concentration in HM	WAZ, weight gain (WHO growth standards)	(-) Association between average milk leptin at 1 month and infant WAZ at 12 months (p = 0.03)	None reported
Larsson et al. Denmark, 2018 (HIC)	Longitudinal (59 enrolled, 30 analyzed)	First visit: 5-6.5 months old; Second visit: 9 months (+/- 2 weeks); Third visit for HW-group only: 18 months +/- 4 weeks	First visit: 5-6.5 months old;<br>Second visit: 9 months (+/- 2 weeks);<br>Third visit for HW-group only: 18 months +/- 4 weeks	Estimated intake and concentration in HM but associations determined using concentrations in milk	Weight, recumbent length, mid-upper-arm circumference, head circumference, lower leg circumference, recumbent waist and thorax circumference, triceps and subscapular skinfold thickness, WAZ, LAZ, BAZ, triceps skinfold for age z-score (TSFZ), subscapular skinfold-for-age z-score (SSFZ)	(No) Association between milk concentrations of adiponectin, leptin, lysozyme, sIgA, lactoferrin and infant's anthropometry or change in z-scores from birth to the 5-month visit	Maternal fasting time, infant sex
Leghi et al. Australia, 2021 (HIC)	Open label crossover design (18 enrolled and analyzed)	Week 1 (baseline), week 2 and week 3 of intervention (Infant age not standardized)	Week 1 (baseline), week 2 and week 3 of intervention (Infant age not standardized)	Estimated intake and concentration in HM	Weight, length, head circumference, BMI, WFLZ, WFAZ, LFAZ,<br><br>(WHO standards)	(NO) outcomes reported for Hormones (Assumed no relationship)	Controlled for maternal BMI

Table S3. Characteristics and results of included studies reporting on human milk <u>hormones</u> and infant anthropometrics - organized by component.

Authors, country, publication year (income setting)	Design and participants	Timing of milk sampling	Timing of infant anthropometrics	Estimated intake or HM concentration*	Anthropometric outcome measures and standards	Associations**	Major confounders considered
Logan et al., Germany. 2019(HIC)	Cohort (combined analyses of data from two distinct cohorts). UBCS (n = 1042); SPATZ (n = 934)	6 weeks postpartum	2 days	Concentrations in HM	Child BMI or change in BMI z-scores from birth up to age 2 years.	(-) Association between 6 week leptin and BMI at 4-5 weeks<br>(+) Association between 6 week leptin and greater increases in BMI after 4-5 weeks<br>(No) Association between 6 month leptin and infant growth	Maternal (age, birth country, parity, education, BMI, history of smoking), birth (gestational age at delivery, delivery mode), and other factors associated with breastfeeding or human milk composition (breastfeeding duration, duration of lactation up to sampling, exclusive or partial breastfeeding at the time of sampling, estimated feedings per day, breastfeeding method (breast or pump), collection time on the day, and time from last breastfeeding) were considered for inclusion in adjusted models for association with child BMI outcomes.
Miralles et al.<br>Spain, 2006<br>(HIC)	Longitudinal<br>28 enrolled & analyzed	1 month, 3 months, 6 months, 9 months postpartum	1 month, 12 months, 24 months	Concentrations in HM	BMI, Body weight, Body weight gain	(-) Association between leptin and infant BMI at 2 years<br>(No) Association between leptin and body weight or body weight gain at all ages	None reported
Mohamad et al.<br>Malaysia. 2018. UMIC	Cohort. 155 enrolled	0 and 2 months postpartum	Birth, 2, 6, 12 months	Concentration in HM	Infant body weight, infant BMI-for-age Z-score (BAZ)	(No) Association between breastmilk leptin and infant anthropometrics	Gestational weight gain,gestational age, maternal age,pre-pregnancy BMI,Infant sex, breastfeeding patterns, breastfeeding exclusivisity(exclusive, partial or no breastfeeding)
Nuss et al.<br>USA, 2019<br>(HIC)	Cross-sectional<br>(33 enrolled & analyzed)	One sample, between 4-8 weekspostpartum	One sample, between 4-8 weekspostpartum	Concentrations in HM	Weight, length, head circumference, % fat mass	(-) Association between leptin and infant weight<br>(-) Association between leptin and infant head circumference<br>(-) Association between leptin and % fat mass<br>(No) Association between leptin and Infant length	Infant age at visit
Pundir et al.<br>Australia, 2020<br>(HIC)	Cohort<br>(18 enrolled)	2, 5, 9, 12 months	2, 5, 9, 12 months	Concentrations in HM	Head circumference, % FM, l	(No) Association between breastmilk leptin and infant anthropometrics	None reported
Quinn et al., Nepal, 2017<br>(LMIC)	Cross-sectional. (50 women from Nubri and 66 women from Kathmandu)	NV=11.02 months +/-7.66; K=11.70 mos +/-8.44	NV=11.02 months +/-7.66;	Concentration in HM	Weight, length, head circumfe	(No) Association between Milk leptin with infant WAZ (Nubri)<br>(-) Association between milk leptin with infant WAZ (Katmandu)	Infant age, transferred milk volume, infant sex, birth order, nursing frequency and, in the Nubri Valley sub-set, altitude of residence.
Savino et al. Italy.2012<br>(HIC)	Cross-sectional. 41 enrolled (23 exclusively breastfed, 18 formula fed)	Between 0-6 months postpartum	Between 0-6 months postpartum	Concentrations in HM	Weight, length, BMI, head circumference, Fat Free Mass, Fat Free Mass Index, Fat Mass, Fat Mass Index, % Fat Mass, Fat Mass/Fat Free Mass (ratio) and z-scores<br><br>(WHO standards)	Report no correlations between breastmilk hormones and infants anthropometric parameters but no numerical data given	None reported
Schueler et al. 2013<br>USA (HIC)	Cohort. 13 mother-infant dyads enrolled and analyzed	29-38 days, 6 and 12 months	29-38 days, 6 and 12 months	Concentrations in HM	Infant weight	(No) Association between breastmilk leptin and infant anthropometrics	None reported
Schuster et al.,<br>Germany. 2011 (HIC)	Cohort. 23 mother-infant dyads enrolled	End of the first, second, third, and fourth week followed by the second, third, fourth, fifth, and sixth month PP	End of the first, second, third, and fourth week followed by the second, third, fourth, fifth, and sixth month PP	Concentrations in HM	Infant weight gain from birth to 6 months, infant weight gain from birth to 4 weeks PP	(-) Association between leptin at 1 week and infant weight gain upto 1-6 months.<br>Spearman correlation. Milk leptin after 1 week and weight gain from the end of the first to the sixth month(r=-0.681, p=0.007). milk leptin after 1 week and weight gain from 1 to 4 weeks (r=0.154, p=0.51)	None reported
Sims et al.<br>USA, 2020<br>(HIC)	Longitudinal<br>(284 enrolled, 174 analyzed)	Postnatal age 0.5, 1, 2, 3, 4, 5, 6, and 9 months	Postnatal age 0.5, 1, 2, 3, 4, 5, 6, and 9 months	Estimated intake and concentrations in HM, but associations only found/reported for estimated intake.	Weight, length, LFA z-score, WFA z-score, WFL z-score, fat mass, fat-free mass, FMI, FFMI	(No) Association between Leptin and WFA & WFL<br>+ Association between Leptin and LFA<br>(-) Association between daily intake of leptin with FMI	Infant sex, feeding mode (exclusive vs. mixed)
Ucar et al.<br>Turkey, 2000<br>(UMIC)	Cross-sectional<br>(18 enrolled & analyzed)	Once, between 3 and 120 days old	Once, between 3 and 120 days old	Concentration in HM	Weight, BMI, triceps skinfold thickness, left upper arm circumference measurements	(No) Association between log leptin concentration and infant's body weight, BMI, triceps skinfold thickness, and left upper arm circumference measurements	None reported
Uysal et al.<br>Turkey, 2002<br>(UMIC)	Cross-sectional<br>50 enrolled & analyzed	68 – 126 days postpartum	68 – 126 days postpartum	Concentration in HM	BMI	(No) Association between leptin and infant BMI	None reported
Weyermann et al.<br>Germany, 2007 (HIC)	Cohort.<br><br>1066 enrolled<br>767 analyzed	Range 33 and 71 days postpartum (mean 44 days; median 43 days; 10th-90th percentile 39–48 days)	6 weeks postpartum	Concentration in HM	Infant BMI, overweight risk (German reference population)	No clear relationship between risk of overweight and breast milk leptin levels	Age of mother (years), school education of mother (9 years; 10 years), nationality of mother (German; Turkish; other), BMI of mother at the beginning of pregnancy (kg/m2), smoking status of mother during follow-up (no; yes), and birthweight (grams). In addition to analyses in the overall sample, specific analyses were carried out according to duration of breast-feeding (6 months; 6 months).

Table S3. Characteristics and results of included studies reporting on human milk hormones and infant anthropometrics - organized by component.

Authors, country, publication year (income setting)	Design and participants	Timing of milk sampling	Timing of infant anthropometrics	Estimated intake or HM concentration*	Anthropometric outcome measures and standards	Associations**	Major confounders considered
Yis et al. Turkey, 2010 (UMIC)	Cohort. 24 infants were in the exclusively breastfed group	80-135 days postpartum	80-135 days postpartum	Concentration in HM	Weight, length, head circumfe	(No) Association between breastmilk ghrelin or breastmilk leptin and infant anthropometrics. Neither breast-milk ghrelin nor breast-milk leptin was correlated with the respective serum ghrelin or leptin, anthropometric data or bowel movement patterns of the infants in the BF group.	Breastfeeding exclusivity
Yu et al. Beijing, 2018 (UMIC)	Longitudinal<br><br>(121 enrolled, 96 analyzed for day 3 colostrum, 78 analyzed for day 42 mature milk, 61 analyzed for day 90 mature milk)	Colostrum on day 3 postpartum, mature milk on day 42 and 90	Days 3, 42, 90 postpartum	Concentration in HM	Weight, length, head circumference, weight-for-height gain	(No) Association between breastmilk leptin and infant anthropometrics	Maternal gestational diabetes status
Zamanillo et al., Spain, 2019 (HIC)	Longitudinal<br><br>59 infant-mother dyads. (38 normal-weight and 21 overweight/obese (BMI ≥ 25)	30, 60, 90 days postpartum	30, 60, 90 days postpartum	Concentration in HM	Infant BMI	(No) Associations between HM analytes of interest and infant anthros.	Maternal BMI
**Insulin (9 studies, 10 articles - 1,189 dyads)**							
Andreas et al. England, 2016 (HIC)	Cohort (105 enrolled)	1 week and 3 months postpartum	1 week and 3 months postpartum	Concentration in HM	Weight, length, head circumference, weight change from birth to 7 days, weight change from 7 days to 3 months, weight change from birth to 3 months	(No) Association between hormone concentrations in foremilk samples collected at 1 week postpartum and infant anthros<br><br>NOT PREDICTIVE:<br>(-) Association between infant weight and hindmilk insulin at 1 week postpartum (Pearson = -0.23, r = 0.03)<br>(-) Association between infant length at 1 week and foremilk insulin at 3 months postpartum (Pearson = -0.21, r = 0.04)<br>(No) Association between infant anthros and hormone concentrations in hindmilk samples collected at 3 months postpartum	None reported
Chan et al. Canada, 2018 (HIC)	Longitudinal (cohort)<br><br>1094 analyzed	3-4 months postpartum	4 months, 1 year	Concentration in HM	Weight, length (WHO Child Growth Standards)	(-) Association between breast milk leptin, insulin and infant WFL, BMI z-scores at 4 months<br>*these patterns of association persisted to 1 year	Prepregnancy maternal BMI, total breastfeeding duration, ethnicity, parity, diabetes, smoking, breastfeeding exclusivity, lactation stage
Cheema et al. Australia, 2021 (HIC)	Cohort (67 enrolled, 57 analyzed)	2 months	3 months	Estimated intake and concentrations in HM	Weight, length, BMI, head circumference, Fat Free Mass, Fat Free Mass Index, Fat Mass, Fat Mass Index, % Fat Mass, Fat Mass/Fat Free Mass (ratio) and z-scores<br><br>(WHO standards)	(no) association between insulin and infant anthropometrics - BIO	Infant birth weight, infant sex, gestational age, and 24-h milk intake
Ellsworth et al. USA, 2020 (HIC)	Longitudinal (55 enrolled, 32 analyzed)	2 weeks (average of 16 days postpartum)	2 weeks, 2 months, 6 months	Concentrations in HM	WFA z-score change, WFL z-score change, BMI z-score change, LFA z-score change, HCA z-score change (WHO multicentre Growth Reference Study Group, 2006)	(+) Association between milk insulin and infant WFA from 2 weeks to 6 months and HCA Z-score change from 2 weeks to 2 months in infants receiving any type of nutrition	Infant sex, *
Fields et al. USA, 2017 (HIC)<br><br>Goran et al. USA, 2017 (HIC)	Longitudinal (37 enrolled, 30 analyzed at 6 months)	1 month, 6 months	1 month, 6 months	Concentrations in HM	Weight, length, % fat, total fat mass, total fat free mass, trunk fat mass	(No) Association between month 1 insulin levels and month 6 body length, % fat, total fat mass, total fat-free mass and trunk fat mass	Infant sex, pregravid maternal BMI category (normal weight, overweight, obese), stage of lactation (1 month vs. 6 months)
Leghi et al. Australia, 2021 (HIC)	Open label crossover design (18 enrolled and analyzed)	Week 1 (baseline), week 2 and week 3 of intervention (Infant age not standardized)	Week 1 (baseline), week 2 and week 3 of intervention (Infant age not standardized)	Estimated intake and concentration in HM	Weight, length, head circumference, BMI, WFLZ, WFAZ, LFAZ,<br><br>(WHO standards)	(NO) outcomes reported for Hormones (Assumed no relationship)	Controlled for maternal BMI
Nuss et al. USA, 2019 (HIC)	Cross-sectional (33 enrolled & analyzed)	One sample, between 4-8 weekspostpartum	One sample, between 4-8 weekspostpartum	Concentrations in HM	Weight, length, head circumference, % fat mass	(-) Association between Insulin and infant weight<br>(-) Association between Insulin and infant head circumference<br>(-) Association between Insulin and % fat mass<br>(No) Association between Insulin and Infant length	Infant age at visit
Sims et al. USA, 2020 (HIC)	Longitudinal (284 enrolled, 174 analyzed)	Postnatal age 0.5, 1, 2, 3, 4, 5, 6, and 9 months	Postnatal age 0.5, 1, 2, 3, 4, 5, 6, and 9 months	Estimated intake and concentrations in HM, but associations only found/reported for estimated intake.	Weight, length, LFA z-score, WFA z-score, WFL z-score, fat mass, fat-free mass, FMI, FFMI	(No) Association between Insulin and LFA, WFA & WFL<br>(+) Association between daily intake of insulin with FMI<br>Analysis of effect of human milk composition on growth, stratified by normal-weight and overweight maternal BMI groups:<br>- effects of insulin on FMI driven by overweight group	Infant sex, feeding mode (exclusive vs. mixed)

Table S3. Characteristics and results of included studies reporting on human milk hormones and infant anthropometrics - organized by component.

Authors, country, publication year (income setting)	Design and participants	Timing of milk sampling	Timing of infant anthropometrics	Estimated intake or HM concentration*	Anthropometric outcome measures and standards	Associations**	Major confounders considered
Yu et al.<br>Beijing, 2018<br>(UMIC)	Longitudinal<br><br>(121 enrolled, 96 analyzed for day 3 colostrum, 78 analyzed for day 42 mature milk, 61 analyzed for day 90 mature milk)	Colostrum on day 3 postpartum, mature milk on day 42 and 90	Days 3, 42, 90 postpartum	Concentration in HM	Weight, length, head circumference, weight-for-height gain	(-) Association between adiponectin, insulin and head circumference during follow-up period (i.e. after day 3), but insulin association was insignificant after Bonferonni correction	Maternal gestational diabetes status
**Ghrelin (9 studies and articles - 701 dyads)**							
Andreas et al.<br>England, 2016<br>(HIC)	Cohort<br>(105 enrolled)	1 week and 3 months postpartum	1 week and 3 months postpartum	Concentration in HM	Weight, length, head circumference, weight change from birth to 7 days, weight change from 7 days to 3 months, weight change from birth to 3 months	(No) Association between hormone concentrations in foremilk samples collected at 1 week postpartum and infant anthros<br><br>NOT PREDICTIVE:<br>(No) Association between infant anthros and hormone concentrations in hindmilk samples collected at 3 months postpartum	None reported
Cesur et al.<br>Turkey, 2012<br>(UMIC)	Longitudinal<br>(25 enrolled, 19 analyzed)	Month 1 and 4 months postpartum	Month 1 and 4 months pos	Concentration in HM	Weight, weight gain, BMI	(+) Association between level of 4th month breast milk Active Ghrelin concentrations and weight gain of infant during study period	None reported
Guler et al. Turkey.<br>(UMIC)<br>2021	Cohort<br>(40 mother-infant dyads enrolled and analysed)	2 months (60 days, SD=10)	2 months (60 days, SD=10)	Concentration in HM	Weight, length, head circumference, weight-for-length z-score<br><br>(WHO standards)	(no) association between Leptin and infant weight for length Z score at 2 months<br><br>(no) association between ghrelin and infant weight for length Z score at 2 months<br><br>(no) association between adiponectin and infant weight for length Z score at 2 months<br><br>(no) association between IGF-1 and infant weight for length Z score at 2 months	Maternal BMI, age, parity, gestational weight gain and sex
Khodabakhshi et al.<br>Iran, 2015<br>(LMIC)	Cross-sectional<br>(80 enrolled & analyzed)	Unclear	2, 4, 6 months	Concentration in HM	Weight, height	(-) Association between ghrelin, EGF-1 and weight status (normal weight infants' mothers' milk had higher concentrations of both ghrelin and EGF1	None reported
Kon et al.<br>Russia, 2014<br>(HIC)	Longitudinal<br>(103 enrolled, 99 analyzed)	1, 2, 3 months postpartum	1, 2, 3 months postpartum	Concentration in HM	Weight gain	(+) Association between infant high weight gain status and ghrelin at 1 and 2 months	None reported
Larson-Meyer et al.<br>USA,, 2020<br>(HIC)	Cohort<br>(24 enrolled & analyzed)	1 and 6 months postpartum	1, 6, 12 months	Concentration in HM	WAZ, weight gain<br>(WHO growth standards)		None reported
Yis et al. Turkey, 2010<br>(UMIC)	Cohort. 24 infants were in the exclusively breastfed group	80-135 days postpartum	80-135 days postpartum	Concentration in HM	Weight, length, head circumfe	(No) association between breastmilk ghrelin or breastmilk leptin and infant anthropometrics. Neither breast-milk ghrelin nor breast-milk leptin was correlated with the respective serum ghrelin or leptin, anthropometric data or bowel movement patterns of the infants in the BF group.	Breastfeeding exclusivity
Yu et al.<br>Beijing, 2018<br>(UMIC)	Longitudinal<br><br>(121 enrolled, 96 analyzed for day 3 colostrum, 78 analyzed for day 42 mature milk, 61 analyzed for day 90 mature milk)	Colostrum on day 3 postpartum, mature milk on day 42 and 90	Days 3, 42, 90 postpartum	Concentration in HM	Weight, length, head circumference, weight-for-height gain		Maternal gestational diabetes status
**IGF-1 (5 studies/articles - 820 dyads)**							
Galante et al.<br>Finland, 2020<br>(HIC)	Cohort<br>(501 mothers and 507 children enrolled)	2.6 +/- 0.4 months postpartum	13 months, 2, 3, 5 years	Concentration in HM	Weight, weight gain, BMI z-scores<br>(Finnish population references)	(-) Association between IGF-1 and weight gain from birth to 2 years<br>(+) Association between IGF-1 and Weight Z at 13 months<br>(-) Association between IGF-1 and Weight Z at 3 and 5 years<br>(+) Association between IGF-1:cGP ration and Weight Z at 13 months<br>(-) Association between IGF-1:cGP ratio and BMI-Z at 3 and 5 years	Maternal pre-pregnancy BMI, infant sex, breastfeeding duration, intro of solid foods, infant birthweight
Guler et al. Turkey.<br>(UMIC)<br>2021	Cohort<br>(40 mother-infant dyads enrolled and analysed)	2 months (60 days, SD=10)	2 months (60 days, SD=10)	Concentration in HM	Weight, length, head circumference, weight-for-length z-score<br><br>(WHO standards)	(no) association between Leptin and infant weight for length Z score at 2 months<br><br>(no) association between ghrelin and infant weight for length Z score at 2 months<br><br>(no) association between adiponectin and infant weight for length Z score at 2 months<br><br>(no) association between IGF-1 and infant weight for length Z score at 2 months	Maternal BMI, age, parity, gestational weight gain and sex
Khodabakhshi et al.<br>Iran, 2015<br>(LMIC)	Cross-sectional<br>(80 enrolled & analyzed)	Unclear	2, 4, 6 months	Concentration in HM	Weight, height		None reported
Kon et al.<br>Russia, 2014<br>(HIC)	Longitudinal<br>(103 enrolled, 99 analyzed)	1, 2, 3 months postpartum	1, 2, 3 months postpartum	Concentration in HM	Weight gain	(+) Association between infant high weight gain status and levels of IGF-1 in breast milk at all lactation ages<br>(No) Association between infant weight gain status and levels of IGF-1 in breast milk at all lactation ages	None reported

Table S3. Characteristics and results of included studies reporting on human milk hormones and infant anthropometrics - organized by component.

Authors, country, publication year (income setting)	Design and participants	Timing of milk sampling	Timing of infant anthropometrics	Estimated intake or HM concentration*	Anthropometric outcome measures and standards	Associations**	Major confounders considered
Saso et al. The Gambia, 2018 (LIC)	Subset of larger cohort. N=100 mother-infant pairs	Birth (colostrum), day 60-89 postpartum	Birth, day 60-89 postpartum	Concentration in HM	Change in WAZ between birth and final visit, WAZ at final visit.	Cytokine levels in mature breast milk were weakly predictive of poor infant growth, possibly reflecting a “read-out” of suboptimal maternal health and nutrition. When adjusted for maternal anemia (as a proxy for maternal nutrition), TNFα and IL6 remained significant predictors ($p < 0.05$). IL6 R^2: 0.17. p<001. TNFa R^2 0.17, $p < 0.01$.	None reported
**Progesterone (1 study/article - 19 dyads)**							
Messripour et al. Iran, 2002 (LMIC)	Longitudinal (23 enrolled, 19 analyzed)	1, 6 months postpartum	1, 6 months postpartum	Concentration in HM	Height, weight, head circumference	(No) Association between milk hormone levels and growth indices after first month of life<br>(-) Association between FSH, LH, progesterone and weight after 6 months of life	None reported
**Luteinizing Hormone (LH; 1 study/article - 19 dyads)**							
Messripour et al. Iran, 2002 (LMIC)	Longitudinal (23 enrolled, 19 analyzed)	1, 6 months postpartum	1, 6 months postpartum	Concentration in HM	Height, weight, head circumference	(No) Association between milk hormone levels and growth indices after first month of life<br>(-) Association between FSH, LH, progesterone and weight after 6 months of life	None reported
**Follicle Stimulating Hormone (FSH; 1 study/article - 19 dyads)**							
Messripour et al. Iran, 2002 (LMIC)	Longitudinal (23 enrolled, 19 analyzed)	1, 6 months postpartum	1, 6 months postpartum	Concentration in HM	Height, weight, head circumference	(No) Association between milk hormone levels and growth indices after first month of life<br>(-) Association between FSH, LH, progesterone and weight after 6 months of life	None reported
**Cortisol (2 studies/articles - 60 dyads)**							
Hollanders et al. Netherlands, 2019 (HIC)	Longitudinal (42 enrolled, 42 analyzed for growth trajectories, 39 analyzed for infant body composition)	30 (+/- 5 days) postpartum	1, 2, 3 months	Concentration in HM	Length, weight, BMI, FMI, FFMI, % fat	(No) Association between glucocorticoid rhythmicity at 1 month and infant body composition or growth at 3 months	HADS-Score, maternal pre-pregnancy BMI, ethnicity (Caucasian vs. Non-caucasian, socioeconomic status, weight gain during pregnancy, parity, mode of delivery, mode of breastmilk at 3 months (i.e. < or > 80% breastmilk), infant sex, birth weight, gestational age
Pundir et al. Australia, 2020 (HIC)	Cohort (18 enrolled)	2, 5, 9, 12 months postpartum	2, 5, 9, 12 months	Concentration in HM	Head circumference, % FM, length, weight, BMI	(+) Association between cortisol and head circumference (rs = 0.25, p = 0.05)<br>(+) Association between cortisol and %FM (rs = 0.27, p = 0.03)<br>(No) Association between cortisol and infant length, weight, and BMI<br>(No) Association between cortisone and any infant parameters<br>(+) Association between cortisol/cortisone ratio and %FM (rs = 0.24, p = 0.01)<br>(+) Association between cortisol/cortisone ratio and BMI (rs = 0.28, p = 0.032)	None reported
**Resistin (2 studies/articles - 146 dyads)**							
Savino et al. Italy.2012 (HIC)	Cross-sectional. 41 enrolled (23 exclusively breastfed, 18 formula fed)	Between 0-6 months postpartum	Between 0-6 months postpartum	Concentrations in HM	Weight, length, BMI, head circumference, Fat Free Mass, Fat Free Mass Index, Fat Mass, Fat Mass Index, % Fat Mass, Fat Mass/Fat Free Mass (ratio) and z-scores<br><br>(WHO standards)	(No) Association between breastmilk Resisten and infant anthropometrics but no numerical data given	None reported
Andreas et al. England, 2016 (HIC)	Cohort (105 enrolled)	1 week and 3 months postpartum	1 week and 3 months postpartum	Concentration in HM	Weight, length, head circumference, weight change from birth to 7 days, weight change from 7 days to 3 months, weight change from birth to 3 months	(No) Association between hormone concentrations in foremilk samples collected at 1 week postpartum and infant anthros<br><br>NOT PREDICTIVE:<br>(No) Association between infant anthros and hormone concentrations in hindmilk samples collected at 3 months postpartum	None reported
**Irisin (1 study/article - 66 dyads)**							
Fatima et al. Pakistan, 2018 (LMIC)	Cohort (66 enrolled & analyzed)	72 hours postpartum, 6 weeks postpartum	Newborn weight, 6 weeks postpartum	Concentration in HM	Weight	(+) Association between irisin and baby weight at 6 weeks (r = 0.325, p = 0.017)<br>(+) Association between mature breast milk irisin and baby weight at 6 weeks (r = 0.296, p = 0.022)<br>**** association is lost when adjusted for maternal BMI	Maternal BMI, stratified by GDM status
**GLP-1 (2 studies/articles - 37 dyads)**							
Larson-Meyer et al. USA,, 2020 (HIC)	Cohort (24 enrolled & analyzed)	1 and 6 months postpartum	1, 6, 12 months	Concentration in HM	WAZ, weight gain (WHO growth standards)	(-) Association between average milk GLP-1 and WAZ at 6 months (r = -0.46)	None reported
Schueler et al. 2013 USA (HIC)	Cohort. 13 mother-infant dyads enrolled and analyzed	29-38 days, 6 and 12 months	29-38 days, 6 and 12 months	Concentrations in HM	Infant weight	(-) Association between hindmilk GLP-1 at 1 months after delivery and infant weight gain over the first 6 months (r 1=-0.67, P 1= 0.034; n =10).<br>(-) Association between GLP-1 concentration and infant weight-for-length percentile at 6 months.	None reported
**PYY (2 studies/articles - 37 dyads)**							
Schueler et al. 2013 USA (HIC)	Cohort. 13 mother-infant dyads enrolled and analyzed	29-38 days, 6 and 12 months	29-38 days, 6 and 12 months	Concentrations in HM	Infant weight	(-) Association between hindmilk GLP-1 at 1 months after delivery and infant weight gain over the first 6 months (r 1=-0.67, P 1= 0.034; n =10).<br>(-) Association between GLP-1 concentration and infant weight-for-length percentile at 6 months.	None reported

Table S3. Characteristics and results of included studies reporting on human milk hormones and infant anthropometrics - organized by component.

Authors, country, publication year (income setting)	Design and participants	Timing of milk sampling	Timing of infant anthropometrics	Estimated intake or HM concentration*	Anthropometric outcome measures and standards	Associations**	Major confounders considered
Larson-Meyer et al.<br>USA,, 2020<br>(HIC)	Cohort<br>(24 enrolled & analyzed)	1 and 6 months postpartum	1, 6, 12 months	Concentration in HM	WAZ, weight gain<br>(WHO growth standards)		None reported

*Values reported as mean ± SD or median (IQR). **"No (assumed) associations = unreported associations assumed to be no association.

Abbreviations: BF, breastfeeding; HIC, high income countries; mo, months; HM, human milk; LMIC, low and middle income countries; NCHS, National Center for Health Statistics; RCT, randomized controlled trial; SCM, subclinical mastitis; WHO, World Health Organization; wks, weeks

Anthropometrics: BMI, body mass indez; HAZ, height for age z-score; HC, head circumference; HCAZ, head circumference z-score; LAZ, length for age Z-score; LFA, length for age; WAZ, weight for age z-score; WFA, weight for age; WLZ, weight-for-length z-score

Table S4. Characteristics and results of included studies reporting on human milk oligosaccharides (HMOs) and infant anthropometrics - organized by component.

Authors, country, publication year (income setting)	Design and participants	Timing of milk sampling	Timing of infant anthropometrics	Estimated intake or HM concentration*	Anthropometric outcome measures and standards	Associations**	Major confounders considered
**HMOs (13 studies/articles - 2,640 dyads)**							
Alderete et al.<br>USA, 2015<br>(HIC)	Longitudinal<br>(37 enrolled, 25 analyzed)	1, 6 months postpartum	1, 6 months	Concentration in HM:<br>2'-FL<br>3-FL<br>3'-SL<br>DFLNH<br>DFLNT<br>DSLNH<br>DSLNT<br>FDSLNH<br>FLNH<br>LNFPI<br>LNFPII<br>LNFPIII<br>LNnT<br>LNT<br>LSTb<br>LSTc<br>Sum<br>Diversity<br>Evenness	Weight, length, % fat, total fat, lean mass, trunk fat mass	1 month:<br>(-) Association between LNFPI and infant weight<br>(-) Association between HMO diversity and 1 month fat mass and % body fat<br>(-) Association between HMO evenness and 1 month fat mass and % body fat<br><br>6 months:<br>(+) Association between DSLNT and length<br>(-) Association between LNFPI and weight, lean mass, and fat mass<br>(+) Association between DSLNT and fat mass<br>(+) Association between LNFPII and fat mass<br>(-) Association between LNnT and body fat<br>(+) Association between FDSLNH and body fat<br>(-) Association between 1 month LNFPII and 6 month fat mass<br><br>Specific HMOs accounting for significant increase in percentage of observed variance in infant body composition: 1 month<br>- including LNFPI explained 18% more of variance in infant weight; HMO diversity and HMO evenness explained 20% more of variance in % fat and 17% more of variance in fat mass<br><br>Specific HMOs accounting for significant increase in percentage of observe red variance in infant body composition: 6 months<br>- including DSLNT explained 7% more of variation in length; including LNFPI explained 6% more of variance in weight and 13% more of variation in lean mass; including LNFPI, DSLNT, AND FDSLNH explained 33% more of variation in infant fat mass; including LNnT explained 23% more of variance in percentage fat	Maternal pre-pregnancy BMI, pregnancy weight gain, infant sex, infant age in days at 1 month
Binia et al. multi centre (Europe), 2021<br>(HIC)	Cohort<br>(375 enrolled, 350 analysed)	2 (0–3) days,<br>17 ± 3 days,<br>30 ± 3 days,<br>60 ± 5 days,<br>90 ± 5 days, and<br>120 ± 5 days	2 (0–3) days,<br>17 ± 3 days,<br>30 ± 3 days,<br>60 ± 5 days,<br>90 ± 5 days, and<br>120 ± 5 days)	Concentrations in HM:<br>2'-FL<br>3-FL<br>6'-GL<br>3'-SL<br>6'-SL<br>LDFT (DiFL)<br>LNT<br>LNnT<br>LNFP-I<br>LNFP-II<br>LNFP-III<br>LNFP-V<br>LNnFP-V<br>LSTb<br>LSTc<br>LNDFH-I<br>LNnDFH<br>DSLNT MFLNH-III DFLNHa	Weight, length, head circumference, weight-for-length, Fat mass index, weight change rate, length change rate, head circumference change rate, weight-for-length change rate<br><br>(WHO Standards)	(-) Association between 3'-SL and length<br><br>(+) Association between MFLNH-III, LNFP-III and head circumference<br><br>(-) Association between A-Tetra and change in head circumference<br><br>(-) Association between LNnT and change in length<br><br>(+) Association between LSTc and weight for length<br><br>(no) Association between any HMOs and FMI (or fat accretion)	Maternal postpartum BMI, infant sex, infant birth weight, and fat mass at visit one (V1)

Table S4. Characteristics and results of included studies reporting on human milk oligosaccharides (HMOs) and infant anthropometrics - organized by component.

Authors, country, publication year (income setting)	Design and participants	Timing of milk sampling	Timing of infant anthropometrics	Estimated intake or HM concentration*	Anthropometric outcome measures and standards	Associations**	Major confounders considered
Cheema et al. Australia, 2022<br>(HIC)	Cohort<br>(67 enrolled, 60 analyzed)	2 months	3 months	Estimated intake and concentration in HM:<br>2'FL,<br>3FL,<br>3'SL,<br>6'SL,<br>DFLac,<br>DFLNH,<br>DFLNT,<br>DSLNH,<br>DSLNT, FDSLNH,<br>FLNH,<br>LNFP I,<br>LNFP II,<br>LNFP III,<br>LNH,<br>LNnT,<br>LNT,<br>LSTb, and<br>LSTc	Weight, length, BMI, head circumference, Fat Free Mass, Fat Free Mass Index, Fat Mass, Fat Mass Index, % Fat Mass, Fat Mass/Fat Free Mass (ratio) and z-scores<br><br>(WHO standards)	**HM Concentrations**<br>**Non-Secretors:**<br>(+) association between [DFLNT] and weight, height, WFAZ, LFAZ and Fat mass<br>(-) association between [FLNH] and length, LFAZ<br><br>**Secretors:**<br>(+) association between [3'SL] and fat free mass<br><br>**Overall (not stratified between NS and Secretors):**<br>(-) association between [FLNH] and weight<br>(+) association between log[DFLNH] and weight, length, LFAZ, fat free mass,<br>(-) association between log[LNnT] and length, LFAZ<br>(-) association between log[LNFP III] and Fat mass (%) and Fat mass to fat-free mass ratio<br><br>**HM Daily Intakes**<br>**Non-Secretors:**<br>(+) association between 6'SL and weight, WFAZ, Fat mass and FMI<br>(-) association between log FDSLNH and BMI, WFAZ, BMI for Age Z, log FFMI<br><br>**Secretors:**<br>(+) association between log 3'SL and weight, length, WFAZ, log FFM, log FFMI<br><br>**Overall (not stratified between NS and Secretors):**<br>(+) association between 2'FL and Weight and Fat mass,<br>(+) association beteween 3FL and weight, length, WFAZ, LFAz, log FFM, log FFMI<br>(+) association between log(DFLaz) and weight, BMI, BMIZ, log FFM, log FFMI<br>(+) assocation between log(DFLNH) and weight, length, WFAZ, LFAZ, log FFM<br>(+) association between log(LSTb) and BMI<br>(+) association between DFLNT and BMI, BMIZ, and log FFMI.<br>(+) association between log(DFLNH) and weight, length, LFAZ, fat free mass,<br>(-) association between log[LNnT] and length, LFAZ<br>(-) association between log[LNFP III] and Fat mass (%) and Fat mass to fat-free mass ratio"	Infant birth weight, infant sex, gestational age, and 24-h milk intake, infant body composition, maternal body composition, maternal weight, Fat Free Mass, Fat Mass and Fat Mass Index

Table S4. Characteristics and results of included studies reporting on human milk oligosaccharides (HMOs) and infant anthropometrics - organized by component.

Authors, country, publication year (income setting)	Design and participants	Timing of milk sampling	Timing of infant anthropometrics	Estimated intake or HM concentration*	Anthropometric outcome measures and standards	Associations**	Major confounders considered
Davis et al.<br>The Gambia, 2017<br>(LIC)	Longitudinal RCT<br>(33 enrolled & analyzed)	4, 16, 20 weeks postpartum	Height and weight measured weekly (4, 16, 20 weeks postpartum)	Concentrations in HM:<br>LNTa<br>LNFP I+III<br>LNnT<br>LSTc<br>MFLNH I+III<br>MFpLNH IV LSTb<br>LNnH<br>DFLNHa<br>LNHa,<br>DFpLNH II<br>DFLNH<br>3'SL<br>4021a+S-LNnH II<br>IFLNH III<br>F-LNO<br>2'FL<br>LNDFH II<br>LDFT<br>4120a<br>TFLNH<br>p-LNH<br>F-LSTc<br>LNDFH I<br>5130a<br>5230a+DFLNnO I/DFLNO II<br>5330a<br>IFLNH I<br>DFLNnO II<br>DFLNO I<br>5130b<br>S-LNH<br>LSTa<br>LNFP II<br>6'SL<br>DFLNHc<br>5130c<br>4320a<br>3FL	WFA z-scores, HFA z-scores<br>(Gambian reference)	(+) Association between 3'SL and WAZ at 20 weeks<br>(-) Association between LSTc and WAZ at 20 weeks<br>(+) Association between DFLNHa and HAZ at 20 weeks<br>(+) Association between LNFP I + III and HAZ at 20 weeks	None reported

Table S4. Characteristics and results of included studies reporting on human milk oligosaccharides (HMOs) and infant anthropometrics - organized by component.

Authors, country, publication year (income setting)	Design and participants	Timing of milk sampling	Timing of infant anthropometrics	Estimated intake or HM concentration*	Anthropometric outcome measures and standards	Associations**	Major confounders considered
Jorgensen et al.<br>Malawi, 2020<br>(LIC)	Longitudinal<br>(1391 enrolled, 659 samples collected, 647 analyzed for HMOs and 637 analyzed for protein)	6 months postpartum	6, 12 months postpartum	Concentrations in HM: untargetted HMOs	Change in LAZ from 6-12 months, changing WAZ from 6-12 months, change in WLZ from 6-12 months, change in HCZ from 6-12 months	**For secretors + nonsecretors combined:**<br>In primary analyses:<br>(No) Association between abundance of groups of HMOs or concentrations of IgA, lactalbumin, or lactoferrin and infant growth indicators.<br><br>(+) Association between unnamed HMO 5311a and change in LAZ and WAZ.<br>(-) Association between unnamed HMO 5330a and change in HCZ.<br>(+) Association between unnamed HMO 5230b and change in WAZ and WLZ.<br>(+) Association between unnamed HMO 4320a and change in LAZ.<br>(+) Association between unnamed HMO 6400a and change in WAZ.<br>(+) Between unnamed HMO 6400b and WAZ.<br><br>**For secretors only:**<br>In primary analyses:<br>(+) Association between absolute abundance of all HMOs and change in LAZ.<br>In exploratory analyses:<br>(-) Association between LNT + LNnT and change in WLZ.<br>(-) Association between LNT and change in WAZ, WLZ, HCZ.<br>(-) Association between LNFP I + III and WLZ.<br>(-) Association between LDFT and change in LAZ.<br>(+) Association between LDFT and changein WLZ.<br>(+) Association between 3'SL and change in HCZ.<br>(+) Association between 5230a + DFLNnO I/DFLNO II and change in WAZ.<br>(-) Association between IFLNH I and change in WLZ.<br>(+) Association between LSTa and change in LAZ.<br>(-) Association between LSTa and change in WLZ<br>(+) Positive association between DFLNHc and change in LAZ.<br>(-) Negative association between DFLNHc and change in WLZ.<br>(-) Negative association between 6'SL and change in HCZ.<br>(+) Positive association between unnamed HMO 5130b and change in LAZ.<br>(+) Association between unnamed HMO 4240a and change in LAZ.<br><br>**For nonsecretors only:**<br>In primary analyses:<br>(No) Significant associations between bioactive proteins or groups of HMOs and infant growth.<br><br>*In exploratory analyses:*<br>(-) Association between LNFP II and change in WLZ.<br>(-) Association between unnamed HMO 4120a and change in WLZ.	Secretor status, baseline age, BMI, parity, education, food security, HIV status, Hb, household assets, residential location, season at time of sample collection, intervention group (consumed iron and folic acid during pregnancy, or consumed multiple micronutrient capsule during pregnancy and up to 6 months postpartum, or consumed a lipid-based nutrient supplement during pregnancy and up to 6 months postpartum), infant sex
Lagstrom et al.<br>Finland, 2020<br>(HIC)	Longitudinal<br>(1797 mothers and 1927 children enrolled, 802 mothers analyzed)	3 months postpartum	3, 6, 8 months, 1, 2, 3, 4, 5 years of ag	Concentration in HM:<br>2FL<br>3FL<br>LNnT<br>3SL<br>DFLac<br>6SL<br>LNT<br>LNFP I<br>LNFP II<br>LNFP III<br>LSTb<br>LSTc<br>DFLNT<br>LNH<br>DSLNT<br>FLNH<br>DFLNH<br>FDSLNH<br>DSLNH<br><br>Diversity<br>Sum of HMOs<br>HMO-bound Sialic acid<br>HMO-bound Fucose	Height z-score, weight z-score	**In secretors:**<br>(-) Association between HMO diversity and height and weight z-scores during first 12 months of life<br>(-) Association between HMO diversity and height z-scores throughout 1 and 5 years<br>(+) Association between 2'FL and height z-scores between 3 and 12 months, and 1 and 5 years<br>(+) Association between 2'FL and weight z-scores between 3 and 12 months<br>(-) Association between LNnT and weight and height z-scores throughout first five years of life<br>(+) Association between HMO-bound fucose and height z-scores and weight z-scores between 3 and 12 months, and 1-5 years<br>(-) Association between LSTb and height z-scores between 3 and 12 months<br>(+) Association between 3FL and weight z-scores from 3 to 13 months and 1-5 years<br>(+) Association between 3'SL and weight z-scores from 3-12 months and 1-5 years<br>(+) Association between DFLac and weight z-scores from 3-12 months<br>(-) Association between LSTb and weight z-scores from 3-12 months<br><br>**For non-secretors:**<br>(No) Association between HMO diversity and height z-scores	Maternal secretor status, maternal pre-pregnancy BMI, infant sex, birthweight z-score, time point (i.e. 3-12 months or 1-5 years)

Table S4. Characteristics and results of included studies reporting on human milk oligosaccharides (HMOs) and infant anthropometrics - organized by component.

Authors, country, publication year (income setting)	Design and participants	Timing of milk sampling	Timing of infant anthropometrics	Estimated intake or HM concentration*	Anthropometric outcome measures and standards	Associations**	Major confounders considered
Larsson et al. Denmark, 2019 (HIC)	Longitudinal<br>(30 enrolled, 30 analyzed at 5 months, 29 analyzed at 9 months)	Infant age 5 - 6.5 months, and 9 months	Birth, 5 months, 9 months	Concentration in HM:<br>2'FL,<br>3 FL<br>3'SL<br>6'SL,<br>DFLac,<br>LNH,<br>LNnT,<br>LNT,<br>LNFP I,<br>LNFP II,<br>LNFP III,<br>DFLNH<br>DFLNT<br>FLNH<br>LSTb<br>LSTc<br>DFLNT<br>DSLNH<br>FDSLNH<br>HMO bound Fucose<br>HMO Bound Sialic Acid<br>HMO sum<br>Diversity	WAZ, BAZ, HAZ, FMI, FFMI, weight velocity	**Analysis of Secretors only:**<br>(+) Association between 2'-FL and 0-5 months weight velocity<br>(+) Association between 2'-FL and FMI at 5 months<br>(+) Association between DFlac and weight velocity and length at 5 months<br>(+) Association between 3'-SL and length at 5 months<br>(-) Association between 6'SL and BAZ at 5 months<br>(-) Association between LNnT and length, weight velocity, FMI, and change in WAZ from birth to five months<br>(+) Association between total HMO-bound fucose and weight velocity 0-5 months and FMI<br>(+) Association between total HMO and weight velocity from 0-5 months and FMI at 5 months<br>(-) Association between HMO diversity and weight velocity and FMI at 5 months<br><br>**Analysis of secretors + non-secretors combined:**<br>(-) Association between LNnT and length<br>(-) Association between HMO diversity and BAZ, weight velocity, and FMI at 5 months	Maternal secretor status, infant sex
Liu et al. China, 2021 (UMIC)	Longitudinal<br>(110 mother-infant dyads)	4 weeks, 8 weeks and 12 weeks	4 weeks, 8 weeks and 12 weeks	Concentration in HM:<br>2'FL,<br>3'FL<br>3'S),<br>6'SL,<br>LNT<br>LNnT<br>LDFT/DFL, LNFP-I, LNFPIII<br>LNFP-II<br>DSLNT, I LNnDFH-II, LNDFH-II, LNDFH-I,<br>LSTb, LSTc 3'SLNFP-II MFLNnH, MFLNH-III MFLNH-I DFpLNnH, DFLNHa	Height, weight, head circumference	(+) association between LDFT, 3'SL and infant body weight at T2 (8 weeks).<br><br>(-) association between DSLNT (4 weeks) and LNT (4, 8 and 12 weeks) and infant body weight<br><br>(+) association between total fucosylated HMOs (4 weeks), total sialylated HMOs (8 weeks), 3'SL (8, 12 weeks) and BMI<br><br>(-) association between LNT (12 weeks) and BMI<br><br>(-) association between 3'SL (4 weeks), LNT (8 weeks), LNFP-1 (12 weeks) and DSLNT (4, 8, 12 weeks) and head circumference.	No infant birth data included in analysis
Menzel et al. Germany, 2021 (HIC)	Cohort<br>(153 enrolled, 145 analysed)	3 months	3 months, 6 months, 1 year, 2 years	Concentration in HM:<br>2'FL<br>3-FL<br>3'SL<br>6'SL<br>LNT<br>LNnT<br>LNFP-I<br>LNFP-V<br>LNnFP	Length, weight, head circumference, BMI (reported as SDs), growth velocity was calculated as the standardized difference between the 3-month height and the 1-year height of the child<br><br>Reported as SDs as per guidelines from the German Working Group on Obesity in Childhood and Adolescence	**Non-Secretors:**<br>(-) association between height and LNT at 2 years<br>(no) association between height and 2'FL, 3-FL, 3'SL, 6'SL, LNnFP, or LNFP-V.<br>(-) association between growth velocity and LNnT at 3m - 1 year and 1-2 years<br>(-) association between BMI-SDS and LNFP-V at 3m, 6m, 1 y and 2 y.<br>(-) association between BMI-SDS and 6' SL at 3m, and 1 y<br>(-) association between LNFP-V and head circumference at 3m, 1 y and 2 y.<br>(+) association between LNnFP and head circumference at 2 years<br><br>**Secretors:**<br>(+) association between height and LNFP-I at 3, 6 and 12 months<br>(no) association between height and 2'FL, 3-FL, 3'SL, 6'SL, LNnFP, or LNFP-V.<br>(-) association between growth velocity and LNFP-I at 1-2 years<br>(no) association between growth velocity and 2'FL,<br>(-) association between BMI-SDS and LNT and LNFP-V at 2 years<br>(+) associations between BMI-SDS and LNnFP at 2 years.<br>(-) association between BMI-SDS and 2'FL at 3m.<br>(no) association between BMI-SDS and LNFP-I<br>(+) association between LNFP-I and head circumference at 6m<br>(no) association between head circumference and 2'FL,<br><br>**Overall (not stratified between NS and Secretors):**<br>(no) association between height and 2'FL, 3-FL, 3'SL, 6'SL, LNnFP, or LNFP-V.<br>(-) association between growth velocity and LNT and LNFP-V at 3 m - 1 year<br>(no) association between growth velocity and 3-FL, 6'SL and LNnFP<br>(no) association between BMI-SDS and 3-FL and LNnT<br>(no) association between head circumference and 3-FL, 3'SL, 6'SL or LNT"	Secretor status, Maternal pre-pregnancy weight and height and infant birth parameters

Table S4. Characteristics and results of included studies reporting on human milk oligosaccharides (HMOs) and infant anthropometrics - organized by component.

Authors, country, publication year (income setting)	Design and participants	Timing of milk sampling	Timing of infant anthropometrics	Estimated intake or HM concentration*	Anthropometric outcome measures and standards	Associations**	Major confounders considered
Saben et al. USA. 2021 (HIC)	Cohorts<br>(194 maternal-infant dyads)	2 months	2 months, 6 months	Estimated intake and concentration in HM:<br>2'FL,<br>3 FL<br>3'SL<br>6'SL,<br>DFLac,<br>DFLNH<br>DFLNT<br>DSLNH<br>DSLNT<br>FDSLNH<br>FLNH<br>LNFP I,<br>LNFP II,<br>LNFP III,<br>LNH,<br>LNnT,<br>LNT,<br>LSTb<br>LSTc	Infant weight and length, WLZ, WAZ, FM, FFM	**All infants:**<br>(+) association between 3 FL and infant fat mass, WLZ, and WAZ at 2-6 months<br>(+) association between LNFP II and infant fat mass, WLZ and WAZ at 2-6 months<br>(+) association between LNFP III and infant fat mass at 2-6 months<br>(+) association between 3' SL and infant fat mass and WAZ at 2-6 months<br>(+) association between 6' SL and infant fat mass at 2-6 months<br>(+) association between LSTb and infant fat mass, WLZ and WAZ at 2-6 months<br>(+) association between DSLNT and infant fat mass at 2-6 months<br>(+) association between DSLNH and infant fat mass, WLZ and WAZ at 2-6 months<br>(+) association between Acidic HMOs and infant fat mass, WLZ, and WAZ at 2-6 months<br>(+) association between Total HMOs and infant fat mass, WLZ, and WAZ at 2-6 months<br><br>**EBF Infants only:**<br>(+) association between 3 FL and infant fat mass and WAZ at 2-6 months<br>(+) association between LNFP II and infant fat mass, WLZ and WAZ at 2-6 months<br>(+) association between 3' SL and infant fat mass and WAZ at 2-6 months<br>(+) association between 6' SL and infant fat mass at 2-6 months<br>(+) association between LSTb and infant fat mass WAZ at 2-6 months<br>(+) association between DSLNH and infant fat mass, WLZ and WAZ at 2-6 months<br>(+) association between Acidic HMOs and infant fat mass and WAZ at 2-6 months<br>(+) association between Total HMOs and infant fat mass and WAZ at 2-6 months	Infant birth weight, infant sex, infant age at time of measurement, maternal BMI, mode of delivery, and maternal secretor status<br>Breastfeeding status
Sprenger et al. Singapore, 2017 (HIC)	Longitudinal<br>(50 enrolled & analyzed)	30, 60, 120 days postpartum	Birth, 1, 2, 4 months	Concentrations in HM:<br>2'FL,<br>3'SL,<br>6'SL,<br>LNnT,<br>LNT	Weight, length, BMI, head circumference<br>(WHO child growth standards)	(No) Association between breast milk type (low 2'FL vs. high 2'FL) and body weight, length, BMI, and head circumference over the four months	Lactation stage, 2'FL (secretor) status, infant sex
Tonon et al. Brazil, 2019 (UMIC)	Cross-sectional<br>(78 enrolled & analyzed)	Once between 17 and 76 days of infant life	Once between 17 and 76 days of infant life	Concentration in HM:<br>2'-FL<br>3'-FL<br>LNFP I<br>LNDFH I<br>LNDFH II<br>DFLNHc<br>DFpLNnH<br>LNH<br>LNnH<br>LNT + LNnT<br>3'-SL<br>6'-SL<br>LSTa<br>LSTb<br>LSTc<br>Fucosylated<br>Neutral core<br>Acidic<br>Total HMOs	Weight, length, weight gain	**Based on Se and Le status:**<br>***For Se+Le+:***<br>(-) Association between LNDFH I, LNT + LNnT, 3'-SL, 6'-SL, LSTa, LSTb, LSTc acidic HMOs, total acidic, total neutral core, total fucosylated, total HMOs and infant weight<br>(-) Association between LNT + LNnT, 3'-SL, LSTa, LSTc, total neutral core, total acidic HMOs and infant length<br>(-) Association between LNDFH I, 6'-SL, LSTc, total fucosylated, total acidic, total HMOs and weight gain<br>***For Se+Le-:***<br>(-) Association between 3'-SL and weight<br>(-) Association between 6'-SL and length<br>(-) Association between LSTb and weight gain<br>***For Se-Le+:***<br>(No) Associations<br><br>**Based on Se status alone:**<br>***For Se+:***<br>(-) Association between LNFP I, LNT + LNnT, 3'-SL, 6'-SL, LSTa, LSTb, LSTc (acidic HMOs), total fucosylated, total neutral core, total acidic, total HMOs and weight<br>(-) Association between LNT + LNnT, 3'-SL, 6'-SL, LSTc, total acid HMOs and length<br>(-) Association between LNFP I, LNFDH I, 6'-SL, LSTc, total fucosylated, total acidic, total HMOs and weight gain<br>***For Se-:***<br>(-) Association between 6'-SL, LSTc and weight<br>(-) Association between 6'-SL, LSTc and length	None reported

Table S4. Characteristics and results of included studies reporting on human milk oligosaccharides (HMOs) and infant anthropometrics - organized by component.

Authors, country, publication year (income setting)	Design and participants	Timing of milk sampling	Timing of infant anthropometrics	Estimated intake or HM concentration*	Anthropometric outcome measures and standards	Associations**	Major confounders considered
Wang et al.<br>China, 2020<br>(UMIC)	Longitudinal<br>(269 enrolled, 116 analyzed)	4 points: 1-5 days, 8-14 days, 4 weeks (27-33 days), and 6 months (177-183 days) postpartum	4 points: 1-5 days, 8-14 days, 4 weeks (27-33 days), and 6 months (177-183 days) postpartum	Concentrations in HM:<br>3'-FL<br>LNT&LNnT<br>LNFP-III<br>LNFP-II<br>LNFP-IV<br>LNDFH-II<br>LNH<br>LNnH<br>MFLNH-I & III<br>MFpLNH-IV<br>IFLNH-III<br>DFLNH-b<br>DFpLNH-I<br>DFpLNH-II<br>DFLNO-I<br>Secretors<br>2'-FL<br>LDFT<br>LNT&LNnT −0.29<br>LNFP-III<br>LNFP-II<br>LNFP-I<br>LNFP-IV<br>LNDFH-I<br>LNDFH-II<br>LNH<br>LNnH<br>MFLNH-I & III<br>MFpLNH-IV<br>IFLNH-III<br>IFLNH-I<br>TFLNH-I<br>TFLNH-II<br>DFLNH-a&c<br>DFLNH-b<br>DFpLNH-I<br>DFpLNH-II<br>DFLNO-I	Weight gain, length gain	**For secretors:**<br>(+) association between infant length gain at month 1 LNH, LNnH, MFpLNH-IV, IFLNH-I AND DFLNH-a+c, colostrum, TFLNH-I AND DFLNH-b in transitional milk, and LNnH, MFpLNH-IV, IFLNH-III, TFLNH-I, TFLNH-II and DFLNH-b in mature milk<br><br>**For non-secretors:**<br>(-) 3'FL,LNDFH-II and weight gain @ 6 months<br>(+) LNT&LNnT and length gain 1 month<br>(+) LNnH and length gain at 2 and 6 months	Maternal secretor status

*Values reported as mean ± SD or median (IQR). **"No (assumed) associations = unreported associations assumed to be no association.

Abbreviations: BF, breastfeeding; HIC, high income countries; mo, months; HM, human milk; LMIC, low and middle income countries; NCHS, National Center for Health Statistics; RCT, randomized controlled trial; SCM, subclinical mastitis; WHO, World Health Organization; wks, weeks

Anthropometrics: BMI, body mass indez; HAZ, height for age z-score; HC, head circumference; HCAZ, head circumference z-score; LAZ, length for age Z-score; LFA, length for age; WAZ, weight for age z-score; WFA, weight for age; WLZ, weight-for-length z-score

HMOs: 2'FL, 2'Fucosyllactose; 3FL, 3 Fucosyllactose; 6'-SL, 6'-sialyllactose; DFLac, Difucosyllactose; DFLNT, Difucosyllacto-N-tetrose; DFLNH, Difucosyllacto-N-hexaose; DSLNT, Disialyllacto-N-tetraose, IFLNH-I, fucosyl-para-lacto-N-hexaose I; LNFPI, Lacto-N-fucopentaose I; LNFP II, Lacto-N-fucopentaose II; LNFP III, Lacto-N-fucopentaose III; LNH, Lacto-N-hexaose; LNT, Lacto-N-tetrose; LNnT, Lacto-N-neotetraose; LSTa, Sialyl-lacto-N-tetraose a; LSTb, Sialyl-lacto-N-tetraose b; LSTc, Sialyl-lacto-N-tetraose c; MFpLNH-IV, Monofucosyl-para-lacto-N-hexaose IV

Table S5. Characteristics and results of included studies reporting on human milk immunomodulatory components and infant anthropometrics - organized by component.

Authors, country, publication year (income setting)	Design and participants	Timing of milk sampling	Timing of infant anthropometrics	Estimated intake or HM concentration*	Anthropometric outcome measures and standards	Associations**	Major confounders considered
**IL-1 beta, 4 studies/articles**							
Enstad et al. USA, 2019 (HIC)	Longitudinal (40 enrolled & analyzed)	1, 4 months	1, 2, 3, 4, 5, 6, 7 months	Concentrations in HM	Weight z-score, length z-score, BMI z-score, % fat mass, % lean mass, infant growth trajectory from month 1 to month 7	(+) Association between IL-1beta and length z-scores in infants at 4 months<br>(+) Association between IL-1beta and infant BMI z-score at 7 months<br>(+) Association between cytokines and weight z-scores at birth<br>(-) Association between cytokines and weight z-scores between 1-2 months<br>(+) Association between cytokines and weight z-scores after 2 months	Race, infant age at time of growth measurement, baseline infant measurement (measured at birth or month 1), sex
Ortiz-Andrellucchi et al. Spain, 2008 (HIC)	RCT.<br>104 mothers (45assignemd to placebo/59 assigned to intreatment)<br>39 mothers analysed from placebo group/54 mothers analysed from treatment group<br>38 children analyzed from placebo group /51 children analyzed from treatment group	72 days, 10 days, and 45 days postpartum	Birth, 2 months, 6 months	Concentrations in HM	Infant weight	No significant differences between groups in relation to weight (data not shown).	None reported
Saso et al. The Gambia, 2018 (LIC)	Subset of larger cohort.<br><br>n=100 mother-infant pairs	Birth (colostrum), day 60-89 postpartum	Birth, day 60-89 postpartum	Concentrations in HM	Change in WAZ between birth and final visit, WAZ at final visit.	(No) Associations between IL-1 beta and infant growth<br><br>Cytokine levels in mature breast milk were weakly predictive of poor infant growth, possibly reflecting a “read-out” of suboptimal maternal health and nutrition. When adjusted for maternal anemia (as a proxy for maternal nutrition), TNFα and IL6 remained significant predictors	maternal anemia (as a proxy for maternal nutrition)
Wren-Atioloa et al. Guatemala, 2021 (LMIC)	Cohort<br><br>(140 enrolled and analysed)	<6 weeks, 4-6 months	<6 weeks, 4-6 months	Concentrations in HM	Head circumference, weight, length, WAZ, LAZ, and HCAZ<br><br>(WHO standards)	(-) association between milk IL1β and LAZ (standardized β = −0.201, P = 0.040) before 6 wk<br>(+) association between IL-1β and daily rate of increase in infant length from early to established lactation (standardized β = 0.281, P = 0.010)	Indicators of Sub-Clinical mastitis and breast inflammation, fecal oral contamination, and breastfeeding practices
**IL-2, 1 study/article**							
Saso et al. The Gambia, 2018 (LIC)	Subset of larger cohort.<br><br>n=100 mother-infant pairs	Birth (colostrum), day 60-89 postpartum	Birth, day 60-89 postpartum	Concentrations in HM	Change in WAZ between birth and final visit, WAZ at final visit.	(+) Association between IL6 and WAZ at final visit (Adjusted)<br>(-) Association between TNF-alpha and WAZ at final visit (Adjusted)<br><br>Cytokine levels in mature breast milk were weakly predictive of poor infant growth, possibly reflecting a “read-out” of suboptimal maternal health and nutrition. When adjusted for maternal anemia (as a proxy for maternal nutrition), TNFα and IL6 remained significant predictors ($p < 0.05$). IL6 R^2: 0.17. p<001. TNFa R^2 0.17, $p < 0.01$.	maternal anemia (as a proxy for maternal nutrition)
**IL-13, 1 study/article**							
Saso et al. The Gambia, 2018 (LIC)	Subset of larger cohort.<br><br>n=100 mother-infant pairs	Birth (colostrum), day 60-89 postpartum	Birth, day 60-89 postpartum	Concentrations in HM	Change in WAZ between birth and final visit, WAZ at final visit.	(No) Associations between IL-13 and infant growth<br><br>Cytokine levels in mature breast milk were weakly predictive of poor infant growth, possibly reflecting a “read-out” of suboptimal maternal health and nutrition. When adjusted for maternal anemia (as a proxy for maternal nutrition), TNFα and IL6 remained significant predictors ($p < 0.05$). IL6 R^2: 0.17. p<001. TNFa R^2 0.17, $p < 0.01$.	maternal anemia (as a proxy for maternal nutrition)
**IL-4, 2 studies**							
Durilova et al. Czech Republic, 2010 (HIC)	Cross-sectional (20 enrolled & analyzed)	2 - 27 weeks (EC group), 12 weeks (control)	Not recorded	Concentrations in HM	Body weight	(+) Association between IL-4 and body weight ($r = 0.345$, $p = 0.029$)	None reported
Saso et al. The Gambia, 2018 (LIC)	Subset of larger cohort.<br><br>n=100 mother-infant pairs	Birth (colostrum), day 60-89 postpartum	Birth, day 60-89 postpartum	Concentrations in HM	Change in WAZ between birth and final visit, WAZ at final visit.	(No) Associations between IL-4 and infant growth<br><br>Cytokine levels in mature breast milk were weakly predictive of poor infant growth, possibly reflecting a “read-out” of suboptimal maternal health and nutrition. When adjusted for maternal anemia (as a proxy for maternal nutrition), TNFα and IL6 remained significant predictors ($p < 0.05$). IL6 R^2: 0.17. p<001. TNFa R^2 0.17, $p < 0.01$.	maternal anemia (as a proxy for maternal nutrition)
**IL-6, 8 studies/articles**							
Durilova et al. Czech Republic, 2010 (HIC)	Cross-sectional (20 enrolled & analyzed)	2 - 27 weeks (EC group), 12 weeks (control)	Not recorded	Concentrations in HM	Body weight	(-) Association between IL-6 and body weight ($r = -0.370$, $p = 0.019$)	None reported
Enstad et al. USA, 2019 (HIC)	Longitudinal (40 enrolled & analyzed)	1, 4 months	1, 2, 3, 4, 5, 6, 7 months	Concentrations in HM	Weight z-score, length z-score, BMI z-score, % fat mass, % lean mass, infant growth trajectory from month 1 to month 7	(+) Association between cytokines and weight z-scores at birth<br>(-) Association between cytokines and weight z-scores between 1-2 months<br>(+) Association between cytokines and weight z-scores after 2 months	Race, infant age at time of growth measurement, baseline infant measurement (measured at birth or month 1), sex
Fields et al. USA, 2017 (HIC)	Longitudinal (37 enrolled, 30 analyzed at 6 months)	1 month, 6 months	1 month, 6 months	Concentrations in HM	Weight, length, % fat, total fat mass, total fat free mass, trunk fat mass	(No) Association between month 1 IL-6 levels and month 6 body length, % fat, total fat mass, total fat-free mass and trunk fat mass	Infant sex, pregravid maternal BMI category (normal weight, overweight, obese), stage of lactation (1 month vs. 6 months)
Nuss et al. USA, 2019 (HIC)	Cross-sectional (33 enrolled & analyzed)	One sample, between 4-8 weekspostpartum	One sample, between 4-8 weekspostpartum	Concentrations in HM	Weight, length, head circumference, % fat mass	(-) Association between IL-6 and infant weight<br>(-) Association between IL-6 and infant head circumference<br>(-) Association between IL-6 and % fat mass<br>(-) Association between IL-6 and Infant length	Infant age at visit

Table S5. Characteristics and results of included studies reporting on human milk <u>immunomodulatory components</u> and infant anthropometrics - organized by component.

Authors, country, publication year (income setting)	Design and participants	Timing of milk sampling	Timing of infant anthropometrics	Estimated intake or HM concentration*	Anthropometric outcome measures and standards	Associations**	Major confounders considered
Ortiz-Andrellucchi et al.<br>Spain, 2008<br>(HIC)	RCT.<br>104 mothers (45assignemd to placebo/59 assigned to intreatment)<br>39 mothers analysed from placebo group/54 mothers analysed from treatment group<br>38 children analyzed from placebo group /51 children analyzed from treatment group	72 days, 10 days, and 45 days postpartum	Birth, 2 months, 6 months	Concentrations in HM	Infant weight	No significant differences between groups in relation to weight (data not shown).	None reported
Saso et al.<br>The Gambia, 2018<br>(LIC)	Subset of larger cohort.<br><br>n=100 mother-infant pairs	Birth (colostrum), day 60-89 postpartum	Birth, day 60-89 postpartum	Concentrations in HM	Change in WAZ between birth and final visit, WAZ at final visit.	(+) Association between IL6 and WAZ at final visit (Adjusted)<br><br>Cytokine levels in mature breast milk were weakly predictive of poor infant growth, possibly reflecting a “read-out” of suboptimal maternal health and nutrition. When adjusted for maternal anemia (as a proxy for maternal nutrition), TNFα and IL6 remained significant predictors (p < 0.05). IL6 R^2: 0.17. p<001. TNFa R^2 0.17, p < 0.01.	maternal anemia (as a proxy for maternal nutrition)
Sims et al.<br>USA, 2020<br>(HIC)	Longitudinal<br>(284 enrolled, 174 analyzed)	Postnatal age 0.5, 1, 2, 3, 4, 5, 6, and 9 months	Postnatal age 0.5, 1, 2, 3, 4, 5, 6, and 9 months	Estimated intake and concentrations in HM, but associations only found/reported for estimated intake.	Weight, length, LFA z-score, WFA z-score, WFL z-score, fat mass, fat-free mass, FMI, FFMI	(No) Association between IL-6 and infant anthropometrics	Infant sex, feeding mode (exclusive vs. mixed)
Wren-Atioloa et al.<br>Guatemala, 2021<br>(LMIC)	Cohort<br><br>(140 enrolled and analysed)	<6 weeks, 4-6 months	<6 weeks, 4-6 months	Concentrations in HM	Head circumference, weight, length, WAZ, LAZ, and HCAZ<br><br>(WHO standards)	(No) Association between IL-6 and infant anthropometrics	Indicators of Sub-Clinical mastitis and breast inflammation, fecal oral contamination, and breastfeeding practices
**IL-8, 4 studies/articles**							
Enstad et al.<br>USA, 2019<br>(HIC)	Longitudinal<br>(40 enrolled & analyzed)	1, 4 months	1, 2, 3, 4, 5, 6, 7 months	Concentrations in HM	Weight z-score, length z-score, BMI z-score, % fat mass, % lean mass, infant growth trajectory from month 1 to month 7	(+) Association between IL-8 and infant BMI z-score at V7<br>(+) Association between cytokines and weight z-scores at birth<br>(-) Association between cytokines and weight z-scores between 1-2 months<br>(+) Association between cytokines and weight z-scores after 2 months	Race, infant age at time of growth measurement, baseline infant measurement (measured at birth or month 1), sex
Ortiz-Andrellucchi et al.<br>Spain, 2008<br>(HIC)	RCT.<br>104 mothers (45assignemd to placebo/59 assigned to intreatment)<br>39 mothers analysed from placebo group/54 mothers analysed from treatment group<br>38 children analyzed from placebo group /51 children analyzed from treatment group	72 days, 10 days, and 45 days postpartum	Birth, 2 months, 6 months	Concentrations in HM	Infant weight	No significant differences between groups in relation to weight (data not shown).	None reported
Sims et al.<br>USA, 2020<br>(HIC)	Longitudinal<br>(284 enrolled, 174 analyzed)	Postnatal age 0.5, 1, 2, 3, 4, 5, 6, and 9 months	Postnatal age 0.5, 1, 2, 3, 4, 5, 6, and 9 months	Estimated intake and concentrations in HM, but associations only found/reported for estimated intake.	Weight, length, LFA z-score, WFA z-score, WFL z-score, fat mass, fat-free mass, FMI, FFMI	(No) Associations between IL-8 and infant anthropometrics	Infant sex, feeding mode (exclusive vs. mixed)
Wren-Atioloa et al.<br>Guatemala, 2021<br>(LMIC)	Cohort<br><br>(140 enrolled and analysed)	<6 weeks, 4-6 months	<6 weeks, 4-6 months	Concentrations in HM	Head circumference, weight, length, WAZ, LAZ, and HCAZ<br><br>(WHO standards)	(+) associations between IL-8 and HCAZ (standardized β = 0.255, P = 0.014)	Indicators of Sub-Clinical mastitis and breast inflammation, fecal oral contamination, and breastfeeding practices
**IL-10, 3 studies/articles**							
Durilova et al.<br>Czech Republic, 2010<br>(HIC)	Cross-sectional<br>(20 enrolled & analyzed)	2 - 27 weeks (EC group), 12 weeks (control)	Not recorded	Concentrations in HM	Body weight	(No) Associations between IL-10 and infant anthropometrics	None reported
Ortiz-Andrellucchi et al.<br>Spain, 2008<br>(HIC)	RCT.<br>104 mothers (45assignemd to placebo/59 assigned to intreatment)<br>39 mothers analysed from placebo group/54 mothers analysed from treatment group<br>38 children analyzed from placebo group /51 children analyzed from treatment group	72 days, 10 days, and 45 days postpartum	Birth, 2 months, 6 months	Concentrations in HM	Infant weight	No significant differences between groups in relation to weight (data not shown).	None reported
Saso et al.<br>The Gambia, 2018<br>(LIC)	Subset of larger cohort.<br><br>n=100 mother-infant pairs	Birth (colostrum), day 60-89 postpartum	Birth, day 60-89 postpartum	Concentrations in HM	Change in WAZ between birth and final visit, WAZ at final visit.	(No) Associations between IL-10 and infant anthropometrics	maternal anemia (as a proxy for maternal nutrition)
**IL-12, 2 studies/articles**							
Ortiz-Andrellucchi et al.<br>Spain, 2008<br>(HIC)	RCT.<br>104 mothers (45assignemd to placebo/59 assigned to intreatment)<br>39 mothers analysed from placebo group/54 mothers analysed from treatment group<br>38 children analyzed from placebo group /51 children analyzed from treatment group	72 days, 10 days, and 45 days postpartum	Birth, 2 months, 6 months	Concentrations in HM	Infant weight	No significant differences between groups in relation to weight (data not shown).	None reported
Saso et al.<br>The Gambia, 2018<br>(LIC)	Subset of larger cohort.<br><br>n=100 mother-infant pairs	Birth (colostrum), day 60-89 postpartum	Birth, day 60-89 postpartum	Concentrations in HM	Change in WAZ between birth and final visit, WAZ at final visit.	(No) Association between IL-12 and infant anthropometrics	maternal anemia (as a proxy for maternal nutrition)

Table S5. Characteristics and results of included studies reporting on human milk immunomodulatory components and infant anthropometrics - organized by component.

Authors, country, publication year (income setting)	Design and participants	Timing of milk sampling	Timing of infant anthropometrics	Estimated intake or HM concentration*	Anthropometric outcome measures and standards	Associations**	Major confounders considered
**IL-17, 1 study/article**							
Durilova et al.<br>Czech Republic, 2010<br>(HIC)	Cross-sectional<br>(20 enrolled & analyzed)	2 - 27 weeks (EC group), 12 weeks (control)	Not recorded	Concentrations in HM	Body weight	(No) Associations between IL-17 and infant anthropometrics	None reported
**IL-18, 1 study/article**							
Durilova et al.<br>Czech Republic, 2010<br>(HIC)	Cross-sectional<br>(20 enrolled & analyzed)	2 - 27 weeks (EC group), 12 weeks (control)	Not recorded	Concentrations in HM	Body weight	(No) Associations between IL-18 and infant anthropometrics	None reported
**IL-23, 1 study**							
Durilova et al.<br>Czech Republic, 2010<br>(HIC)	Cross-sectional<br>(20 enrolled & analyzed)	2 - 27 weeks (EC group), 12 weeks (control)	Not recorded	Concentrations in HM	Body weight	(No) Associations between IL-23 and infant anthropometrics	None reported
**TNF-a, 6 studies/articles**							
Fields et al.<br>USA, 2017<br>(HIC)	Longitudinal<br>(37 enrolled, 30 analyzed at 6 months)	1 month, 6 months	1 month, 6 months	Concentrations in HM	Weight, length, % fat, total fat mass, total fat free mass, trunk fat mass	(No) Association between month 1 TNF-a levels and month 6 body length, % fat, total fat mass, total fat-free mass and trunk fat mass	Infant sex, pregravid maternal BMI category (normal weight, overweight, obese), stage of lactation (1 month vs. 6 months)
Nuss et al.<br>USA, 2019<br>(HIC)	Cross-sectional<br>(33 enrolled & analyzed)	One sample, between 4-8 weekspostpartum	One sample, between 4-8 weekspostpartum	Concentrations in HM	Weight, length, head circumference, % fat mass	(+) Association between TNF-a and infant weight<br>(-) Association between TNF-a and infant head circumference<br>(+) Association between TNF-a and % fat mass<br>(No) Association between TNF-a and Infant length	Infant age at visit
Ortiz-Andrellucchi et al.<br>Spain, 2008<br>(HIC)	RCT.<br>104 mothers (45assignemd to placebo/59 assigned to intreatment)<br>39 mothers analysed from placebo group/54 mothers analysed from treatment group<br>38 children analyzed from placebo group /51 children analyzed from treatment group	72 days, 10 days, and 45 days postpartum	Birth, 2 months, 6 months	Concentrations in HM	Infant weight	No significant differences between groups in relation to weight (data not shown).	None reported
Saso et al.<br>The Gambia, 2018<br>(LIC)	Subset of larger cohort.<br><br>n=100 mother-infant pairs	Birth (colostrum), day 60-89 postpartum	Birth, day 60-89 postpartum	Concentrations in HM	Change in WAZ between birth and final visit, WAZ at final visit.	(-) Association between TNF-alpha and WAZ at final visit (Adjusted)<br><br>Cytokine levels in mature breast milk were weakly predictive of poor infant growth, possibly reflecting a "read-out" of suboptimal maternal health and nutrition. When adjusted for maternal anemia (as a proxy for maternal nutrition), TNFα and IL6 remained significant predictors ($p < 0.05$). IL6 R^2: 0.17. p<001. TNFa R^2 0.17, $p < 0.01$.	maternal anemia (as a proxy for maternal nutrition)
Sims et al.<br>USA, 2020<br>(HIC)	Longitudinal<br>(284 enrolled, 174 analyzed)	Postnatal age 0.5, 1, 2, 3, 4, 5, 6, and 9 months	Postnatal age 0.5, 1, 2, 3, 4, 5, 6, and 9 months	Estimated intake and concentrations in HM, but associations only found/reported for estimated intake.	Weight, length, LFA z-score, WFA z-score, WFL z-score, fat mass, fat-free mass, FMI, FFMI	(No) Associations between TNF-a and infant anthropometrics	Infant sex, feeding mode (exclusive vs. mixed)
Wren-Atioloa et al.<br>Guatemala, 2021<br>(LMIC)	Cohort<br><br>(140 enrolled and analysed)	<6 weeks, 4-6 months	<6 weeks, 4-6 months	Concentrations in HM	Head circumference, weight, length, WAZ, LAZ, and HCAZ<br><br>(WHO standards)	(No) Associations between TNF-a and infant anthropometrics	Indicators of Sub-Clinical mastitis and breast inflammation, fecal oral contamination, and breastfeeding practices
**TGF-beta, 4 studies/articles**							
Alsharnoubi et al., Egypt, 2019 (LMIC)	Cross-sectional. 84 mother-infant dyads	71.5 +/- 61.4 days	71.5 +/- 61.4 days	Concentrations in HM	Weight, length, head circumference, postnatal weight gain, TSF	A comparative study on different infant body weight and length groups showed that TGF-b1 was much higher in infants with average weight.	Mother's age, specific food, contraceptive use, infant's age [days], infant's weight, infant's length
Durilova et al.<br>Czech Republic, 2010<br>(HIC)	Cross-sectional<br>(20 enrolled & analyzed)	2 - 27 weeks (EC group), 12 weeks (control)	Not recorded	Concentrations in HM	Body weight	(No) Associations between TNF-b and infant anthropometrics	None reported
Ortiz-Andrellucchi et al.<br>Spain, 2008<br>(HIC)	RCT.<br>104 mothers (45assignemd to placebo/59 assigned to intreatment)<br>39 mothers analysed from placebo group/54 mothers analysed from treatment group<br>38 children analyzed from placebo group /51 children analyzed from treatment group	72 days, 10 days, and 45 days postpartum	Birth, 2 months, 6 months	Concentrations in HM	Infant weight	No significant differences between groups in relation to weight (data not shown).	None reported
Saso et al.<br>The Gambia, 2018<br>(LIC)	Subset of larger cohort.<br><br>n=100 mother-infant pairs	Birth (colostrum), day 60-89 postpartum	Birth, day 60-89 postpartum	Concentrations in HM	Change in WAZ between birth and final visit, WAZ at final visit.	(No) Associations between TNF-b and infant anthropometrics	None reported
**Lysozyme, 2 studies/3 articles**							
Gridneva et al.,<br>Australia. 2018 (HIC)<br><br>Gridneva et al.,<br>Australia. 2021 (HIC)	Cohort. 20 mother-infant dyads were enrolled	2, 5, 9, 12 months	2, 5, 9, 12 months	Estimated intake and concentration	Infant length, weight, BMI, head circumference, infant body composition, infant abdominal adiposity<br><br>(WHO standards)	(No) Associations between lysozyme and infant anthropometrics	None reported

Table S5. Characteristics and results of included studies reporting on human milk immunomodulatory components and infant anthropometrics - organized by component.

Authors, country, publication year (income setting)	Design and participants	Timing of milk sampling	Timing of infant anthropometrics	Estimated intake or HM concentration*	Anthropometric outcome measures and standards	Associations**	Major confounders considered
Larsson et al. Denmark, 2018 (HIC)	Longitudinal (59 enrolled, 30 analyzed)	First visit: 5-6.5 months old; Second visit: 9 months (+/- 2 weeks); Third visit for HW-group only: 18 months +/- 4 weeks	First visit: 5-6.5 months old; Second visit: 9 months (+/- 2 weeks); Third visit for HW-group only: 18 months +/- 4 weeks	Estimated intake and concentration in HM but associations determined using concentrations in milk	Weight, recumbent length, mid-upper-arm circumference, head circumference, lower leg circumference, recumbent waist and thorax circumference, triceps and subscapular skinfold thickness, WAZ, LAZ, BAZ, triceps skinfold for age z-score (TSFZ), subscapular skinfold-for-age z-score (SSFZ)	(No) Association between milk concentrations of adiponectin, leptin, lysozyme, sIgA, lactoferrin and infant's anthropometry or change in z-scores from birth to the 5-month visit	Maternal fasting time, infant sex
**Lactoferrin, 3 studies/4 articles**							
Gridneva et al., Australia. 2018 (HIC)<br><br>Gridneva et al., Australia. 2021 (HIC)	Cohort. 20 mother-infant dyads were enrolled	2, 5, 9, 12 months	2, 5, 9, 12 months	Estimated intake and concentration	Infant length, weight, BMI, head circumference, infant body composition, infant abdominal adiposity<br><br>(WHO standards)	(-) Association between lactoferrin concentration and infant visceral depth<br><br>(no other significant associations)	None reported
Jorgensen et al. Malawi, 2020 (LIC)	Longitudinal (1391 enrolled, 659 samples collected, 647 analyzed for HMOs and 637 analyzed for protein)	6 months postpartum	6, 12 months	Concentration in HM	Change in LAZ from 6-12 months, chagnein WAZ from 6-12 months, change in WLZ from 6-12 months, change in HCZ from 6-12 months	For secretors + nonsecretors combined:<br>In primary analyses:<br>(No) association between abundance of groups of HMOs or concentrations of IgA, lactalbumin, or lactoferrin and infant growth indicators.<br>.	Secretor status, baseline age, BMI, parity, education, food security, HIV status, Hb, household assets, residential location, season at time of sample collection, intervention group (consumed iron and folic acid during pregnancy, or consumed multiple micronutrient capusle during pregnancy and up to 6 months postpartum, or consumed a lipid-based nutrient supplement during pregnancy and up to 6 months postpartum), infant sex
Larsson et al. Denmark, 2018 (HIC)	Longitudinal (59 enrolled, 30 analyzed)	First visit: 5-6.5 months old; Second visit: 9 months (+/- 2 weeks); Third visit for HW-group only: 18 months +/- 4 weeks	First visit: 5-6.5 months old; Second visit: 9 months (+/- 2 weeks); Third visit for HW-group only: 18 months +/- 4 weeks	Estimated intake and concentration in HM but associations determined using concentrations in milk	Weight, recumbent length, mid-upper-arm circumference, head circumference, lower leg circumference, recumbent waist and thorax circumference, triceps and subscapular skinfold thickness, WAZ, LAZ, BAZ, triceps skinfold for age z-score (TSFZ), subscapular skinfold-for-age z-score (SSFZ)	(No) Association between milk concentrations of adiponectin, leptin, lysozyme, sIgA, lactoferrin and infant's anthropometry or change in z-scores from birth to the 5-month visit	Maternal fasting time, infant sex
**sIgA, 2 studies/ 3 articles**							
Gridneva et al., Australia. 2018 (HIC)<br><br>Gridneva et al., Australia. 2021 (HIC)	Cohort. 20 mother-infant dyads were enrolled	2, 5, 9, 12 months	2, 5, 9, 12 months	Estimated intake and concentration	Infant length, weight, BMI, head circumference, infant body composition, infant abdominal adiposity<br><br>(WHO standards)	(No) Associations between sIgA and infant anthropometrics	None reported
Larsson et al. Denmark, 2018 (HIC)	Longitudinal (59 enrolled, 30 analyzed)	First visit: 5-6.5 months old; Second visit: 9 months (+/- 2 weeks); Third visit for HW-group only: 18 months +/- 4 weeks	First visit: 5-6.5 months old; Second visit: 9 months (+/- 2 weeks); Third visit for HW-group only: 18 months +/- 4 weeks	Estimated intake and concentration in HM but associations determined using concentrations in milk	Weight, recumbent length, mid-upper-arm circumference, head circumference, lower leg circumference, recumbent waist and thorax circumference, triceps and subscapular skinfold thickness, WAZ, LAZ, BAZ, triceps skinfold for age z-score (TSFZ), subscapular skinfold-for-age z-score (SSFZ)	(No) Association between milk concentrations of adiponectin, leptin, lysozyme, sIgA, lactoferrin and infant's anthropometry or change in z-scores from birth to the 5-month visit	Maternal fasting time, infant sex
**MDA, 2 studies/articles**							
Enstad et al. USA, 2019 (HIC)	Longitudinal (40 enrolled & analyzed)	1, 4 months	1, 2, 3, 4, 5, 6, 7 months	Concentrations in HM	Weight z-score, length z-score, BMI z-score, % fat mass, % lean mass, infant growth trajectory from month 1 to month 7	(+) Association between cytokines and weight z-scores at birth<br>(-) Association between cytokines and weight z-scores between 1-2 months<br>(+) Association between cytokines and weight z-scores after 2 months	Race, infant age at time of growth measurement, baseline infant measurement (measured at birth or month 1), sex

Table S5. Characteristics and results of included studies reporting on human milk immunomodulatory components and infant anthropometrics - organized by component.

Authors, country, publication year (income setting)	Design and participants	Timing of milk sampling	Timing of infant anthropometrics	Estimated intake or HM concentration*	Anthropometric outcome measures and standards	Associations**	Major confounders considered
Nikniaz et al. Iran, 2013 (LMIC)	RCT. 80 mothers enrolled(Reviewer 1 just wrote N=80) . 75 completed the study, providing anthropometrics and HM samples	30 days post-intervention	Pre and post intervention	Concentrations in HM	Infant BMI, WAZ, HAZ, Head Circumference	No significant association was found between MDA levels and weight for age Z-score of infants and TAC.	Infant birth weight, Maternal BMI , maternal daily energy intake

*Values reported as mean ± SD or median (IQR). **"No (assumed) associations = unreported associations assumed to be no association.

Abbreviations: BF, breastfeeding; HIC, high income countries; mo, months; HM, human milk; LMIC, low and middle income countries; NCHS, National Center for Health Statistics; RCT, randomized controlled trial; SCM, subclinical mastitis; WHO, World Health Organization; wks, weeks

Anthropometrics: BMI, body mass indez; HAZ, height for age z-score; HC, head circumference; HCAZ, head circumference z-score; LAZ, length for age Z-score; LFA, length for age; WAZ, weight for age z-score; WFA, weight for age; WLZ, weight-for-length z-score

Components: cGP, Cyclic glycine-proline; sCD14, Soluble cluster of differentiation 14; GLP, Glucagon-like peptide; IFLNH-I, fucosyl-para-lacto-N-hexaose I; IGF-1, Insulin-like growth factor 1; IL (1,4,6,α, β), Interleukin; LNFPI, PYY, Peptide YY (also known as peptide tyrosine tyrosine); TFLNH, Trifucosyllacto-N-hexaose; TNF-α, Tumor necrosis factor – alpha; TGF-β, Transforming growth factor – beta.
