# Supplementary material for: Human Milk Bioactive Components and Child Growth and Body Composition in the First 2 Years: A Systematic Review
Source: Adv Nutr. 2023 Oct 4;15(1):100127. doi: 10.1016/j.advnut.2023.09.015 (PMC10831900; doi:10.1016/j.advnut.2023.09.015)
Supplement: Multimedia component 2 [file mmc2.docx]

# Appendix A

Medline Search

1 exp Breast feeding/ or HM/ or exp Lactation/ or Colostrum/ (90647)

2 (breast-feed$ or breastfeed$ or breast-fed$ or breastfed$ or breastmilk$ or colostrum).kw,tw. (50890)

3 (milk adj2 (mother$ or maternal or express$ or human or breast$ or share$ or sharing)).kw,tw. (24630)

4 ((nursing or lactating) adj2 (mother$ or maternal or feed$ or infant$ or milk)).kw,tw. (4994)

5 or/1-4 [Breast milk search concept] (116976)

6 exp body weight/ or body composition/ or exp "body weights and measures"/ or growth/ or exp body size/ or growth disorders/ or exp anthropometry/ or Failure to Thrive/ (725377)

7 ((body or muscle) adj2 (composition$ or fat$ or mass$ or size$ or height$ or length$)).kw,tw. (315242)

8 (weight or grow$ or BMI or height or over-weight or overweight or underweight or under-weight or obesity or obese or anthropomet$ or (physical adj1 develop$)).tw,kw. (2833561)

9 or/6-8 [Growth Search Concept] (3206373)

10 Infants/ or Infant, Newborn/ or Infant Health/ or (infant$ or babies or baby$ or newborn or new-born or perinat$ or neonat$).kw,tw. (1433063)

11 exp Dietary Proteins/ or exp Dietary Carbohydrates/ or exp Lipids/ (1287487)

12 exp "amino acids, peptides, and proteins"/ or exp carbohydrates/ or nutrients/ (7763295)

13 (macronutri$ or macro-nutri$ or carbohydrate$1 or fat$ or lactose$ or galactose$ or casein$ or phosphoprotein$ or lipoprotein$ or protein$ or aminoacid$ or taurine$ or lysine$ or tyrosine$ or amino-acid$ or triacylglyceride$ or triglyceride$ or triacylglycerol$ or diglyceride$ or diacylglycerol$ or diacylglyceride$ or monoacylglyceride$ or monoglyceride$ or acylglycerol$ or lipid$ or phospholipid$ or fattyacid$ or acid$ or DHA or EPA or cholesterol$ or LCPUFA$ or PUFA$ or omega-3 or omega-6 or glycoprotein$ or glycolipid$ or glucose$ or disaccharide$).kw,tw. (5737284)

14 or/11-13 [Macronutrient search concept] (10146645)

15 5 and 9 and 10 and 14 (7079)

16 exp Micronutrients/ or Calcium, Dietary/ or Phosphorus, Dietary/ or magnesium/ or exp Vitamin A/ or carotenoids/ or choline/ or Iron, Dietary/ or exp Elements/ or exp Folic Acid/ or Dietary Potassium/ (2001860)

17 (micro-nutri$ or micronutri$ or provitamin$ or pro-vitamin$ or previtamin$ or zinc or calcium$ or phosphorus$ or magnesium$ or iodine$ or selenium$ or carotenoid$ or thiamine$ or riboflavin$ or folate$ or choline$ or iron$ or niacin or nicotinamide$ or mineral$ or retinol$ or potassium).kw,tw. (1228280)

18 (vitamin adj1 (a$1 or b$2 or c$1 or d$1 or e$1)).tw,kw. (161743)

19 or/16-18 [Micronutrient Search] (2733776)

20 5 and 9 and 10 and 19 (2885)

21 exp immunoglobulins/ or exp oligosaccharides/ or exp Hormones/ (2270362)

22 (bio-active$ or bioactive$ or immune-globulin$ or immunoglobulin$ or antibod$ or anti-bod$ or monoligosacchari$ or mono-oligosacchari$ or monosacchari$ or oligosacchari$ or HMO or fucosyllactose$ or sialyllactose$ or ig$2 or cytokine$ or interleukin$ or interferon-g or lactoferrin$ or lactotransferrin$ or osteopontin$ or bone-sialoprotein or hormone$ or leptin$ or insulin$ or adiponectin$ or ghrelin$ or "tumo?r necrosis factor-a" or TNF-a$).kw,tw. (2386179)

23 (transforming-growth-factor$ or tumo?r-growth-factor$ or TGF$ or epidermal-growth-factor$ or urogastrone$).kw,tw. (166992)

24 or/21-23 [Bioactive Search] (3637852)

25 5 and 9 and 10 and 24 (3101)

26 15 or 20 or 25 (8686)

27 26 not (exp Animals/ not humans.sh.) (7205)

28 limit 27 to english language (6683)

29 limit 28 to yr="1980 -Current" (6365)
